# Chemically Recyclable Polyester Thermosets from Activated Adipic Acid and Renewable Polyols

**DOI:** 10.1002/cssc.202500880

**Published:** 2025-09-01

**Authors:** Davide Rigo, Matteo Lorenzon, Jonas Simon, Bennett Addison, Alvise Perosa, Maurizio Selva

**Affiliations:** ^1^ Department of Molecular Sciences and Nanosystems Ca’ Foscari University of Venice Via Torino 155 30172 Venice Italy; ^2^ Department of Chemistry Institute of Chemistry of Renewable Resources University of Natural Resources and Life Sciences Vienna (BOKU) Konrad‐Lorenz‐Strasse 24 3430 Tulln Austria; ^3^ Renewable Resources and Enabling Sciences Institute of Chemistry of Renewable Resources National Renewable Energy Laboratory Golden Colorado 80401 United States

**Keywords:** bioplastics, chemical recycling, isopropenyl acetate, solvent‐free, upcycling

## Abstract

This study outlines a method for producing chemically recyclable crosslinked polyesters using renewable polyols**—**glycerol and sorbitol**—**combined with adipic acid (AA), which is transformed/activated into a polyanhydride mixture prior to use. A three‐step procedure has been designed: 1) an acid‐catalyzed reaction of AA with nontoxic isopropenyl acetate or acetic anhydride to form a crosslinking mixture (CLM) made of adipic‐acetic mixed polyanhydrides; 2) a solvent‐ and additive‐free process where glycerol or sorbitol, or a combination thereof, is reacted with the CLM to achieve a prepolymer, and 3) a casting/molding of the liquid viscous prepolymer to yield a thermoset as the end product. Different thermosets (eight examples) are prepared by changing the reagents ratio. These solids are thoroughly characterized by tensile tests, DMA, high‐resolution magic angle spinning and solid‐state NMR, thermal gravimetric analysis, DSC, and fourier transformed infra red (FT‐IR) spectroscopy. The formation of cross‐linked polyesters is confirmed in all cases, but mechanical properties varied significantly from one specimen to another. Interestingly, a tensile strength up to 18 MPa**—**approximately an order of magnitude higher than similar polymers**—**is achieved when sorbitol and the CLM are used in a 1:1 wt% ratio. The chemical recycle of the resulting polymers is achieved via methanolysis with quantitative recovery of the monomeric units.

## Graphical abstract

Closed‐loop chemically recyclable polyester thermosets were synthesized using glycerol and sorbitol and adipic acid as the monomers. Adipic acid was converted/activated into a mixture of polyanhydrides via a benign procedure prior to polymerization. A tensile strength up to 18 MPa was achieved. Methanolysis provided quantitative recovery of the monomeric units which could then be repolymerized.

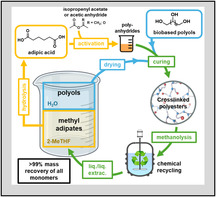

## 1 Introduction

**Figure 1:**
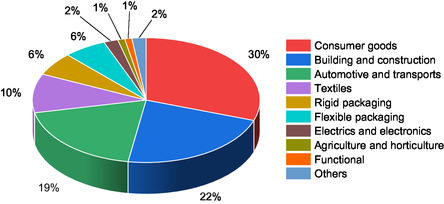
Shares of biobased polymers in different market segments in 2023. Data.^[^ ^1^ ^]^

The global production of bio‐based plastics (BBPs) is limited compared to that of fossil‐based polymers, not exceeding 2% (≈7.000 tonn y^−1^) of all plastics available today.^[^ ^1^, ^2^ ^]^ Currently, bio‐based polymers that are either already produced commercially or expected to become commercially available soon stand at just over a dozen of compounds, including, high‐ and low‐density polyethylene, polyethylene‐terephthalate and furanoate (PET and PEF), polycarbonate (PC), polybutylene succinate (PBS), polylactic acid (PLA), polyhydroxyalkanoate (PHA), thermoplastic starch (TPS), and thermoplastic polyurethane (TPU).^[^ ^3^, ^4^ ^]^ The segments for the applications of these materials are very different and are summarized in **Figure** **1**. Other less widespread BBPs have been synthesized starting from terpenes and terpenoids,^[^ ^5^, ^6^ ^]^ fatty oils,^[^ ^7^, ^8^ ^]^ and lignin.^[^ ^9^, ^10^ ^]^

The sector of BBPs, however, is expected to expand significantly and gain an increasingly relevant market share.^[^ ^1^ ^]^

The design/development of existing or new BBPs implies remarkable techno‐economic challenges: bio‐based materials not only must meet severe requirements in terms of properties and production costs competitive to their fossil‐based counterparts, but they should be as compatible as possible with existing recycling processes or be biodegradable/compostable.^[^ ^2^, ^11^ ^]^

**Figure 2:**
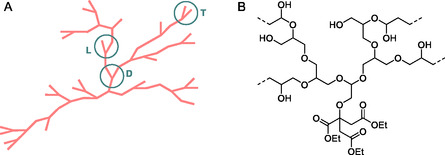
A) Schematic view of a hyperbranched polymer: linear defects (L), dendritic (D) and terminal (T) units and B) a biomass‐based hyperbranched polyol from triethyl citrate and glycidol.

An innovative and rather unexplored area in this context is represented by renewable highly branched polymers (r‐HBPs). In general, HBPs are described as dispersed materials with a structure based on a randomly assembled pattern of chemical bonds where branched (crosslinked) units and/or even dendritic ones coexist with unreacted terminal/linear repeating segments.^[^ ^12^ ^]^ As an example, **Figure** **2** depicts a schematic representation of an HBP (A, left) highlighting randomly distributed linear defects (L), dendritic (D), and terminal (T) units in the structure, and an example of a biomass‐based hyperbranched polyol (B, right).^[^ ^13^, ^14^ ^]^

The architecture of Figure 2 confers to HBPs unique physical and chemical properties^[^ ^12^, ^15^, ^16^, ^17^, ^18^, ^19^, ^20^, ^21^, ^22^ ^]^ as demonstrated for a variety of polyesters,^[^ ^23^ ^]^ polyamides,^[^ ^24^ ^]^ polyphenylenes,^[^ ^15^, ^22^ ^]^ and polyurethanes.^[^ ^25^ ^]^ For the specific interest in this work, the focus has been placed on bio‐based polyesters that, more than other polymers, are fast emerging for their tunable properties and wide range of application areas.^[^ ^26^ ^]^ The synthesis of such materials requires polyfunctional building blocks. Sugar alcohols such as glycerol and sorbitol represent excellent nucleophilic partners for the polymerization reaction,^[^ ^27^, ^28^, ^29^, ^30^ ^]^ while polyacids act as electrophilic cross‐linking agents.^[^ ^31^ ^]^ Particularly, adipic acid (AA), which is recognized from IEA (International Energy Agency) as the most important dicarboxylic acid,^[^ ^32^ ^]^ is often a natural monomer candidate to the scope. It should be reminded here that both glycerol and sorbitol have consolidated positions in the list of the top platform chemicals from the biodiesel industry^[^ ^33^ ^]^ (glycerol), and from the hydrogenation of glucose^[^ ^34^ ^]^ or cellulose^[^ ^35^ ^]^ (sorbitol). These polyols display one of the largest potentials among bio‐based molecules for further conversions to value‐added chemicals or materials.^[^ ^36^, ^37^ ^]^ Adipic acid, instead, is still primarily manufactured from petrochemical feedstock, though an ongoing massive research promises to introduce bio‐based AA soon into the market via the treatment of biomass (e.g., lignin and other agro‐waste) with three major pathways, including chemical catalytic processes, indirect fermentation with chemical conversion, and direct fermentation.^[^ ^32^, ^38^, ^39^ ^]^

Several examples of the polyesterification of AA with glycerol,^[^ ^28^, ^40^, ^41^, ^42^, ^43^, ^44^, ^45^, ^46^, ^47^, ^48^, ^49^, ^50^ ^]^ or sorbitol,^[^ ^51^, ^52^, ^53^, ^54^ ^]^ or mixtures of them^[^ ^27^, ^55^ ^]^ have been already reported in the literature (Table S1). Strategies based on enzymatic catalysis (mainly lipases),^[^ ^27^, ^29^, ^43^, ^47^, ^51^, ^55^ ^]^ and metal‐/organo‐catalyzed protocols^[^ ^41^, ^54^ ^]^ typically require temperatures in the range of 60–100 °C and long times >24 h. Interestingly, a recent study claimed the economic viability for the production of crosslinked polyesters from glycerol and adipic acid, with costs around 1.7 € kg^−1^ polymer.^[^ ^50^ ^]^ Harsher conditions (150 °C, 2–5 days) have been described for catalyst‐free syntheses of bio‐based polyesters,^[^ ^53^, ^56^ ^]^ with some protocols even at very high temperatures and under high vacuum (220 °C, 0.001 bar).^[^ ^57^ ^]^

Most of these procedures refer to the preparation of materials and polymers (e.g., surfactants) soluble in conventional solvents (water, acetone, chloroform, DMSO) and with modest mechanical properties, while only few examples involving mixtures of monomers and/or more severe processing describe the formation of insoluble thermosets (Table S1).^[^ ^45^ ^]^ These results reflect the achievement of highly crosslinked polymers is a general major issue in the synthesis of bio‐based polyesters using dioic acids and polyols, like AA, glycerol, and sorbitol.

We were prompted to investigate this problem based on the results of our recent article on the poly‐esterification of Kraft lignin with the use of activated AA as a crosslinker.^[^ ^58^ ^]^ In this work, we wish to report that after a deep revision of the protocol originally designed for lignin‐derived polyesters, an original synthesis of sugar alcohols‐based recyclable thermoset polymers was successfully achieved. A three‐steps strategy was carried out where at ≈80 °C, AA was initially activated by acetylation (with either isopropenyl acetate, a nontoxic compound, or acetic anhydride) and converted into a crosslinking mixture (CLM) comprised of adipic acetic mixed polyanhydrides. Thereafter, at 150 °C, the CLM was subjected to a transesterification reaction with both glycerol and sorbitol and mixtures thereof to obtain a representative library of thermoset polyesters (eight examples). These materials not only showed good/improved physicochemical and mechanical properties compared to previously reported adipic acid and glycerol/sorbitol‐based polyesters, but they also proved compatible with a simple treatment of chemical recycling via base‐catalyzed methanolysis.

## 2 Results and Discussion

### 2.1 Synthesis of an Activated Acylating Mixture (CLM)

Several works demonstrated that isopropenyl acetate (iPAc), an enol ester, was effective for the chemical upgrading of a variety of biomass derivatives.^[^ ^59^, ^60^ ^]^ iPAc was a privileged reagent because of its nontoxicity and its peculiar reactivity: when involved in esterification or transesterification reactions, it originates an enol as a coproduct that quickly converts into the corresponding ketone (acetone), thereby making the overall transformation irreversible.^[^ ^61^ ^]^

iPAc was successfully used also in this work to promote the initial activation/conversion of adipic acid into an activated acylating mixture comprised of adipic acetic mixed polyanhydrides. The latter were far more powerful electrophiles than AA, for the subsequent synthesis of crosslinked polyesters with both glycerol and sorbitol. Since the activated acylating mixture was used with this specific purpose (polymer preparation), it was named as CLM in this work. The reaction for the preparation of CLM was the same as described in a recent article by our group,^[^ ^58^ ^]^ but the synthetic protocol was deeply revised here.

A parametric analysis of the process allowed a substantial improvement of the procedure with a reduction of both PMI (process mass intensity) and e‐factor:^[^ ^62^ ^]^ 1) the iPAc excess (with respect to AA) was halved from 4 to 2, 2) the catalyst (H_2_SO_4_) amount was decreased by 100 times, from 0.5 mol% to 0.05 mol%, 3) the amount of the most active sites for crosslinking,^[^ ^58^ ^]^ that is, adipoyl anhydride groups, was tripled from 1.8 to 5.4 mmol g^−1^, and 4) the reaction was scaled up to 500 g without affecting its outcome.

**Figure 3:**
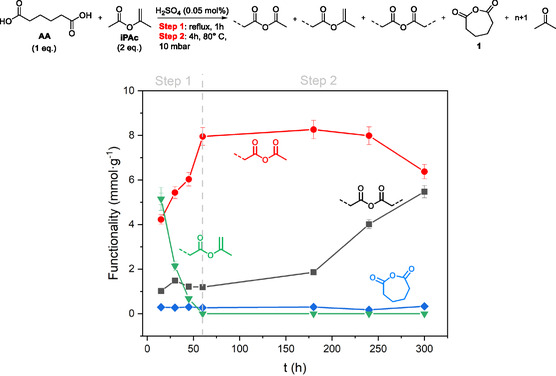
Top: the synthesis of the adipic acid (AA)‐based CLM with isopropenyl acetate (iPAc). Bottom: the trend of the reaction over time. 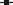 Symmetric anhydrides 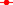 Acetyl anhydrides 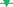 Isopropenyl esters 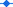 Adipic anhydride (**1**). Determined by ^1^H NMR with dioxane as the internal standard. The decline in isopropenyl ester signals was due to the formation of mixed anhydrides via an acid‐catalyzed transesterification with carboxylic acid groups, accompanied by the release of acetone.

**Figure** **3** (bottom) describes a kinetic profile for the preparation of the CLM mixture according to the conditions set in this work. A two‐step one‐pot synthesis was performed (Figure 3, top). In step 1, a mixture of AA and iPAc (1:2 mol mol^−1^) was set to react at the reflux temperature (97 °C) in presence of H_2_SO_4_ (0.05 mol%) as the catalyst. After 1 h, step 2 started via a vacuum distillation of the residual iPAc and the coproduct acetone. Both these compounds were quantitively recovered at 80 °C and 10 mbar, in 4 h. NMR analyses confirmed the structure of the obtained products (Supporting Information section). The progress of the reaction showed that after the initial formation of oligomers bearing isopropenyl ester groups (green profile, in step 1), these were progressively converted into acetyl anhydrides which in turn, over time, yielded the desired adipoyl anhydrides (red and black profiles, respectively, in step 2). This transformation and the disappearance of the isopropenyl ester functions occurred with the release of acetone. A plausible mechanism is provided in Figure S1, Supporting Information.^[^ ^61^ ^]^ At the end of the second step, the CLM was comprised of adipoyl anhydrides (AdOCOOAd; Ad = adipoyl) and acetyl anhydrides (AcOCOOAd) in amounts of 5.4 and 6.4 mmol g^−1^, respectively. Heating under vacuum was crucial to achieve this result, but no appreciable variations of the CLM composition were noticed even when step 2 was prolonged up to 8 h. Furthermore, a small but detectable amount of adipic anhydride (**1**, ≈0.3 mmol g^−1^) was observed. The molar mass of the CLM was ≈3000 Da, evaluated via ^1^H NMR.

For comparison, the synthesis of CLM was conducted by replacing iPAc with acetic anhydride (Ac_2_O) as a conventional acetylating agent. With respect to iPAc, the use of Ac_2_O released acetic acid (AcOH) as a major by‐product. This compound was readily removed under vacuum distillation not only to prevent its accumulation in the reaction mixture but also to avoid its release into the environment and eliminate any unpleasant odor during the subsequent curing step. Under the conditions of Figure 3 (top), the outcome was similar to that described for iPAc: the CLM obtained from Ac_2_O consisted of adipoyl anhydrides and acetyl anhydrides in amounts of 7.9 and 4.2 mmol g^−1^, respectively. Some (carboxylic) acid functional groups, up to 0.9 mmol g^−1^, were also observed (Figure S2, Supporting Information). This result provided further evidence that iPAc was an alternative to acetic anhydride for the activation of AA investigated in this work. Indeed, compared to acetic anhydride, iPAc was not only a greener, nontoxic, biodegradable, and renewable‐based reagent, but notably, it did not face any regulatory restrictions.^[^ ^61^ ^]^

### 2.2 Activity of the CLM Obtained from iPAc and Ac_2_O

Preliminary control experiments were aimed at comparing the behavior of CLM derived from iPAc and Ac_2_O, referred to as CLM_i_ and CLM_a_, respectively, in the synthesis of bio‐based polyesters. Both CLM_i_ and CLM_a_ were set to react with glycerol and with sorbitol to prepare four polymeric specimens (Supporting Information Section). This study revealed that the nature of the CLM did not affect the resulting materials: characterization analyses by fourier transformed infra red (FT‐IR) spectroscopy, Thermal gravimetric analysis (TGA), and DSC proved that glycerol‐derived polymers were substantially equivalent irrespective of whether the CLM originated from iPAc or Ac_2_O. The same held true for sorbitol‐derived samples.

Results confirmed the potential of iPAc as a greener and safer reagent compared to acetic anhydride that poses health risks, corrosivity issues, and legal restrictions.^[^ ^63^ ^]^ For practical reasons, however, such as the lower cost of Ac_2_O, its larger availability, and the need to design the preparation of polymers on a mid‐large scale (20–500 g for each test), the CLM_a_ was selected for the continuation of this work.

### 2.3 Synthesis of the Glycerol‐/Sorbitol‐Based Polyesters

**Figure 4:**
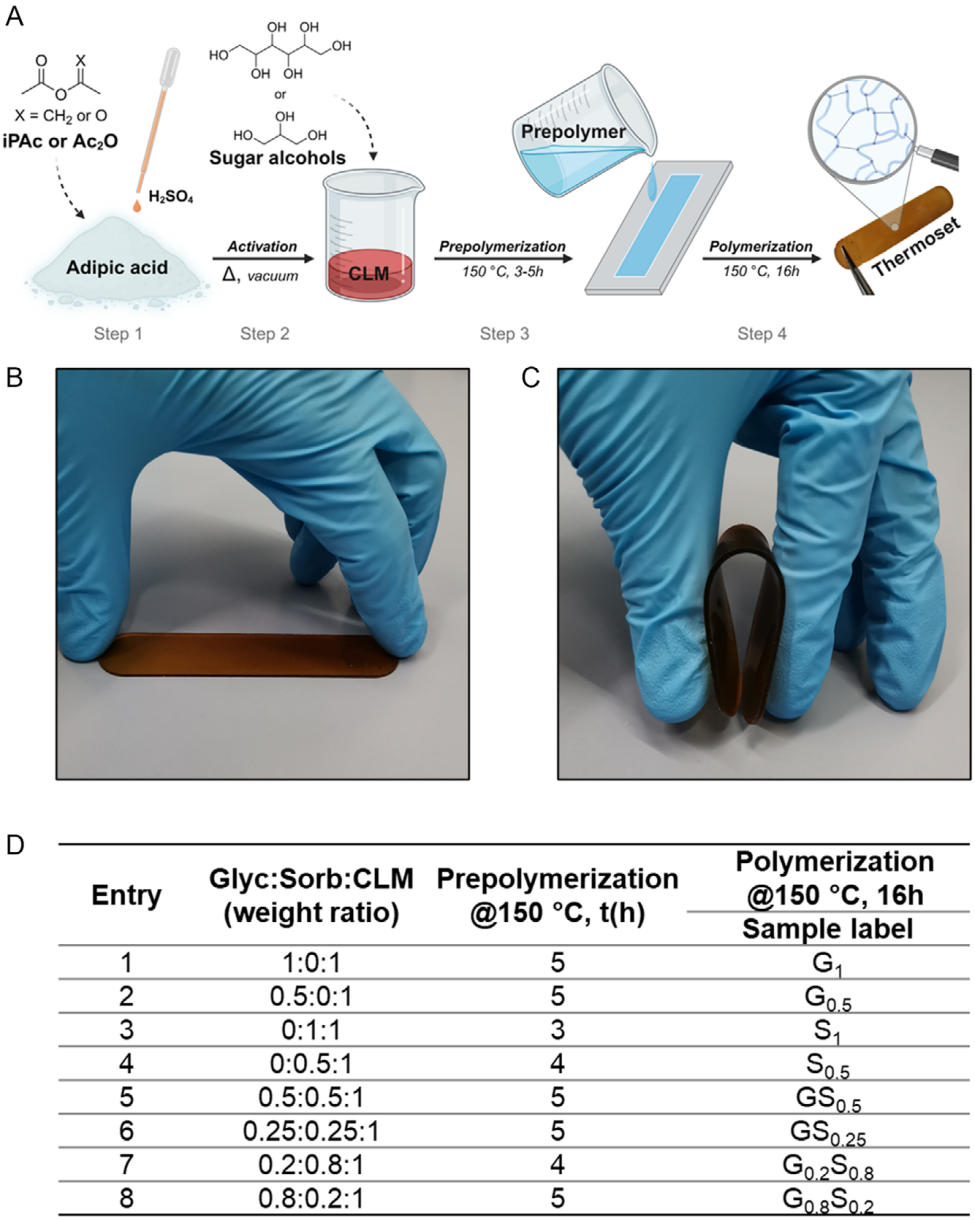
A) Representation of the preparation of glycerol and sorbitol poly‐adipates. B) Flat and C) bent typical polyester sample (S_1_). D) Prepolymerization and polymerization conditions and labelling of the synthesized polyester samples. Glyc:Sorb:CLM = glycerol:sorbitol:CLM weight ratio in the prepolymer synthesis.

Glycerol and sorbitol (sugar alcohols) and their mixtures thereof were set to react with the above described CLM_a_. A representation of the synthetic procedure is shown in **Figure** **4**A, complemented by a Table outlining reaction conditions. Different sugar alcohols:CLM weight ratios were used to obtain eight different samples. In the first part of the polyester preparation (step 3), the transesterification of CLM with sugar‐alcohols originated liquid prepolymers along with volatile by‐products, predominantly acetic acid (AcOH, vide infra).

At 150 °C and ambient pressure, the gelation time required for molding the prepolymers was 3–5 h at 150 °C and atmospheric pressure, depending on the reactant sugar alcohol used and its amount (Figure 4D). The control of prepolymerization conditions was crucial also to minimize the release of bubbles due to volatiles.

The curing was carried out at 150 °C by pouring the hot liquid prepolymers into a silicon mold and let them react/settle for 16 h (step 4). This duration standardized the protocol for the preparation of different samples (Figure 4D), based on: 1) the conditions previously reported by us for the synthesis of lignin‐derived thermoset polyesters^[^ ^58^ ^]^ and 2) control experiments that indicated that a prolonged curing (at least 14 h) was necessary even for sugar alcohols‐based prepolymers to achieve highly crosslinked final materials.

Additional DSC experiments were carried out to further analyze the curing behavior of the investigated polyesters. Mixtures of glycerol: CLM and sorbitol: CLM in a 1:1 wt ratio, were heated from ambient temperature up to 250 °C. The corresponding DSC profiles highlighted a substantial heat flow through the samples, consistent with the onset of crosslinking reactions, at T above 133 °C (Figure S18, Supporting Information), suggesting that 150 °C was an appropriate curing temperature.

**Figure 5:**
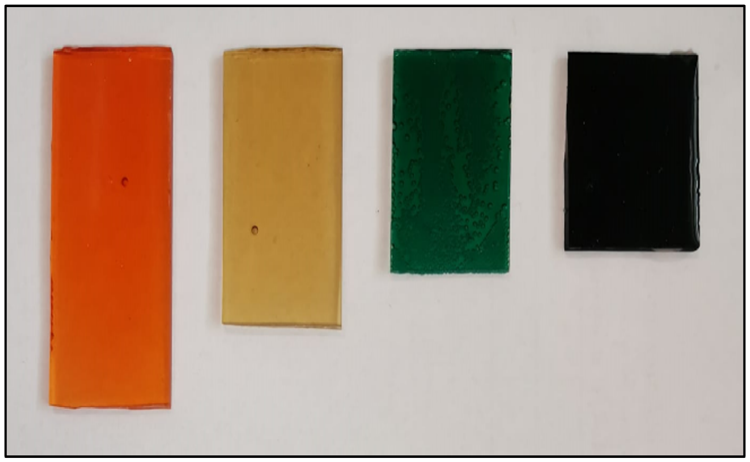
Examples of dyed samples of polyesters obtained by the reaction of glycerol with CLM. Conditions of prepolymerization and curing were those of Figure 4.

Thermoset polyesters were isolated in the form of dry, flexible, and transparent films (≈2 mm mm thickness) of amber color (Figure 4B,C). Both the shape and the color, however, could be easily modified by changing the mold and adding dye to the prepolymer before curing. **Figure** **5** shows orange, pale yellow, green, and dark blue samples that were obtained by using rose bengal, brilliant yellow, brilliant green, and methylene blue as dyes (0.1 wt% with respect to the prepolymer added during step 2), respectively.

**Figure 6:**
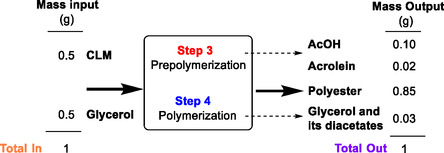
Evaluation of the mass balance for a typical preparation of the polyester G_1_.

The mass balance of the overall polymerization procedure was validated by analyzing the weight loss during steps 3 and 4 of two representative samples, G_1_ and S_1_, that were achieved starting from pure glycerol and sorbitol (Table S3). The formation of volatile compounds in 12 wt% and 11 wt% with respect to the total mass of reactants was observed during the prepolymerization (step 3, Figure 4A). These volatiles included AcOH and acrolein in a 7.4:1 mol:mol ratio for G_1_ and of AcOH alone for S_1_. Acrolein was plausibly due to a side‐dehydration reaction of glycerol, catalyzed by the acidity of the CLM.^[^ ^64^ ^]^ Other volatile mixtures (≈3 wt% with respect to the total mass of reactants in total) also formed during the curing (step 4). Albeit the composition of these fractions was complex, main products were identified as glycerol and its acetates for G_1_ and isosorbide and its diacetate for S_1_, respectively (Tables S4 and S5). **Figure** **6** exemplifies the evaluation of the mass balance for a typical preparation of the polyester G_1_. The gravimetric determination of the amounts of reagents (CLM and glycerol, left) and products (polyester G_1_ and volatiles from both step 3 and 4, right) proved a substantial match between input and output mass and confirmed that the polymer was achieved in a good 85% mass yield. The mass balance was equally validated for the polyester S_1_ (from sorbitol), which was isolated in 86% mass yield.

Shortening the prepolymerization to 30 min resulted in the production of final materials with bubbles of a relatively uniform size (diameter in the range of 0.5–15 and 0.15–0.25 mm from glycerol and sorbitol, respectively) by encapsulating the volatiles in the polymeric matrix, that is, polyester foams (Figure S31, Supporting Information).

### 2.4 Structural Characterization of the Polyesters

**Figure 7:**
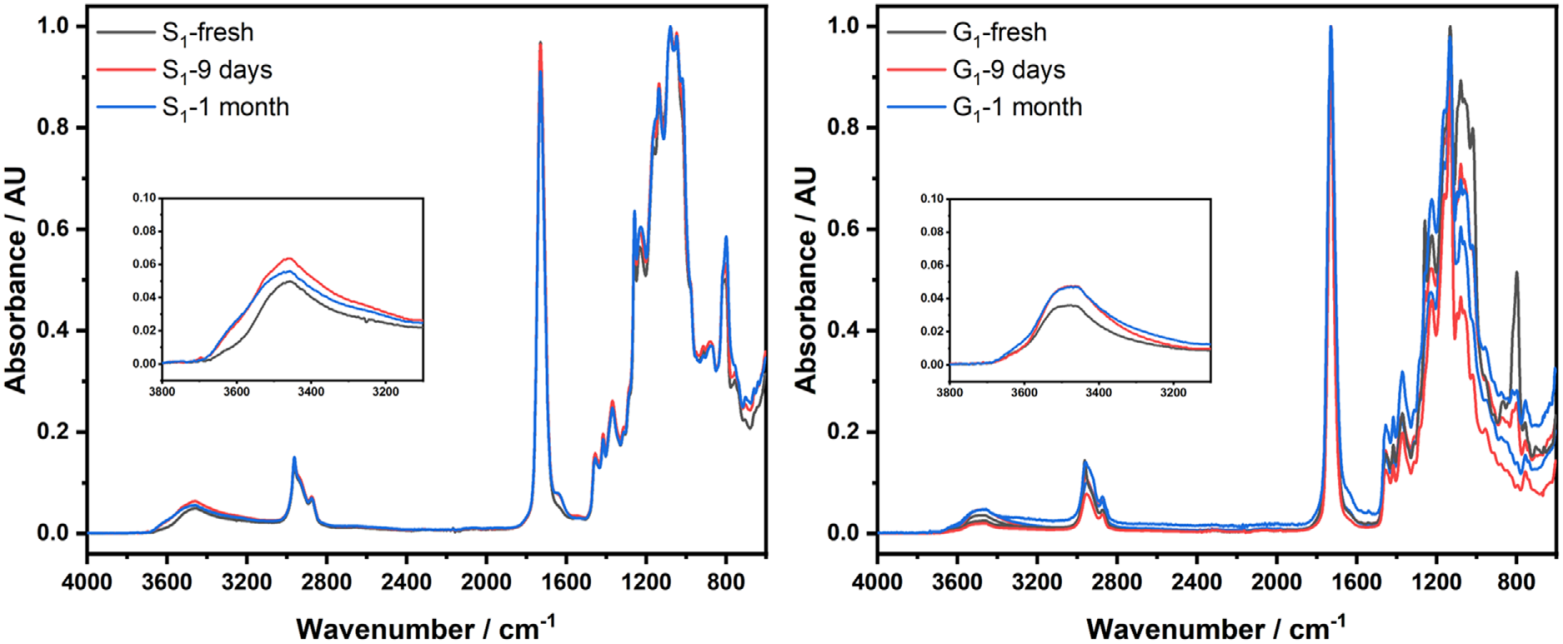
FT‐IR spectra of samples S_1_ (left) and G_1_ (right) over time.

**Figure 8:**
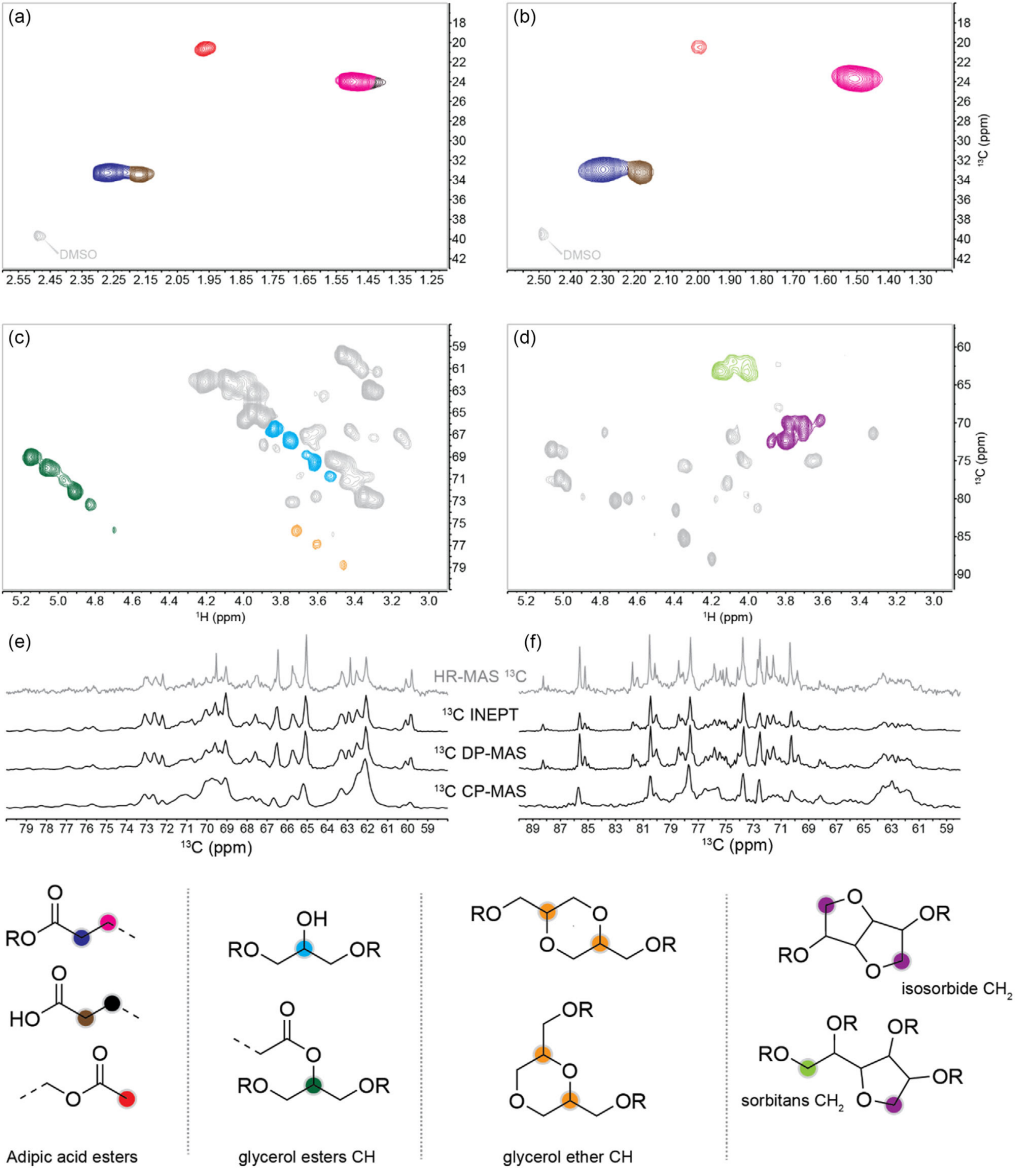
a,b): aliphatic regions of HR‐MAS HSQC NMR spectra of samples G_1_ and S_1_, respectively. c,d): oxygenated aliphatic regions of HR‐MAS HSQC NMR spectra of samples G_1_ and S_1_, respectively. e) Overlay of ^13^C HR‐MAS, INEPT, DP‐MAS, and CP‐MAS NMR spectra. (e) and f): Overlay of 13C HR‐MAS, INEPT, DP‐MAS, and CP‐MAS NMR spectra for G1 nd S1 respectively.

The occurrence of polyesterification was confirmed by solid state and high‐resolution magic angle spinning ^13^C and HSQC NMR, and FT‐IR analyses. As an example, **Figure** **7** and **8** report the characterization data for samples G_1_ and S_1_.

#### 2.4.1 FT‐IR Spectra

Figure 7 shows normalized FT‐IR spectra of G_1_ (left) and S_1_ (right) recorded at three different time intervals (fresh, 9 days, and 1 month of storage at ambient conditions, in air). The substantial overlap of the three spectra for each sample suggested that no significant modification of the chemical structure of the polymers was appreciable over time, particularly indicating a substantial moisture stability of the materials.

The intense signal at 1750 cm^−1^ was typical of the carbonyl stretching of aliphatic esters and the almost complete disappearance of peaks in the range 3000–3600 cm^−1^ indicated that the hydroxyl functionalities considerably converted into esters. The absorbance at around 800 cm^−^^1^ was associated with out‐of‐plane bending vibrations, more specifically to C—O—C bending modes of the ester group or rocking vibrations of –CH_2_– groups in the polymer backbone, especially in regular, repeating aliphatic segments. The absence of any signal at 1850 cm^−1^–the typical symmetric C=O stretching of noncyclic saturated anhydrides–corroborated the full conversion of the reactive functions of CLM during the synthesis of polymers. For comparison, Figure S32, S33, and S34, Supporting Information, show the FT‐IR spectra of CLM, glycerol, and sorbitol, respectively. Similar considerations were drawn from the FT‐IR analysis of the other samples (Figure S35–S40, Supporting Information). It should also be noted here that from a visual inspection, both G_1_ and S_1_ samples showed no appreciable changes even when fully immersed in water for one week.

#### 2.4.2 High‐Resolution Magic Angle Spinning (HR‐MAS) HSQC NMR

The peaks at δ_C_/δ_H_ = 34–32/2.35–2.24 ppm and δ_C_/δ_H_ = 34–32/2.24–2.10 ppm in the aliphatic regions of the HR‐MAS HSQC of G_1_ and S_1_ (Figure 8a,b) suggested the presence of adipic acid esters and carboxylic acid functionalities in both the glycerol‐ and sorbitol‐based materials.^[^ ^46^ ^]^ These assignments, corroborated by HR‐MAS HMBC data (Figure S41 and S42, Supporting Information), allowed us to evaluate the ratio between adipic acid esters and acid functionalities via the deconvolution of the two peaks in the ^1^H spectrum at δ_H_ = 2.35–2.24 ppm and δ_H_ = 2.24–2.10 ppm, respectively (Figure S43 and S44, Supporting Information).

The ratio between ester:acid functionalities was 86:14 mol% and 83:17 mol% for G_1_ and S_1_, respectively. In addition, acetyl esters were identified based on the signal of the CH_3_–COOR moiety at δ_C_/δ_H_ = 22–20/2.0–1.9 ppm.^[^ ^65^ ^]^ The green and light blue clusters in the oxygenated aliphatic region of G_1_ (Figure 8c) at δ_C_/δ_H_ = 77–69/5.2–4.7 ppm and δ_C_/δ_H_ = 73–66/3.9–3.5 ppm, respectively, confirmed the presence of glycerol mono‐/di‐/tri‐esters.^[^ ^46^, ^66^, ^67^, ^68^ ^]^ This piece of evidence (along with FT‐IR) strongly supported the fact the primary mechanism for the formation of G_1_ and S_1_ materials was based on the esterification of activated acylating mixture (CLM) by the hydroxyl groups of the nucleophilic polyols as reaction partners. Other polymerization pathways based on isopropenyl double bonds were ruled out since the CLM obtained from iPAc no longer contained traces of isopropenyl groups (Figure 3).

The orange cluster at δ_C_/δ_H_ = 75–79/3.8–3.4 ppm indicated esters of glycerol ethers.^[^ ^69^ ^]^ These compounds (ethers) plausibly formed via an acid catalyzed reaction of glycerol at high T.^[^ ^70^ ^]^ In the case of the S_1_ sample, the HSQC analysis suggested that sorbitol was fully converted into sorbitans and isosorbide.^[^ ^71^ ^]^ These compounds derived subjected from an acid catalyzed dehydration of sorbitol during the polymer synthesis. Esters of isosorbide and sorbitans were also detected based on the signals at δ_C_/δ_H_ = 75–70/3.6–3.9 ppm and δ_C_/δ_H_ = 65–60/4.2–3.9 ppm, respectively.^[^ ^72^, ^73^ ^]^

#### 2.4.3 Solid‐State ^13^C NMR

The HR‐MAS NMR technique described in the previous paragraph mainly analyzes highly plasticized and dynamic regions and does not give info on highly rigid environments. Thus, solid‐state NMR of the DMSO‐*d*_6_ swollen materials was used as a supplementary characterization technique to offer a more comprehensive understanding of the carbon environments. Three solid‐state NMR experiments were performed for the G_1_ and S_1_: 1) Cross‐polarization magic angle spinning (CP‐MAS), which emphasized more rigid carbon environments; 2) direct polarization magic angle spinning (DP‐MAS), which displayed all carbons regardless of their mobility, though not quantitatively; and 3) insensitive nuclei enhancement by polarization transfer (INEPT), which selectively detected highly dynamic carbons with covalently bound protons. These ssNMR spectra were compared to the corresponding HR‐MAS ^13^C spectra (Figure 8e,f and Figure S45–S52, Supporting Information). The ^13^C ssNMR data showed three main regions: clear peaks of CO acid and ester groups were present in the range 170–180 ppm, peaks of aliphatic chain of esterified and nonesterified AA at 20–40 ppm, and the characteristic signals of esterified glycerol and sorbitol in G_1_ and S_1_ were found at δ_C_ = 60–80 ppm and δ_C_ = 60–90 ppm, respectively. Similar peaks in the ^13^C ssNMR spectra of dry samples were spotted for all the other samples (Figure S53–60). Across all methods—CP‐MAS, DP‐MAS, INEPT, and HR‐MAS—the ^13^C spectra of the G_1_ sample showed strikingly similar features and spectral intensities in the oxygenated aliphatic region (δ_C_ = 90–60 ppm). This uniformity across ssNMR techniques, which emphasized carbons with differing dynamics, suggested that the G_1_ polymer was homogeneously swollen by the plasticizing solvent, retaining a balance between mobility and rigidity. For S_1_, a similar behavior of near‐homogeneous polymer swelling was observed, however select regions were only visible under DP/INEPT but not by CP. For example, sharp ^13^C signal clusters near 88, 85, and 82 ppm seen in the HR‐MAS, DP, and INEPT spectra were not observed in the CP spectrum. This evidence was interpreted considering a more rigid and dynamically hindered cross‐linked core with more dynamic terminal end groups, consistent with the conceptual model of hyperbranched polymers in Figure 2.

### 2.5 The Properties of the Polyesters

#### 2.5.1 Thermal Properties and Stability

TGA allowed us to determine *T*_d5%_ and *T*_d50%_, the temperatures at which 5% and 50% of the initial mass of each sample were lost, respectively, during heating (see SI). Samples exhibited relatively high *T*_d5%_ values, above 260 °C, that were higher for materials as G_1_‐G_0.5_ and S_1_‐S_0.5_ (308–339 °C; entries 1–4) prepared from pure monomers than for GS‐polyesters (262–306 °C; entries 5–8) achieved from mixtures of glycerol and sorbitol. The *T*_d50%_ values, instead, oscillated within a narrower range of 409–424 °C for all the eight samples. These data confirmed a high thermal stability in all cases and were consistent with the occurrence of a substantial crosslinking across the materials, in agreement with trends reported for other bio‐based polyesters.^[^ ^74^, ^75^ ^]^

The glass transition temperature (*T*_g_), as determined by DSC, was remarkably affected by both the type of sugar alcohol and its proportion relative to the CLM (Figure S63, Supporting Information). The *T*_g_ values ranged from −4.6 to 41.1 °C, with the highest and the lowest value recorded for a polymer prepared from pure sorbitol and a polymer obtained from a glycerol‐rich mixture (glycerol: sorbitol = 4:1 weight ratio), respectively. These two extremes of *T*_*g*_ were referred to samples S_1_ and G_0.8_S_0.2_ prepared from sugar alcohol(s) and CLM in a 1:1 weight ratio (entries 3 and 8). Moreover, it was noticed that: 1) polymers G_1_ and G_0.5_ containing glycerol (entries 1‐2) exhibited a *T*_*g*_ lower than that of the equivalent sorbitol‐based materials (S_1_ and S_0.5_, entries 3‐4) and 2) when mixtures of sugar alcohols were used, a higher proportion of glycerol relative to sorbitol led to lower *T*_*g*_ values (entries 5‐8). Consistently with a different degree of crosslinking, the observed changes of *T*_g_ indicated that materials with different elastic properties and brittleness were synthesized by adjusting the CLM:sugar alcohol(s) ratio. Previous studies established that the two primary hydroxyl groups of both sugar alcohols were kinetically equivalent, and they acted as the sole reactive sites in polymerization reactions with no other transformations occurring.^[^ ^27^, ^76^ ^]^ However, under the conditions explored in this work, the NMR characterization revealed that most of glycerol underwent the transesterification with CLM by preserving its original structure, while sorbitol did not as, it was subjected to dehydration and largely converted into isosorbide and sorbitans. Such molecules, particularly isosorbide, have been reported able to induce a significant increase of the *T*_*g*_ for a variety of polymers including polyurethanes, polyesters and polyacrylates, as it was observed in **Table** **1**.^[^ ^77^, ^78^, ^79^ ^]^ As a final note, *T*_*g*_ gradually increased by increasing the cross‐linking density of the material in R1 and R2 (entries 9‐10), consistent with our findings.

**Table 1:** The thermal and tensile properties, and the gel fraction of polyesters examined in this work.

Entry	Sample	*T*_d5%_ [°C]	*T*_d50%_ [°C]	*T*_g_ [°C]a)	Gel content [wt%]b)	*σ* c) [MPa]	*ε* d) [%]	*E* e) [MPa]	Ref.
1	G_1_	339	424	0.4	99.6	1.8 ± 0.5	11 ± 2	16 ± 2	This work
2	G_0.5_	322	424	−0.5	94.7	1.4 ± 0.2	4 ± 1	35 ± 1	This work
3	S_1_	308	412	41.1	99.4	18 ± 3	60 ± 13	50 ± 1	This work
4	S_0.5_	337	421	4.4	97.6	3.2 ± 0.3	20 ± 2	17 ± 1	This work
5	GS_0.5_	306	421	8.4	97.6	2.6 ± 0.7	16 ± 5	18 ± 2	This work
6	GS_0.25_	262	409	12.3	99.2	3.4 ± 0.3	7.5 ± 0.6	41 ± 3	This work
7	G_0.2_S_0.8_	263	416	4.4	99.6	5.8 ± 0.2	24.7 ± 3	27 ± 1	This work
8	G_0.8_S_0.2_	291	414	−4.6	97.7	2.7 ± 0.3	16 ± 2	18 ± 1	This work
9	R1	n.a.f)	n.a.f)	−48 to −38	88.7–95.5	0.36–2.42	542–1728	0.08–0.86	[57]
10	R2	n.a.f)	n.a.f)	−30 to −3	92.2–99.6	0.16–0.68	28.5–193.8	0.07–5.0	[45]

a) Evaluated by DSC.

b) Evaluated in acetone.

c) Tensile strength.

d) Tensile strain.

e) Young's modulus.

f) n.a. = not available.

The gel content was above 95% for all the samples. Albeit no clear trends were spotted either varying the type/amount of sugar alcohol or the CLM, the gel content was comparable, but often higher, than that of previously reported insoluble polyesters belonging to the R1 and R2 families,^[^ ^45^, ^54^, ^57^ ^]^ thereby further corroborating the fact that the synthesis designed in this work was effective to achieve highly crosslinked materials.

#### 2.5.2 Mechanical Properties

Mechanical properties were variable among the eight investigated polyesters, but in general, the values of stretchability, length of deformation and stiffness (tensile stress, tensile strain, and Young's modulus: *σ*, *ε*, and *E*, respectively) were superior for materials prepared from sorbitol than for those obtained from glycerol or from glycerol‐rich mixtures as crosslinkers (compare entries 1‐2 and 3‐4, and 7 and 8).

This behavior was especially exemplified by the S_1_ thermoset synthesized from a 1:1 mixture of pure sorbitol and CLM (entry 3). This sample provided an outstanding stress resistance of ≈18 MPa which was between one and two orders of magnitude higher than that of the other specimens (entries 1‐2, and 4–8) including polyesters R1 and R2 (entries 9‐10). The latter, however, showed higher and lower *ε* and E values, respectively, compared to S_1_ and, more in general, to all other polymers of Table 1. This difference was ascribed to the protocol for the preparation of both R1 and R2, particularly the short polymerization time (6 h @180–225 °C, for R1) and the plasticizing effect of 1,4‐butanediol and ethylene glycol for R1 and R2, respectively. Moreover, S_1_ also possessed the highest tensile strain (60%) and Young's modulus (50 MPa) among the eight investigated polymers of Table 1. The replacement of sorbitol with glycerol led to a material (G_1_) with values of *σ*, *ε*, and *E* that were 10‐, 6‐, and 3‐fold lower, respectively, than those of S_1_ (entry 1). Mechanical tests also highlighted that decreasing the sugar alcohol(s):CLM weight ratio was detrimental for both the tensile strength and strain (compare entries 1‐2 and 3‐4).

Overall, results proved consistent with the synthesis of polyesters with a highly variable crosslinking degree, where the presence of sorbitol‐derived sorbitans/isosorbide tended to originate more reticulated materials with a higher resistance to stress and strain, while glycerol acted as a plasticizer, decreasing tensile strength. Noteworthy, an influence on the mechanical properties of the sorbitol‐based thermosets by the sorbitans/isosorbide in addition to sorbitol should be considered. The stiffness (E) of the polymers did not seem to follow any clear trend among the samples. Apparently, decreasing the amount of sorbitol (from S_1_ to G_0.2_S_0.8,_ S_0.5,_ and GS_0.5_: entries 3, 7, 4 and 5) induced a drop in the elastic modulus in line with the achievement of progressively less reticulated materials. Such tendency, however, was contrasted by the behavior of samples G_0.5_ and GS_0.25_: albeit the content of glycerol and sorbitol in these materials was lower compared to G_1_ and S_0.5_, respectively, they showed surprisingly high E values (compare entries 1 and 2, and 3 and 6).

Additional DMA (dynamic mechanical analysis) tests carried out on samples G_1_ and S_1_, offered values of *T*_*g*_ in good agreement with those obtained by DSC analysis. Moreover, below and above *T*_*g*_, DMA proved that the storage modulus (E′) was higher than the loss modulus (E″) for both samples, thereby indicating that the elastic component was predominant over the viscous one (Figure S64 and S65, Supporting Information).

### 2.6 The Chemical Recycling of the Bio‐Based Polyesters

The chemical recycling/upcycling of polymers is conceptually different from the conventional “melt‐and‐reshape” approach, as, in principle, it targets a virtually endless recycle and/or upgrade of the monomers without loss of value.^[^ ^80^, ^81^ ^]^ This is even truer for thermosets, which, by definition, are not suitable for a melt and reshape process. In this perspective, the chemical depolymerization of G_1_ and S_1_ samples, chosen as models for glycerol‐ and sorbitol‐based thermosets, and the subsequent polymerization of the recovered monomers were investigated.

#### 2.6.1 The Depolymerization of G_1_ and S_1_

**Figure 9:**
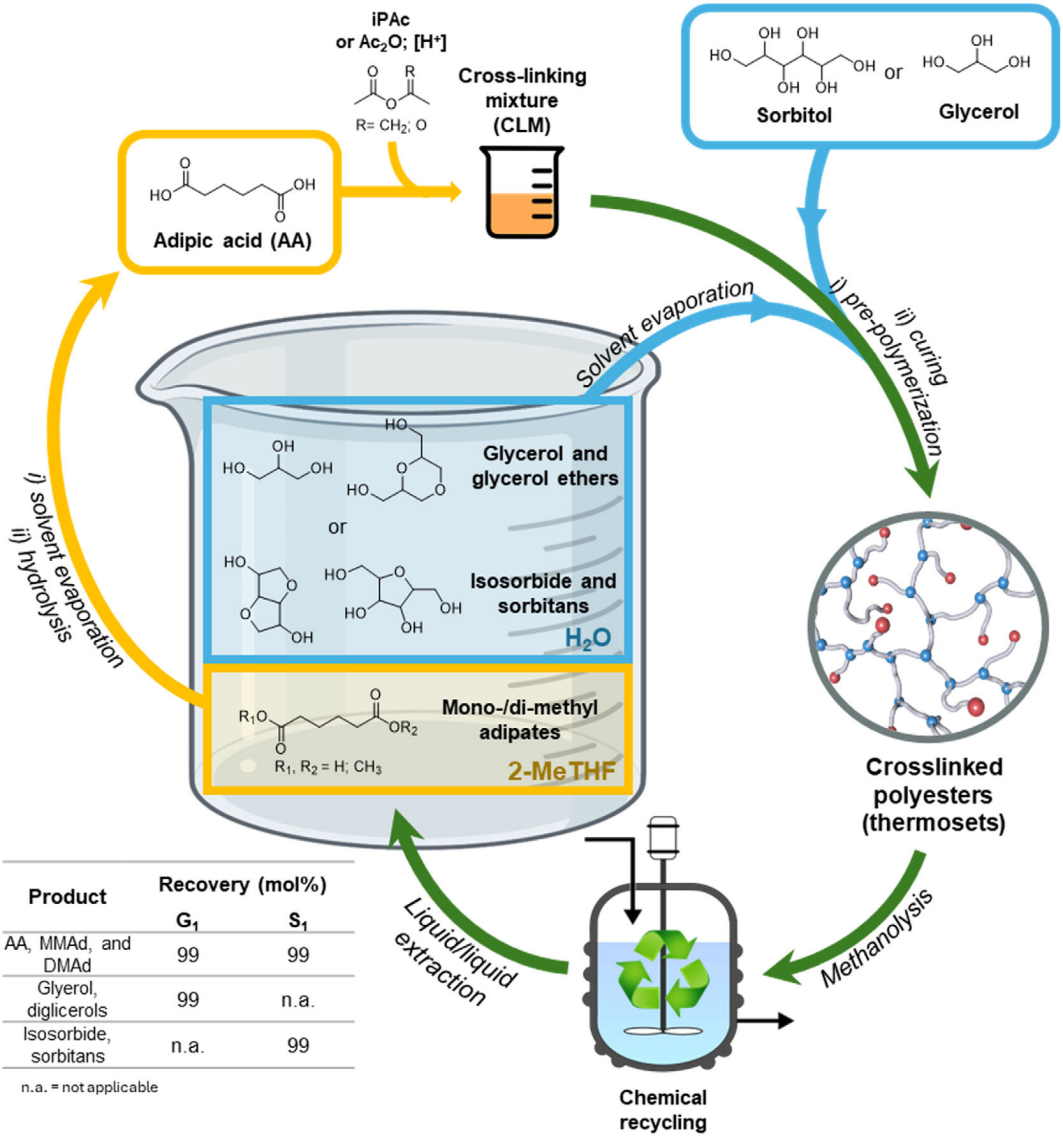
Synthesis and recycle of the polyesters.

The depolymerization was carried out after a screening of different reactions, which led us to identify a base‐mediated methanolysis as an effective strategy, active under mild conditions. Polyesters G_1_ and S_1_ were reacted at 50 °C and atmospheric pressure with a 1 wt% NaOH methanol solution for 1 h, providing homogenous solutions which were subjected to a liquid/liquid extraction (H_2_O/2‐MeTHF, 1:1 v/v; see Supporting Information). The content of the two phases, after removal of both water and the organic solvent, was analyzed by NMR and GC/MS (see Supporting Information). The overall procedure is depicted in **Figure** **9**.

In the case of G_1_, glycerol and diglycerols (linear and cyclic: 3,3′‐oxybis(propane‐1,2‐diol), (1,4‐dioxane‐2,6‐diyl)dimethanol), and (1,4‐dioxane‐2,5‐diyl)dimethanol) were detected as aqueous extracts and quantified in 4:1 molar ratio respectively; the overall amount of these compounds corresponded to 99 mol% of starting (reactant) glycerol. The components of the organic fraction were adipic acid and its mono‐ and di‐methyl esters (AA+MMAd:DMAd = 1:6.6 molar ratio). The weight‐based (mass) recovery of these compounds was 117 wt% (>99 mol%) because of the incorporation of methyl groups in the ester derivatives. In the case of S_1_, the aqueous extracts were isosorbide and a complex mixture of C6 polyols that included, among others, 1,4‐anhydrosorbitol, and 1,5‐anhydrosorbitol (see Supporting Information). These products summed up to ≈79 wt% (>99 mol%) of the reactant sorbitol, a result which was consistent with the loss of water due to the dehydration of sorbitol itself during the synthesis of S_1_. Sorbitol was not detected as a depolymerization product, in line with the results of HSQC HR‐MAS spectra on the characterization of the S_1_ polyester (vide infra).

The organic extracts were methyl adipates MMAd and DMAd (>99% pure by NMR) isolated in a 113 wt% yield compared to the reactant adipic acid.

#### 2.6.2 The Synthesis of Polymers from Recycled Monomers

Mono‐ and di‐methyl esters derived from the depolymerization of G_1_ and S_1_ were converted into adipic acid (r‐AA) in quantitative yield via an acid catalyzed hydrolysis procedure (H_2_SO_4_ aq. 1%v/v; t = 5 h; T = 150 °C; MeOH aq. 10 v%; see Supporting Information). Thereafter, the multi‐step procedure of Figure 4A comprised of the synthesis of recycled CLM (r‐CLM), prepolymerization and curing, was followed by the recycling of the recovered components. The transesterification of r‐CLM with the mixtures of glycerol+diglycerols and isosorbide+sorbitans provided two polyesters which were labelled as r‐G_1_ and r‐S_1_, respectively. These materials were characterized by FT‐IR, DSC, TGA, tensile tests, and gel content (Table S8).

A substantial overlap was noticed among normalized FT‐IR spectra of original and recycled polymers, thereby indicating full structural similarities between G_1_ and r‐G_1_, and S_1_ and r‐S_1,_ respectively (Figure S94 and S95, Supporting Information). The presence of an intense signal at 1750 cm^−1^ typical of the carbonyl stretching of aliphatic esters together with the almost complete absence of peaks in the range 3000–3600 cm^−1^ in all the spectra indicated that transesterification of OH groups occurred. Moreover, the similar intensities (two‐by‐two: G_1_ vs. r‐G_1_, and S_1_ vs. r‐S_1_) of the latter peaks in the normalized spectra suggested that the extent of transesterification was comparable in the recycled glycerol‐ and sorbitol‐based polyesters.

DSC and TGA analyses corroborated further similarities of original and recycled polyesters. From DSC profiles, *T*_*g*_ values of 0.4 and 0.2 °C and of 41.1 and 39.8 °C were determined for G_1_ and r‐G_1_, and for S_1_ and r‐S_1_, respectively (Figure S96 and S97, Supporting Information). From TGA measures, significant was the comparison of T_d50%_ that indicated close values for corresponding samples, particularly of 422 °C versus 382 °C and of 411 °C versus 423 °C for G_1_ and r‐G_1_, and S_1_ and r‐S_1_, respectively (Figure S98, S99, S100, and S102, Supporting Information).

However, the mechanical properties of r‐G_1_ and r‐S_1_ were different than the fresh materials. The tensile strength, the elongation at break, and the Young's modulus of r‐G_1_ and r‐S_1_ were 0.7 ± 0.1 and 3.4 ± 0.7 MPa, 23 ± 2 and 200 ± 30%, and 4.4 ± 0.3 and 5 ± 4 MPa, respectively (Table S8 and Figure S116–S117, Supporting Information), thereby indicating that the recycled materials bore a lower (maximum) load to fracture and were more flexible and stretchable with respect to the virgin samples. Moreover, the gel content of r‐G_1_ and r‐S_1_ was 94.7% and 93.8%, respectively, lower than that of G_1_ (99.6%) and S_1_ (99.4%). These results were consistent to a lower degree of crosslinking of recycled polyesters compared to the parent polymers. This behavior was ascribed to the occurrence of dehydration reactions during the depolymerization and/or the repolymerization treatments, which brought about a partial loss of OH functionalities in the recovered monomers.

Overall, this investigation proved that the G_1_ and S_1_ thermosets could be chemically recycled into materials with structure and thermal properties comparable to those of the parent materials but different mechanical properties that could make the recycled polymers suited to other applications than the virgin polyesters.

## 3 Conclusion

The demand for bio‐based and recyclable thermosets is expected to see a significant increase in the coming years, which means that there is an urgent need to design new synthetic concepts aimed to improve both the sustainability of the preparation protocols and the properties of these materials. On this premise, the present work has been focused on bio‐based polyesters achieved by the reaction of adipic acid with either glycerol or sorbitol and their mixtures thereof. This process is novel in that the activation of adipic acid with iPAc (or Ac_2_O) reported here is a new key step offering genuine advantages over any existing literature benchmark. To summarize the most relevant ones: 1) the perspective of using nontoxic acetylating agents as isopropenyl acetate to mediate the activation of adipic acid; 2) the preparation of highly crosslinked thermosets with tunable mechanical properties, which are otherwise hard to obtain and, in fact, are rather unconventional in this area and 3) the chemical recycling of the polyesters–very important in the case of thermosets–through depolymerization, recovery of monomeric substrates and subsequent repolymerization. Notably, mild conditions (50 °C, 1 atm) identified for the depolymerization are compatible with an industrially favorable process design perspective. Recycled polyesters show with a composition comparable to the parent polymers but possess different mechanical properties that may even broaden the range of applications of the investigated materials.

Potential applications for the synthesized polyesters are coatings, seals, gummy materials of various types, glues, packaging, foams, and composite materials as, for example, carbon/glass fibers or wood laminates.

## Conflict of Interest

Davide Rigo, Maurizio Selva, Alvise Perosa and Matteo Lorenzon have filed a patent application on this concept (patent number: IT102025000006018).

## Supporting information

Supplementary Material

## Data Availability

The data that support the findings of this study are available in the supplementary material of this article.

## References

[1] R. Chinthapalli , P. Skoczinski , M. Carus , W. Baltus , D. De Guzman , H. Käb , A. Raschka , J. Ravenstijn , Ind. Biotechnol. 2019, 15, 237.

[2] S. Walker , R. Rothman , J. Clean. Prod. 2020, 261, 121158.

[3] Y. Zhu , C. Romain , C. K. Williams , Nature 2016, 540, 354. 27974763 10.1038/nature21001

[4] L. Ritzen , B. Sprecher , C. Bakker , R. Balkenende , Resour. Conserv. Recycl. 2023, 199, 107268.

[5] P. A. Wilbon , F. Chu , C. Tang , Macromol. Rapid. Commun. 2013, 34, 8. 23065943 10.1002/marc.201200513

[6] F. Della Monica , A. W. Kleij , Polym. Chem. 2020, 11, 5109.

[7] F. Seniha Güner , Y. Yaǧci , A. Tuncer Erciyes , Prog. Polym. Sci. 2006, 31, 633.

[8] M. Galià , L. M. de Espinosa , J. C. Ronda , G. Lligadas , V. Cádiz , Eur. J. Lipid Sci. Technol. 2010, 112, 87.

[9] A. Duval , M. Lawoko , React. Funct. Polym. 2014, 85, 78.

[10] A. Grossman , V. Wilfred , Curr. Opin. Biotechnol. 2019, 56, 112. 30458357 10.1016/j.copbio.2018.10.009

[11] M. Zaborowska , K. Bernat , Waste Manage. Res. 2023, 41, 68. 10.1177/0734242X221105432 PMC9925894 35765777

[12] J. M. J. Fréchet , C. J. Hawker , I. Gitsov , J. W. Leon , J. Macromol. Sci. ‐ Pure Appl. Chem. 1996, 33, 1399.

[13] A. K. Gayen , R. Singla , S. Ramakrishnan , Chem. Commun. 2024, 60, 1534. 10.1039/d3cc05506j 38252017

[14] Q. Li , X. Shu , P. Jia , Y. Zhou , Polymers 2020, 12, 913. 32326431 10.3390/polym12040913 PMC7240514

[15] Y. H. Kim , O. W. Webster , Macromolecules 1992, 25, 5561.

[16] A. Sunder , J. Heinemann , H. Frey , Chem. ‐ Eur. J. 2000, 6, 2499. 10961393 10.1002/1521-3765(20000717)6:14<2499::aid-chem2499>3.0.co;2-m

[17] P. J. Flory , J. Am. Chem. Soc. 1952, 74, 2718.

[18] R. Hobzová , J. Peter , P. Sysel , Chem. Listy 2008, 102, 906.

[19] Y. H. Kim , J. Polym. Sci. A Polym. Chem. 1998, 36, 1685.

[20] B. Voit , J. Polym. Sci. A Polym. Chem. 2000, 38, 2505.

[21] K. Inoue , Prog. Polym. Sci. 2000, 25, 453.

[22] Y. H. Kim , O. W. Webster , T. D. Getman , C. B. Knobler , J. Am. Chem. Soc. 1990, 112, 4592.

[23] K. L. Wooley , C. J. Hawker , R. Lee , J. M. J. Fréchet , Polym. J. 1994, 26, 187.

[24] R. Spindler , J. M. J. Fréchet , Macromolecules 1993, 26, 4809.

[25] A. Kumar , S. Ramakrishnan , J. Polym. Sci. A Polym. Chem. 1996, 34, 839.

[26] Q. Zhang , M. Song , Y. Xu , W. Wang , Z. Wang , L. Zhang , Prog. Polym. Sci. 2021, 120, 101430.

[27] H. Fu , A. S. Kulshrestha , W. Gao , R. A. Gross , M. Baiardo , M. Scandola , Macromolecules 2003, 36, 9804.

[28] O. Valerio , M. Misra , A. K. Mohanty , ACS Sustainable Chem. Eng. 2018, 6, 5681.

[29] A. S. Kulshrestha , W. Gao , R. A. Gross , Macromolecules 2005, 38, 3193.

[30] E. Malmström , M. Johansson , A. Hult , Macromolecules 1995, 28, 1698.

[31] W. Thielemans , R. P. Wool , Biomacromolecules 2005, 6, 1895. 16004426 10.1021/bm0500345

[32] E. Skoog , J. H. Shin , V. Saez‐Jimenez , V. Mapelli , L. Olsson , Biotechnol. Adv. 2018, 36, 2248. 30389426 10.1016/j.biotechadv.2018.10.012

[33] M. R. Monteiro , C. L. Kugelmeier , R. S. Pinheiro , M. Otávio , S. César , Renewable Sustainable Energy Rev. 2018, 88, 109.

[34] B. Kusserow , S. Schimpf , P. Claus , Adv. Synth. Catal. 2003, 345, 289.

[35] D. Polidoro , G. Stamilla , M. Feltracco , A. Gambaro , A. Perosa , M. Selva , Green Chem. 2023, 25, 6677.

[36] S. He , T. S. Kramer , D. S. Santosa , A. Heeres , H. J. Heeres , Green Chem. 2022, 24, 941. 35177952 10.1039/d1gc03531b PMC8785961

[37] J. R. Ochoa‐Gómez , T. Roncal , Production Of Platform Chemicals From Sustainable Resources. Biofuels And Biorefineries (Eds: Z. Fang , R. Smith Jr. , X. Qi ), Springer, Singapore 2017.

[38] D. R. Vardon , A. Franden , C. W. Johnson , E. M. Karp , M. T. Guarnieri , M. J. Salm , J. Strathmann , G. T. Beckham , Energy Environ. Sci. 2015, 8, 617.

[39] H. Zhang , X. Li , X. Su , E. L. Ang , Y. Zhang , H. Zhao , ChemCatChem 2016, 8, 1500.

[40] M. A. Carnahan , M. W. Grinstaff , Macromolecules 2006, 39, 609.

[41] F. Zeng , X. Yang , D. Li , L. Dai , X. Zhang , Y. Lv , Z. Wei , J. Appl. Polym. Sci. 2020, 137, 48574.

[42] K. Shukla , J. Polym. Res. 2022, 29, 373.

[43] V. Taresco , R. G. Creasey , J. Kennon , G. Mantovani , C. Alexander , J. C. Burley , M. C. Garnett , Polymer 2016, 89, 41. 10.1002/pola.28215 PMC5516180 28781423

[44] M. De Meireles , D. Hansen , P. Fiúza , Mater. Res. 2007, 10, 335.

[45] L. Navarro , N. Ceaglio , I. Rintoul , Polym. J. 2017, 49, 625. 10.1016/j.xphs.2017.05.012 28535975

[46] T. Zhang , B. A. Howell , A. Dumitrascu , S. J. Martin , P. B. Smith , Polymer 2014, 55, 5065.

[47] L. E. Iglesias , Y. Fukuyama , H. Nonami , R. Erra‐balsells , Biotechnol. Tech. 1999, 13, 923.

[48] B. A. Howell , S. T. Lazar , Ind Eng Chem Res 2019, 58, 17227.

[49] R. H. Kienle , F. E. Petke , J. Am. Chem. Soc. 1940, 62, 1053.

[50] L. Bueno , C. Toro , M. Martín , Chem. Eng. Res. Des. 2015, 93, 432.

[51] L. Gustini , B. A. J. Noordover , C. Gehrels , C. Dietz , C. E. Koning , Eur. Polym. J. 2015, 67, 459.

[52] A. Anand , R. D. Kulkarni , K. Patil , V. V. Gite , RSC Adv. 2016, 6, 9843.

[53] V. Kavimani , V. Jaisankar , J. Phys. Sci. Appl 2014, 4, 507.

[54] L. Gustini , C. Lavilla , W. W. T. J. Janssen , A. Martínez De Ilarduya , S. Muñoz‐Guerra , C. E. Koning , ChemSusChem 2016, 9, 2250. 27406029 10.1002/cssc.201600626

[55] A. Kumar , A. S. Kulshrestha , W. Gao , R. A. Gross , Macromolecules 2003, 36, 8219.

[56] A. V. Kavimani , B. Viswanathan Jaisankar , IJACS 2015, 3, 192.

[57] W. Q. Yuan , G. L. Liu , C. Huang , Y. D. Li , J. B. Zeng , Macromolecules 2020, 53, 9847.

[58] D. Di Francesco , D. Rigo , K. Reddy Baddigam , A. P. Mathew , N. Hedin , M. Selva , J. S. M. Samec , ChemSusChem 2022, 15, e202200326. 35312238 10.1002/cssc.202200326 PMC9321611

[59] G. Fiorani , A. Perosa , M. Selva , Green Chem. 2023, 25, 4878.

[60] D. Rigo , D. Polidoro , L. Marcuzzo , A. Perosa , M. Selva , ACS Sustainable Chem. Eng. 2023, 11, 12602.

[61] D. Rigo , A. F. Masters , T. Maschmeyer , M. Selva , G. Fiorani , Chem. ‐ Eur. J. 2022, 28, e202200431. 35385201 10.1002/chem.202200431

[62] R. A. Sheldon , ACS Sustainable Chem. Eng. 2018, 6, 32.

[63] R. Calmanti , M. Galvan , E. Amadio , A. Perosa , M. Selva , ACS Sustainable Chem. Eng. 2018, 6, 3964.

[64] B. Katryniok , S. Paul , M. Capron , F. Dumeignil , ChemSusChem 2009, 2, 719. 19693786 10.1002/cssc.200900134

[65] N. R. Babij , E. O. McCusker , G. T. Whiteker , B. Canturk , N. Choy , L. C. Creemer , C. V. D. Amicis , N. M. Hewlett , P. L. Johnson , J. A. Knobelsdorf , F. Li , B. A. Lorsbach , B. M. Nugent , S. J. Ryan , M. R. Smith , Q. Yang , Org. Process. Res. Dev. 2016, 20, 661.

[66] E. Alexandri , R. Ahmed , H. Siddiqui , M. I. Choudhary , C. G. Tsiafoulis , I. P. Gerothanassis , Molecules 2017, 22, 1663. 28981459 10.3390/molecules22101663 PMC6151582

[67] E. Hatzakis , A. Agiomyrgianaki , S. Kostidis , P. Dais , JAOCS 2011, 88, 1695.

[68] P. Dais , M. Misiak , E. Hatzakis , Anal. Methods 2015, 7, 5226.

[69] G. N. M. Boussambe , R. Valentin , Z. Mouloungui , JAOCS 2015, 92, 1567.

[70] Z. Gholami , A. Z. Abdullah , K. T. Lee , Appl. Catal. A Gen. 2014, 479, 76.

[71] J. M. Fraile , C. J. Saavedra , ChemistrySelect 2017, 2, 1013.

[72] J. A. Bennek , G. R. Gray , J. Org. Chem. 1987, 52, 892.

[73] P. Che , H. Ma , X. Nie , W. Yu , J. Xu , Green Chem. 2022, 24, 7545.

[74] S. P. Arnaud , L. Wu , M. A. Wong Chang , J. W. Comerford , T. J. Farmer , M. Schmid , F. Chang , Z. Li , M. Mascal , Faraday Discuss 2017, 202, 61. 28671209 10.1039/c7fd00057j

[75] I. Bîtcan , A. Pellis , A. Petrovici , D. M. Dreavă , I. Păuşescu , F. Peter , L. Nagy , S. Kéki , L. Gardossi , A. Todea , Sustainable Chem. Pharm. 2023, 35, 101229.

[76] L. Nagy , B. Vadkerti , C. Lakatos , P. P. Fehér , M. Zsuga , S. Kéki , Int. J. Mol. Sci. 2021, 22, 4059. 33920018 10.3390/ijms22084059 PMC8071031

[77] S. W. Lee , J. H. Back , G. S. Shim , S. W. Jang , H. J. Kim , Int. J. Adhes. Adhes. 2020, 98, 102503.

[78] O. Goerz , H. Ritter , Polym. Int. 2013, 62, 709.

[79] S. Oprea , V. O. Potolinca , V. Oprea , Eur. Polym. J. 2016, 83, 161.

[80] S. R. Nicholson , J. E. Rorrer , A. Singh , M. O. Konev , N. A. Rorrer , A. C. Carpenter , A. J. Jacobsen , Y. Román‐Leshkov , G. T. Beckham , Ann. Rev. Chem. Biomol. Eng. 2022, 13, 301. 35320697 10.1146/annurev-chembioeng-100521-085846

[81] L. D. Ellis , N. A. Rorrer , K. P. Sullivan , M. Otto , J. E. McGeehan , Y. Román‐Leshkov , N. Wierckx , G. T. Beckham , Nat. Catal. 2021, 4, 539.

