# Supplementary material for: Chemically Recyclable Polyester Thermosets from Activated Adipic Acid and Renewable Polyols
Source: ChemSusChem. 2025 Sep 1;18(19):e202500880. doi: 10.1002/cssc.202500880 (PMC12487748; doi:10.1002/cssc.202500880)
Supplement: Supplementary file 1 — Supplementary Material [file CSSC-18-e202500880-s001.pdf]

Supporting Information (SI)

Chemically recyclable polyester thermosets from activated adipic acid and renewable polyols

Davide Rigo,[a], +, * Matteo Lorenzon, [a], + Jonas Simon, [b] Bennett Addison, [c] Alvise Perosa, [a] and Maurizio Selva[a],*

[a] Dr. D. Rigo, M. Lorenzon, Prof. A. Perosa, Prof. M. Selva
Department of Molecular Sciences and Nanosystems
Ca' Foscari University of Venice
Via Torino 155, 30172 Venice, Italy
E-mail: davide.rigo@unive.it; selva@unive.it

[b] Dr. Jonas Simon
Department of Chemistry
Institute of Chemistry of Renewable Resources, University of Natural Resources and Life Sciences Vienna (BOKU) Konrad-Lorenz-Strasse 24, 3430 Tulln, Austria

[c] Dr. Bennett Addison
Renewable Resources and Enabling Sciences Institute of Chemistry of Renewable Resources
Natural National Renewable Energy Laboratory
Golden, Colorado 80401, United States

+These Authors equally contributed to the work.

Corresponding authors: davide.rigo@unive.it
selva@unive.it

Table of contents

Experimental ... 3
Literature procedures for the synthesis of hyperbranched polyesters based on glycerol, sorbitol and adipic acid ... 7
Possible mechanism for the CLM synthesis ... 11
The synthesis and characterization of the crosslinking mixtures (CLMs) ... 11
The comparison of CLM obtained from iPAc and Ac_2O. ... 18
The recovery of the residual iPAc/Ac_2O and the volatiles by products from the CLMs synthesis ... 19
Differential scanning calorimetry (DSC) curves of the glycerol/sorbitol+CLM mixtures ... 22
The formation of volatiles and the mass balance during the pre-polymer synthesis and during the materials curing ... 23
The synthesis of elastomeric foams ... 36
FT-IR spectra ... 36
Differential scanning calorimetry (DSC) curves ... 52
Dynamic mechanical analysis (DMA) ... 52
The chemical recycling of the polyesters ... 53
The characterization of the recycled polyesters G_1 and S_1 ... 69
Thermo gravimetry analysis (TGA) ... 73
Tensile tests of the fresh materials ... 77
Tensile tests of the recycled materials ... 81
References: ... 83

Experimental

General

Reagents and solvents were commercially available compounds and were used as received unless otherwise stated. Glycerol, sorbitol, adipic acid, acetic anhydride (Ac_2O), isopropenyl acetate (iPAc), H_2SO_4, chloroform, MeOH-*d4*, DMSO-*d6*, $CDCl_3$, and NaOH were purchased from Sigma-Aldrich (Merck) and used without any further purification.

Synthesis of the crosslinking mixture (CLM)

CLM was synthesized by adjusting a method recently reported by us.[1] Accordingly, in a glass-flask, a mixture of adipic acid (AA; 200 g, 1.36 mol), isopropenyl acetate (iPAc; 2 equiv., 274 g) or acetic anhydride (Ac_2O; 2 equiv., 279.4 g), and H_2SO_4 (0.05 mol%, 67 mg) was set to react for: i) 1 h at reflux temperature of iPAc or Ac_2O and ii) 4 h at 80 °C under vacuum (p = 10 mbar) using a rotavapor to distill residual traces of iPAc/Ac_2O and other compounds as acetone or AcOH formed during the reaction. The CLM was isolated in a quantitative molar yield with respect to adipic acid, and characterized by ^{1}H, ^{13}C and HSQC NMR analyses.

Synthesis of glycerol-/sorbitol-based polyesters

In a glass-flask, the chosen sugar alcohol (glycerol or sorbitol, 20-50 g) was mixed with the CLM in different weight ratios, in the ranges glycerol:sorbitol:CLM = 0-1:0-1:1 wt%:wt%:wt% (details are in Figure 4D). A homogeneous highly viscous pre-polymer was obtained at 150 °C over a time of 3-5 h that depended on the reactant sugar alcohol used and its amount (Figure 4D). The pre-polymer was then poured into a previously warmed silicon mold and further heated at 150 °C for 16 h. Once cooled to rt, a thermoset polyester (82-89 wt% yield based on the total amount of reactants) was isolated. The material was characterized for its structural and mechanical properties by tensile tests, dynamic mechanical analysis (DMA), high-resolution magic angle spinning (HR-MAS) and solid-state (ss) NMR, thermo-gravimetry analysis (TGA), differential scanning calorimetry (DSC), and Fourier-transformed infrared (FT-IR) spectroscopy.

Evaluation of the mechanical properties

Tensile strength, strain and Modulus. Tensile properties of samples were measured using an Instron Model 3345 mechanical tester at room temperature. The thickness, width and length of each sample were 1.5 mm, 20 mm, 100 mm, respectively, and the crosshead speed was 5 mm·min^{-1}. A minimum of three samples were tested for each measurement.

Characterization of the polyesters

NMR analysis during polymer synthesis and depolymerization

^{1}H- and ^{13}C-NMR spectra of the polymers were recorded on a 400-MHz Bruker instrument (FT 400 MHz, ^{1}H; 100 MHz, ^{13}C) at ambient temperature. Chemical shifts were referenced to internal solvent resonances and reported as parts per million (ppm) relative to tetramethylsilane (TMS). $CDCl_3$ and DMSO-d_6 were used as the solvents.

Solid state ^{13}C NMR of dry polymers

Solid state ^{13}C cross-polarization/magic angle spinning (CP/MAS) NMR spectra were recorded on a Bruker Avance III HD 400 spectrometer at a resonance frequency of 400 MHz and 100 MHz for ^{1}H and ^{13}C, respectively, with a 4 mm dual broadband CP/MAS probe. Data were acquired at room temperature with a spinning rate of 12 kHz, a CP contact time of 2 ms, and SPINAL-64 ^{1}H decoupling.

NMR analysis of swollen polymers

High-Resolution Magic Angle Spinning (HR-MAS) data were collected on a 600 MHz (14.1 Tesla) Bruker AV4 NEO NMR spectrometer equipped with a 4-mm HR-MAS probe with z-gradients. All samples were swollen in DMSO-d_6 and packed into polychlorotrifluoroethylene (PCTFE, KelF, Bruker Biospin) HR-MAS inserts, sealed with provided plug and screw-in caps then loaded into 4 mm zirconium rotors with a Kel-F drive tip. Samples were spun at the magic angle at 5 kHz MAS for all HR-MAS measurements. Solution-state NMR sequences were used to collect ^{1}H, ^{13}C, HSQC, and HMBC data. All spectra were processed and visualized using MestreNova version 14, and chemical shifts were all referenced internally to the solvent signal (2.49, 39.5 ppm).

^{13}C solid-state NMR (ssNMR) data were collected on the same spectrometer as for HR-MAS but using a 4 mm PhoenixNMR (Loveland, CO) HXY MAS probe configured in HC double resonance mode. The same DMSO-d_6 swollen samples for HR-MAS were removed from the inserts and then centerpacked into 80 mL RevolutionNMR (Loveland, CO) rotors with silicon disks sandwiched between PCTFE spacers to create a tight seal. Samples were spun at 10 kHz for all ssNMR measurements. Three ^{13}C ssNMR measurements were performed on each sample: 1) Cross-polarization magic angle spinning (CP-MAS) to highlight mostly rigid carbons, 2) Direct-polarization (DP-MAS) using a moderate recycle delay of 5 seconds to highlight all carbons, and 3) 1D INEPT to highlight only highly dynamic protonated carbon types. Typical radiofrequency (RF) field strengths were 62.5 kHz RF for cross-polarization and hard pulses (4.0 ms 90s), and 80 kHz for ^{1}H decoupling. To be consistent with ssNMR best practices, ^{13}C chemical shifts were referenced externally to TMS at 0 ppm by setting the downfield Adamantane signal to 38.48 ppm.[2] All data were processed and visualized using MestreNova version 14.

Thermogravimetry analysis (TGA)

TGA was performed on a TG 209F1 instrument (NETZSCH). Each sample (4 to 10 mg) was equilibrated at room temperature and heated at 10 °C min^{-1} to 600 °C under

nitrogen. The reported $T_{d5\%}$ and $T_{d50\%}$ values represent the temperatures at which 5% and 50% of the mass is lost, respectively.

Differential scanning calorimetry (DSC)

DSC thermograms were measured under nitrogen on a DSC 214 Polyma (NETZSCH). Each sample (ca 10 mg) was added to a pierced aluminum crucible and passed through a heat−cool−heat cycle at 10 °C min^{-1}. The temperature ranged from -80 (min) to 300 °C (max), and the reported data are from the second heating cycle. NETZSCH's spectroscopy software Proteus® (v 8.0.2) was used for processing DSC data.

Fourier transformed Infrared (FTIR) spectroscopy

FTIR spectroscopy was conducted using a Frontier FTIR spectrophotometer (PerkinElmer) operating in attenuated total reflection (ATR) mode. All spectra were recorded by accumulation of 16 scans per sample at a resolution of 4 cm^{-1} over the range of 4000-600 cm^{-1}.

Gel Fraction Measurement

The gel fraction of the thermosets was measured by solvent soaking. In a typical procedure, the sample of choice (W_I, *ca.* 0.5 g) was soaked in acetone (25 mL) at room temperature for 2 days. The insoluble residue was filtered and then vacuum-dried to a constant weight (T = 40 °C, *p* = 5 mbar). The gel fraction (W_G) was determined gravimetrically. The measurements were repeated three times. The % of gel fraction was calculated as follows: $(W_G/W_I)\cdot 100$.

Chemical recycling of the polyesters

Depolymerization tests via methanolysis. Samples G_1 and S_1 in Table 1 (*vide infra*) obtained by the reaction of AA with glycerol and sorbitol, respectively, were used for this study. In a typical experiment, a round-bottom flask was charged with the polymer (50 g) and 1 wt% NaOH solution in MeOH (200 mL), and heated at 50 °C for 1 h, under magnetic stirring (400 rpm). Thereafter, the mixture was neutralized with 10mol% HCl, and the solvent rotary evaporated (*p* = 5 mbar). A heterogeneous mixture of adipic acid methyl esters and sugar alcohols was obtained. This mixture was fractionated by liquid/liquid extraction using water and 2-methyl tetrahydrofuran (2-MeTHF) in 1:1 v/v (100 mL each). The organic phase was rotary evaporated to obtain a light brown oil composed of adipic acid methyl esters (yield: 26.3 g, 99 mol%, and 25.4 g, 99mol% for G_1 and S_1, respectively). The aqueous phase was treated with Amberlyst-36 for 1 h at room temperature under stirring to remove the NaCl formed during the neutralization of NaOH. Then, the resin was filtered, and the water was removed by rotary evaporation (*p* = 5 mbar). A slightly brown highly viscous liquid composed of the corresponding sugar alcohols (yield: 25.3g, 99%, and 19.5g, >99% for G_1 and S_1, respectively) was obtained. The mixtures of such products were characterized by GC/MS, ^{1}H-NMR, and ^{13}C-NMR, using benzoic acid as an internal standard for NMR analyses (*vide infra*).
Synthesis of recycled polyesters. The mixture of methyl adipates obtained from the above-described depolymerization of G_1 and S_1 samples (26.3 g and 25.4 g, respectively) was hydrolyzed in a 200 mL autoclave with a 0.1% (v/v) H_2SO_4 aq. (100

mL) for 4 hours at 150 °C. Then, water was removed by rotary evaporation (*p* = 5 mbar) and the catalyst via a fast SiO_2-filtration. Adipic acid was obtained in a >99% yield (22.3-22.4 g): its structure was confirmed by ^{1}H- and ^{13}C-NMR. Thereafter, the procedures described above for the synthesis of the crosslinking mixture (CLM) and the transesterification of the CLM with glycerol and sorbitol were followed using the recovered compounds, both adipic acid and sugar alcohols in 1:1 wt ratio. The resulting thermoset recycled polymers were then characterized by TGA, DSC, and FT-IR.

Literature procedures for the synthesis of hyperbranched polyesters based on glycerol, sorbitol and adipic acid

Table S1. Reported procedures for the synthesis of crosslinked polyesters using glycerol, sorbitol and adipic acid as the monomers.

Entry	Reagents	T, t (°C, h)	Catalyst	Gel fraction (%)	Tg[a]	σ[a] (MPa)	ε[b] (%)	E[c] (MPa)	Ref.
1	Glycerol, adipic acid	**Step 1:** 100, n.a. **Step 2:** 150, n.a. **Step 3:** 50, >48	dibutyltin dilaurate	n.a.	n.a.	n.a.	n.a.	n.a.	[3]
2	Glycerol, adipic acid	140, n.a.	dibutyltin oxide	Soluble in DMSO	n.a.	n.a.	n.a.	n.a.	[4]
3	Glycerol, adipic acid	**Step 1:** 100, n.a. **Step 2:** 150, n.a. (p = 100 mbar)	dibutyltin oxide	Soluble in DMSO, THF	n.a.	n.a.	n.a.	n.a.	[5]
4	Glycerol, divinyl adipate	**Step 1:** 40-70, 24 **Step 2:** 90, 1	Novozyme 435	Soluble in acetone, THF	-49 to -34	n.a.	n.a.	n.a.	[6]
5	Glycerol, adipic acid, 1,8-octanediol	**Step 1:** 115, n.a. **Step 2:** 90, 2 **Step 2:** 90, 42 (p = 40 mbar)	Novozyme 435	Soluble in THF, MeOH	n.a.	n.a.	n.a.	n.a.	[7]
6	Glycerol, adipic acid, 1,8-octanediol	**Step 1:** 80, 4 (p = 400-200 mbar)	Novozyme 435, $Sc(OTf)_3$,	Soluble in $CHCl_3$ except with $Sc(OTf)_3$	n.a.	n.a.	n.a.	n.a.	[8]

		**Step 2:** 80-100, 4-9 (*p* = 40 mbar)	Nf_2NH, DPP, TBD	which led to an insoluble gel					
7	Glycerol, divinyl adipate, indomethacin	**Step 1:** 50, 10 (*p* = 300 mbar) **Step 2:** 95, 1	Novozyme 435	Soluble in $CHCl_3$	-27 to 51	n.a.	n.a.	n.a.	[9]
8	Glycerol, divinyl adipate, stearoyl chloride	**Step 1:** 40-70, 24 **Step 2:** 90, 1 **Step 3:** 66, 3-4 **Step 4:** 50, 48 (vacuum; *p* = n.a.)	Novozyme 435, pyridine	Soluble in acetone, THF	-54 to -28	n.a.	n.a.	n.a.	[10]
9	Glycerol, divinyl adipate, stearoyl chloride	**Step 1:** 50, 0.5 **Step 2:** 50, 24 **Step 3:** 100, n.a. (vacuum; *p* = n.a.) **Step 4:** 66, 2	Immobilized Novozyme 435, pyridine	Soluble in acetone, THF	n.a.	n.a.	n.a.	n.a.	[11]
10 (R2)	Glycerol, adipic acid, ethylene glycol	**Step 1:** 150, 17 **Step 2:** 150, 4, (*p* = 40 mbar) **Step 3:** 120, 72-120	Cat.-free	92.2-99.6	-30 to -3	0.16-0.68	28.5-193.8	0.07-5.0	[12]
11 (R1)	Glycerol, succinic, adipic, sebacic acid, 1,4-butanediol	**Step 1:** 180, 3, 1 **Step 2:** 225, 3, (*p* = 10 mbar)	Cat.-free	88.7-95.5	-48 to -38	0.36-2.42	542-1728	0.08-0.86	[13]
12	Sorbitol, adipic acid	95-120, 48	PTSA, Novozyme 435, K_2CO_3	Soluble in water	n.a.	n.a.	n.a.	n.a.	[14]
13	Sorbitol, succinic, adipic, suberic,	**Step 1:** 120-140, n.a.	PTSA	Soluble in $CHCl_3$	n.a.	n.a.	n.a.	n.a.	[15]

	sebacic acids, 1,4-butanediol, propionic acid	**Step 2:** 160, n.a. **Step 1:** 180-190, n.a.							
14	Sorbitol, adipic acid/dimethyl adipate, 1,10-/1,8-decanediol, succinic, suberic, sebacic, dodecandioc, tetradecanedioic acids	90-130, 2-24	Novozyme 345, CALB	Soluble/partially soluble in DMSO, acetone, $CHCl_3$	*ca.* - 20	n.a.	n.a.	n.a.	[16]
15	Sorbitol, 1,3-propanediol, dimethyl adipate	60-140, 26-103 (vacuum, *p* = n.a.)	CAL-B, TBD, $Sc(OTf)_3$, DBTO	Insoluble gel (gel fraction n.a.) or soluble in pyridine, $CHCl_3$	-59 to -48	n.a.	n.a.	n.a.	[17]
16	Sorbitol, tetrahydro phthalic anhydride, diethylene glycol	**Step 1:** 150, 1 **Step 2:** 200, 5-6	ZnOAc	n.a.	n.a.	n.a.	n.a.	n.a.	[18]
17	Sorbitol, succinic acid, ethylene glycol, 1,4-butanediol, 1,6-hexanediol	**Step 1:** 150, 2 **Step 2:** 80, 48	Cat.-free	Soluble in acetone, 1,4-dioxane, DMSO, $CHCl_3$	-35 to -16	0.014-10.61	18.5-38.7	20.2-554.8	[19]
18	Sorbitol, succinic, adipic, suberic, sebacic acid, diethylene glycol	**Step 1:** 150, 2 **Step 2:** 150, 120	Cat.-free	Soluble in acetone, 1,4-dioxane, DMSO, $CHCl_3$	n.a.	0.57-0.78	15-87	1.77-3.84	[20]

19	Glycerol, sorbitol, adipic acid	90, 48	Novozyme 435	Soluble in water, DMF	n.a.	n.a.	n.a.	n.a.	[21]
20	Glycerol, sorbitol, adipic acid	90, 48 (*p* = 50 mbar)	Novozyme 435	Soluble in $CHCl_3$	-28 to 7	n.a.	n.a.	n.a.	[22]

[a]Glass transition temperature (T_g). [a]Tensile stress (σ). [b]Tensile strain (ε). [c]Young's modulus (E). n.a. = not available. ■ Glycerol-based ■ Sorbitol-based ■ Glycerol+sorbitol-based

Possible mechanism for the CLM synthesis

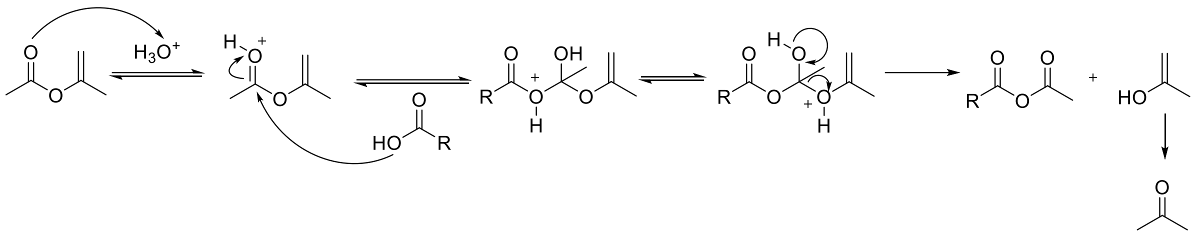

Figure S1. Proposed mechanism for acetone formation during adipic acid activation with iPAc: acid-catalyzed nucleophilic attack of the carboxylic acid to isopropenyl acetate, intramolecular rearrangement followed by keto-enol tautomerization of the isopropenyl group to yield acetone.

The synthesis and characterization of the crosslinking mixtures (CLMs)

The activation of adipic acid (AA) was performed by adapting our recently reported protocol.[1]

The synthesis of the CLM using acetic anhydride (Ac_2O). A mixture of AA (200 g, 1.36 mol), acetic anhydride (Ac_2O; 2 equiv., 279.4 g), and H_2SO_4 (0.01 mol%, 13 mg) was set to react for 1h at T = reflux (Step 1), followed by vacuum distillation of the volatiles by-products for 4h at T = 80 °C and p = 10 mbar (Step 2) (Figure S1, top). The products distribution was evaluated by ^{1}H NMR with 1,4-dioxane as the internal standard.

During the reaction between AA and Ac_2O, acetyl anhydrides are converted into the desired adipoyl anhydrides, while a low number of COOH functionalities is constantly present in the mixture (Figure S1). Nevertheless, the formation of adipic anhydride (**1**) and the conversion of COOH groups were observed in the Ac_2O-CLM while being stored at room temperature over time (see Figure S6-8). At the end of the reaction the composition of the CLM included adipoyl anhydrides (AdOCOOAd) and acetyl anhydrides (AcOCOOAd) in amounts of 7.9 mmol·g^{-1}, and 4.2 mmol·g^{-1}, respectively, together with a small amount of COOH functional groups (0.9 mmol·g^{-1}).

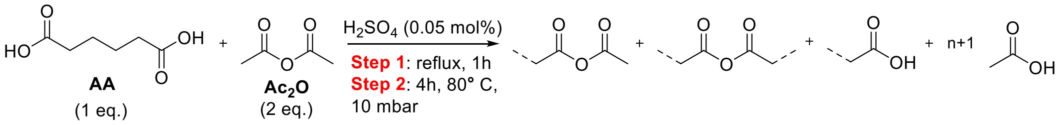

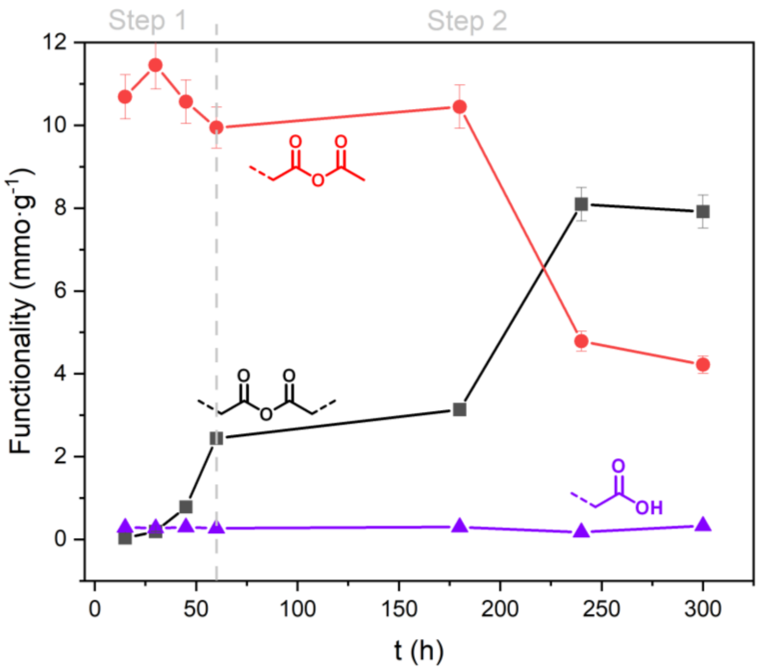

Figure S2. Top: The synthesis of the adipic acid (AA)-based crosslinking mixture (CLM) with acetic anhydride (Ac_2O). **Bottom:** The trend of the reaction over the time. -■- Symmetric anhydrides -●- Acetyl anhydrides -▲- Carboxylic acids -♦- Cyclic anhydrides. The results have been evaluated by ^{1}H NMR with dioxane as the internal standard.

Table S2 summarizes the results obtained after 300 min and the spectra over time from the reaction with iPAc and Ac_2O are reported in Figures S2 and S3, respectively.

Table S2. The type and amount of functionalities in the CLMs.

Entry	Reagent	Functionality (mmol/g)[a]					
		Isopropenyl esters	Acetyl anhydrides	Adipoyl anhydrides	Cyclic anhydrides	Carboxylic acids	Sum[b]
1	iPAc	Traces	6.4	5.4	0.3	Traces	12.1
2	Ac_2O	-	4.2	7.9	<0.05	0.9	13.0

Reaction conditions: adipic acid (AA; 200 g, 1.36 mol), isopropenyl acetate (iPAc; 2 equiv., 274 g) or acetic anhydride (Ac_2O; 2 equiv., 279.4 g), and H_2SO_4 (0.01 mol%, 13 mg). Step 1: t = 1h, T = reflux; step 2: 4h, T = 80 °C, *p* = 10 mbar. [a]Evaluated by ^{1}H NMR with 1,4-dioxane as the internal standard. [b]Sum of all the functionalities in the CLMs.

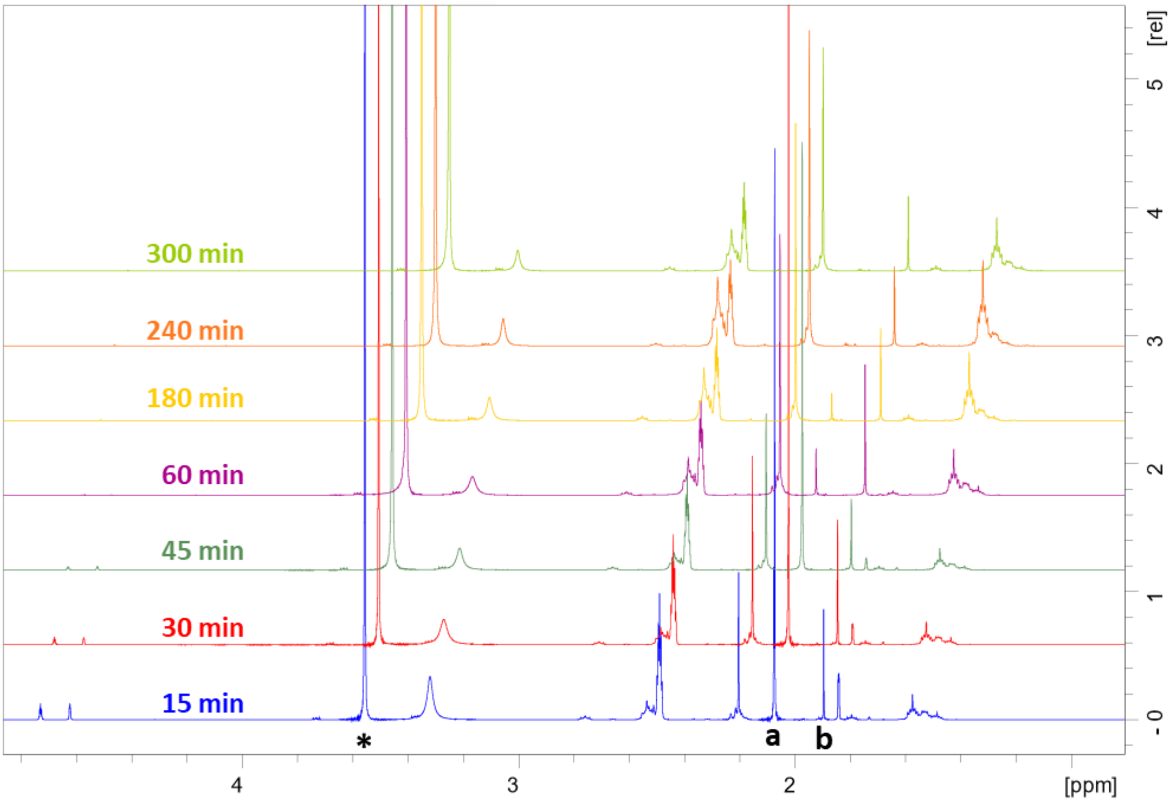

Figure S3. ^{1}H spectra of the CLM-iPAc over time from the reaction between AA (1 eq.) and iPAc (2 eq.) under H_2SO_4 catalysis (0.05 mol%). Step 1: T = reflux, t = 1h. Step 2: T = 80 °C, t = 4h. The ***** indicates the signal of 1,4-dioxane as the internal standard. The **a** and **b** are referred to the acetone and AcOH by-products of the reaction, respectively. Spectra were recorded in DMSO-d_6.

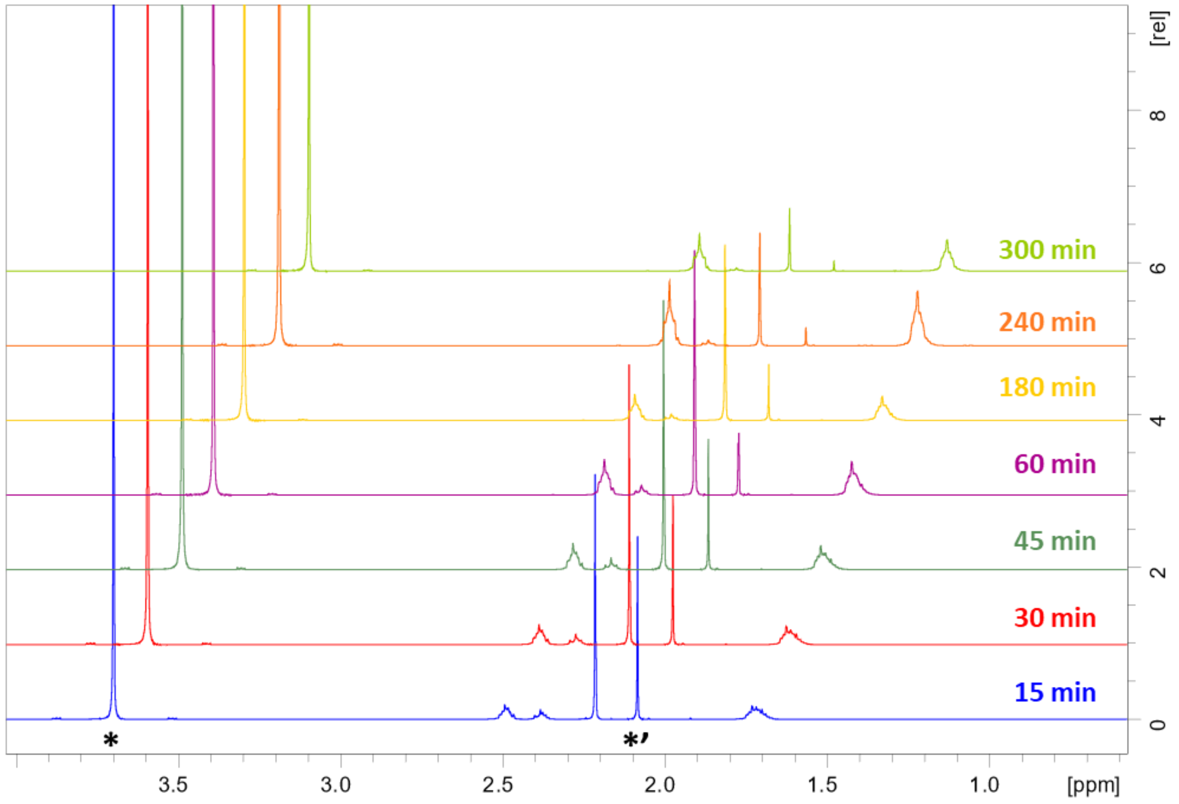

Figure S4. ^{1}H NMR spectra of the CLM-Ac_2O over time from the reaction between AA (1 eq.) and Ac_2O (2 eq.) under H_2SO_4 catalysis (0.05 mol%). Step 1: T = reflux, t = 1h. Step 2: T = 80 °C, t = 4h. The ***** indicates the signal of 1,4-dioxane as the internal standard and the ***'** indicates the signal of the AcOH by-product. Spectra were recorded in $CDCL_3$:DMSO-d_6 = 1:0.5 v/v.

A comprehensive NMR characterization (^{1}H, ^{13}C and HSQC) of the CLMs was performed at the end of the reaction. The spectra are reported in Figures S4-9.

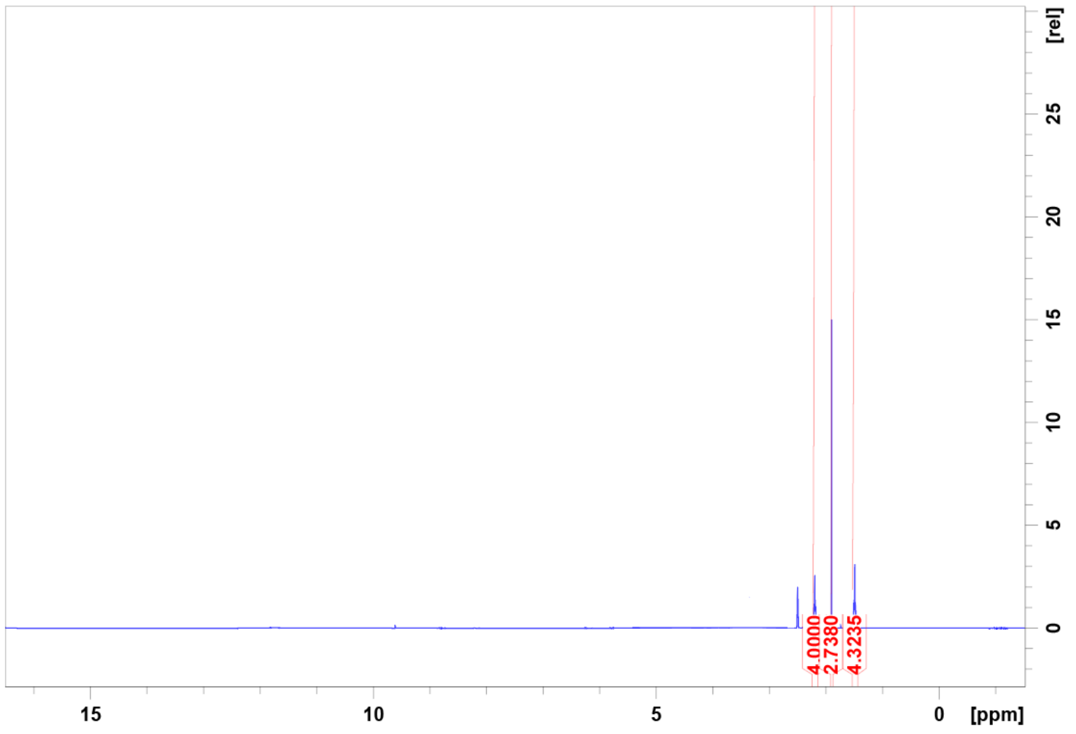

Figure S5. ^{1}H NMR spectrum of the CLM-iPAc after one week of storage (400 MHz, 298 K, $CDCL_3$:DMSO-d_6 = 1:0.5 v/v).

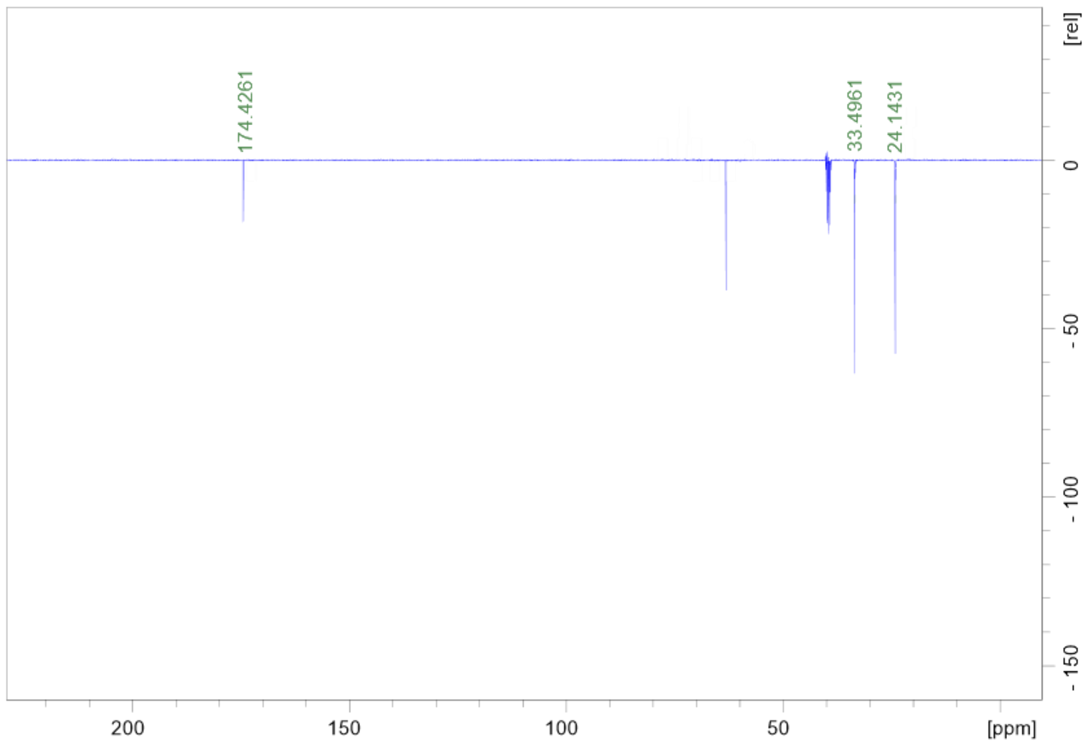

Figure S6. ^{13}C NMR spectrum of the CLM-iPAc after one week of storage (100 MHz, 298 K, $CDCL_3$:DMSO-d_6 = 1:0.5 v/v).

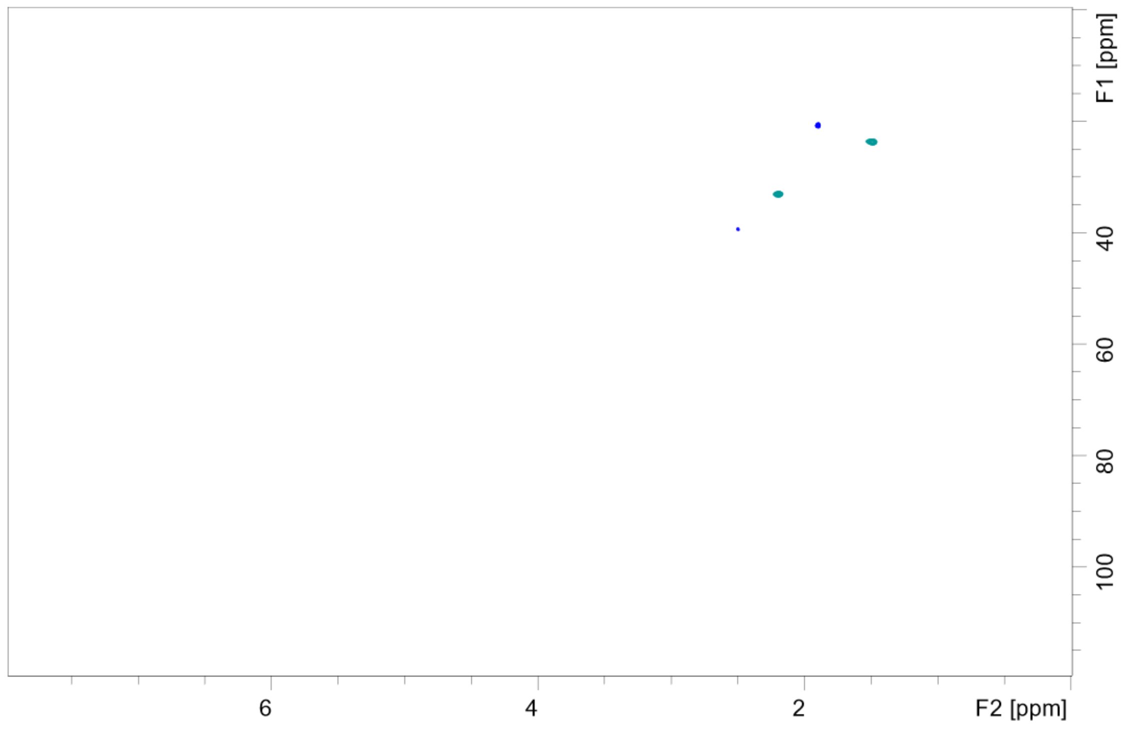

Figure S7. HSQC NMR spectrum of the CLM-iPAc after one week of storage (298 K, $CDCL_3$:DMSO-d_6 = 1:0.5 v/v).

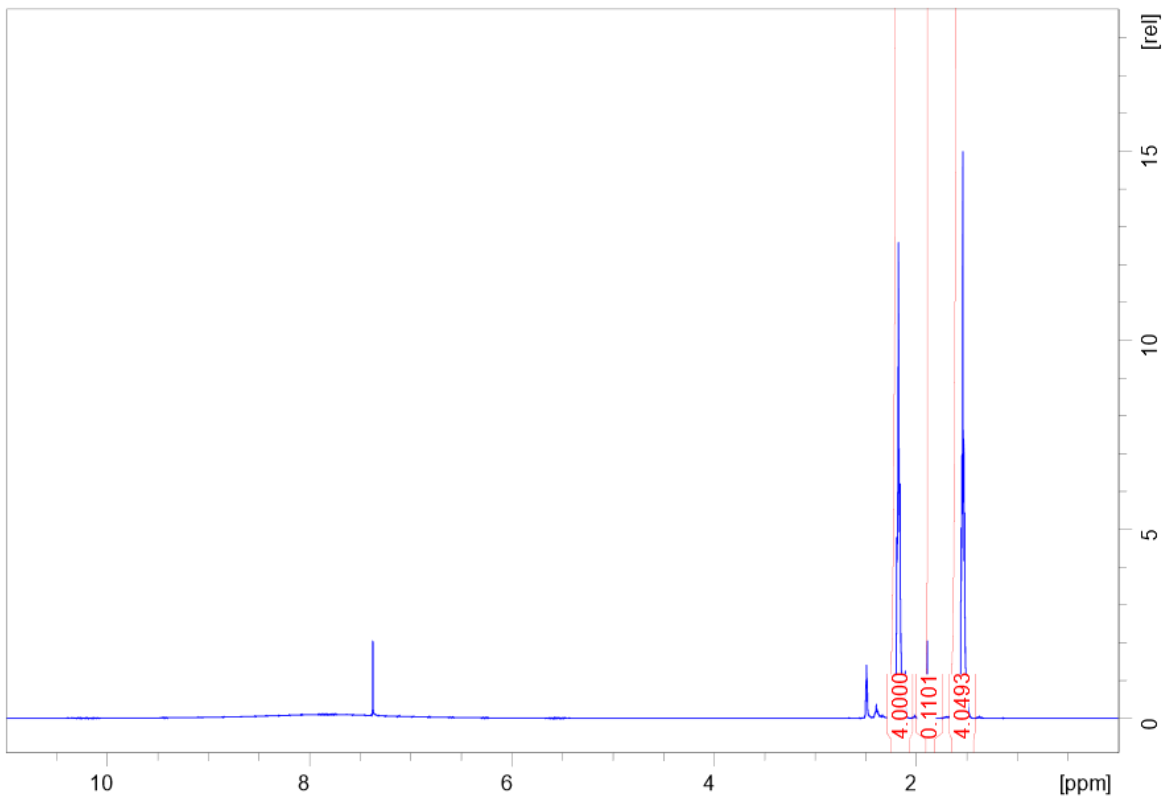

Figure S8. ^{1}H NMR spectrum of the CLM-Ac_2O after one week of storage (400 MHz, 298 K, $CDCL_3$:DMSO-d_6 = 1:0.5 v/v).

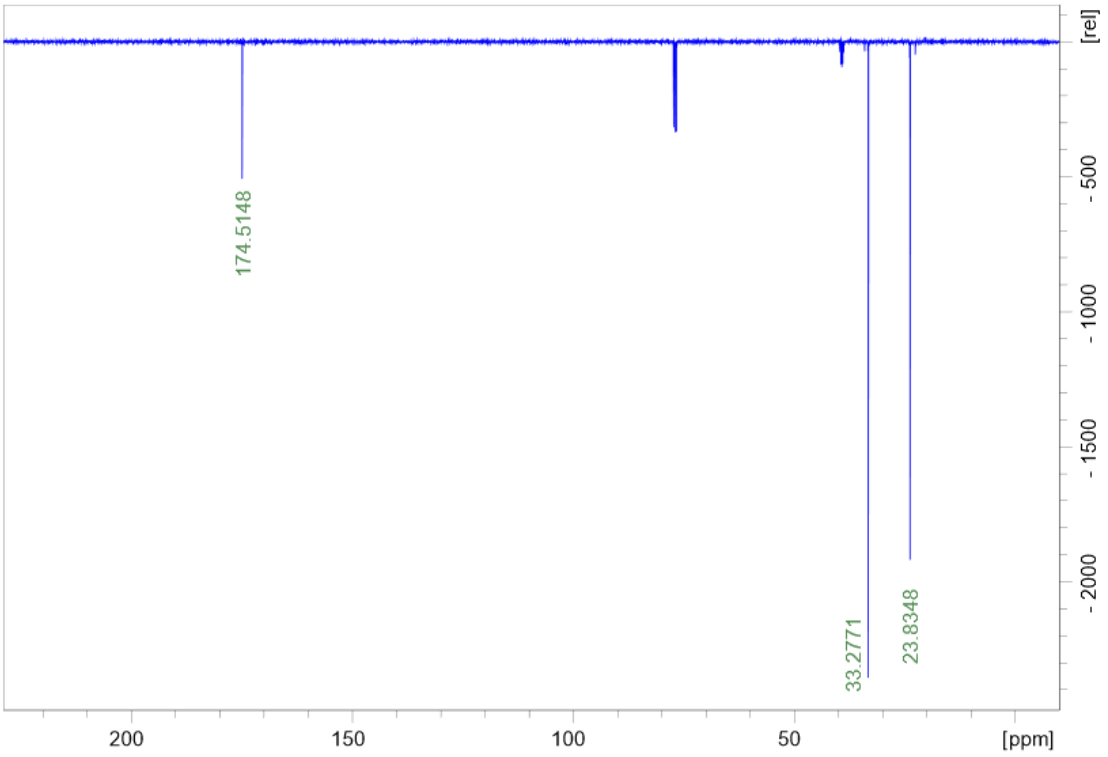

Figure S9. ^{13}C NMR spectrum of the CLM-Ac_2O after one week of storage (100 MHz, 298 K, $CDCL_3$:DMSO-d_6 = 1:0.5 v/v).

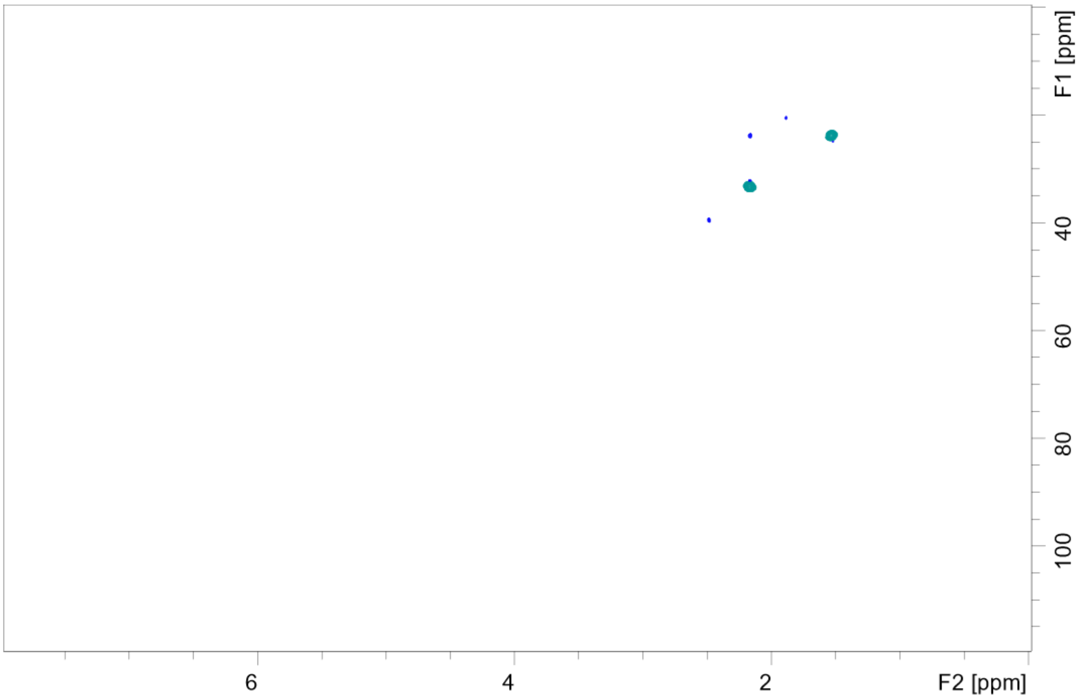

Figure S10. HSQC NMR spectrum of the CLM-Ac_2O after one week of storage (298 K, $CDCL_3$:DMSO-d_6 = 1:0.5 v/v).

The comparison of CLM obtained from iPAc and Ac_2O.

Conditions illustrated in Figure S10 and the accompanying Table were used to prepare four polyesters samples. After the synthesis of CLM derived from iPAc and Ac_2O, CLM_i and CLM_A, respectively, these mixtures were set to react with glycerol or sorbitol using a SA (sugar alcohol):CLM weight ratio of 1:1. The synthesis followed the two steps detailed in the section “Synthesis of the glycerol-/sorbitol-based polyesters” in main text of the paper: particularly, prepolymerization and polymerization were carried out at 150 °C for 3 h and 16 h, respectively. The resulting four samples were labelled as G_{i1} and G_{A1}, from glycerol, and S_{i1} and S_{A1}, from sorbitol, respectively.

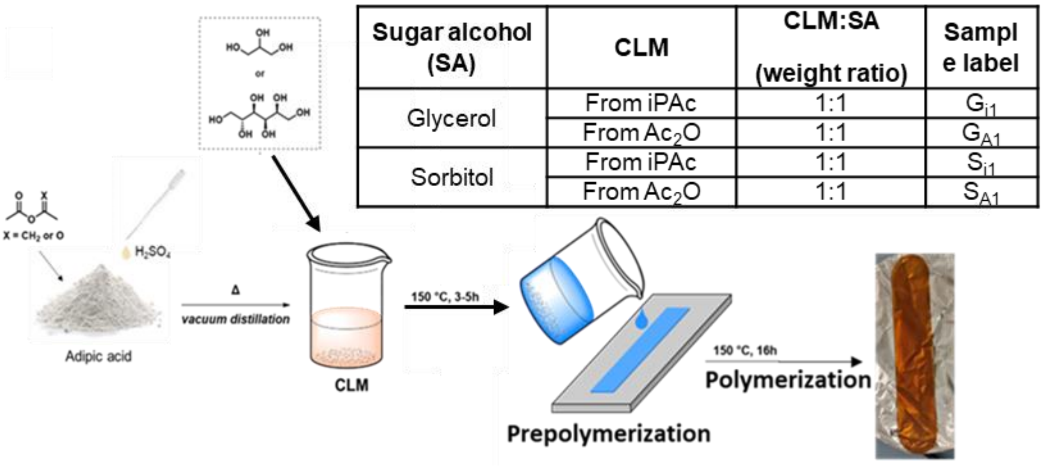

Sugar alcohol (SA)	CLM	CLM:SA (weight ratio)	Sample label
Glycerol	From iPAc	1:1	G_{i1}
	From Ac_2O	1:1	G_{A1}
Sorbitol	From iPAc	1:1	S_{i1}
	From Ac_2O	1:1	S_{A1}

Figure S11. The preparation of glycerol and sorbitol poly-adipates using a CLM based on iPAc and Ac_2O, respectively. The SA (sugar alcohol):CLM = weight ratio was 1:1 in all cases. The picture shows a typical polyester sample

The polyesters were characterised by FT-IR, DSC, and TGA. Results are illustrated in Figures S11 and S12. The substantial overlap of the normalized FT-IR spectra in Figure S11A and S11B confirmed the structural similarities between G_{i1} and G_{A1}, and S_{i1} and S_{A1}, respectively. The presence of an intense signal at 1750 cm^{-1} typical of the carbonyl stretching of aliphatic esters together with the almost complete absence of peaks in the range 3000-3600 cm^{-1} in all the spectra indicated that esterification of OH groups occurred. The similar intensities (two-by-two G_{i1} vs. G_{A1}, and S_{i1} vs. S_{A1}) of the latter peaks in the normalized spectra suggest that the degree of esterification is similar in both the glycerol- and sorbitol-based polyesters. DSC curves of samples G_{i1} and G_{A1} (Figure S12A), and S_{i1} and S_{A1} (Figure S12B) show comparable T_g (-0.8 and 0.4 for G_{i1} and G_{A1}, respectively; 39.5 and 41.1 for S_{i1} and S_{A1}, respectively) values for the polyesters synthesized from CLM_i and CLM_A. This indicates that the use of more benign iPAc in place of Ac_2O for the preparation of the CLM does not affect the thermal properties of the thermosets.

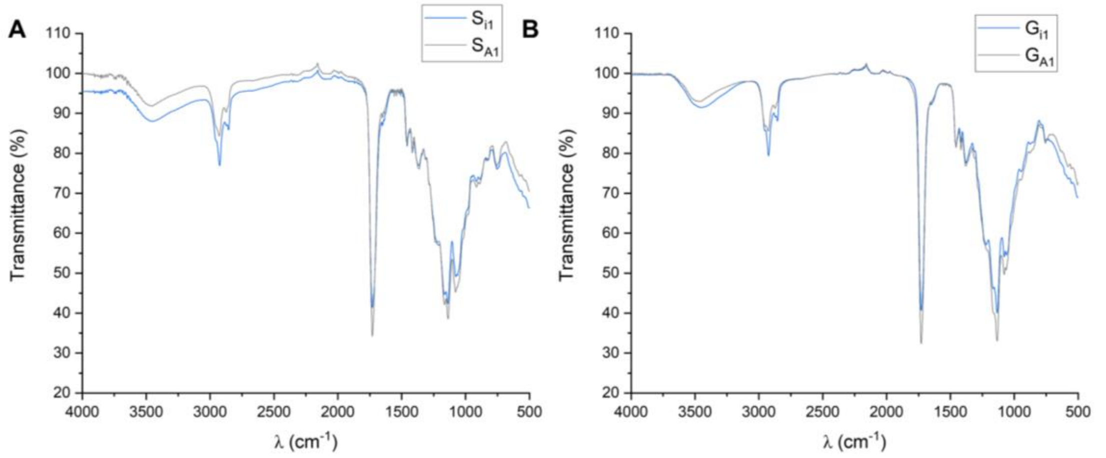

Figure S12. A) FT-IR spectra of glycerol-based polyesters synthesized from CLM$_i$ (G$_{i1}$) and CLM$_A$ (G$_{A1}$). B) FT-IR spectra of sorbitol-based polyesters synthesized from CLM$_i$ (S$_{i1}$) and CLM$_A$ (S$_{A1}$).

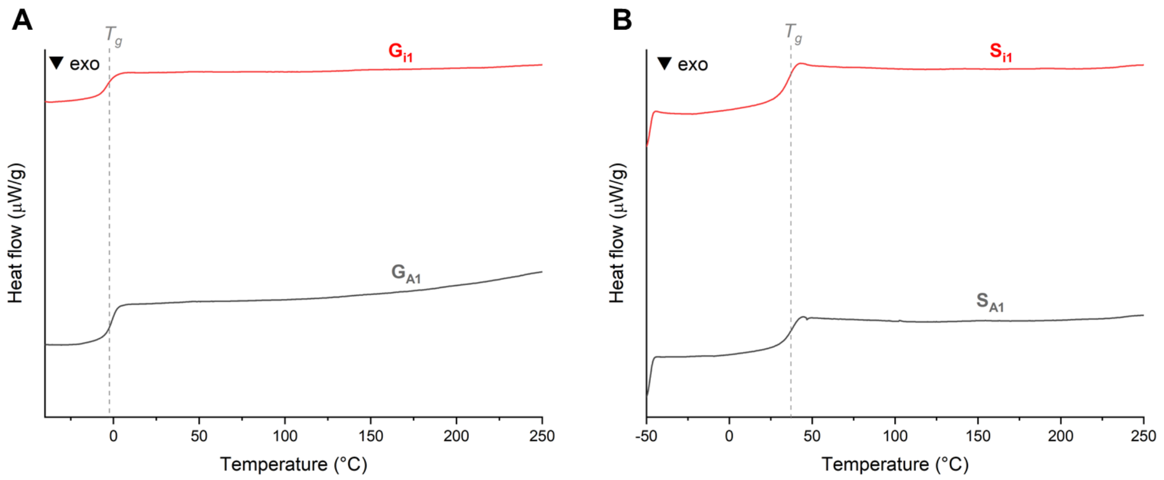

Figure S13. A) DSC curves of glycerol-based polyesters synthesized from CLM$_i$ (G$_{i1}$) and CLM$_A$ (G$_{A1}$). B) DSC curves of sorbitol-based polyesters synthesized from CLM$_i$ (S$_{i1}$) and CLM$_A$ (S$_{A1}$).

The recovery of the residual iPAc/Ac$_2$O and the volatiles by products from the CLMs synthesis

The reaction for the synthesis of the CLMs both using iPAc and Ac$_2$O as the activators was scaled up to 500g. To confirm the mass balance from the reaction, the eventual non-reacted iPAc/Ac$_2$O together with the acetone and AcOH (and possible others) co-products from the reaction were isolated by vacuum distillation during the 2nd step for the CLMs synthesis. The reaction conditions are reported in Figure 3 (main text). To summarize, a mixture of AA (500g, 3.4 mol) with either iPAc (684g, 2 equiv.) or Ac$_2$O (698g, 2 equiv.) under H_2SO_4 (0.16g, 0.05 mol%) catalysis were set to react at reflux temperature for 1h (Step 1) followed by vacuum distillation of the volatiles for 4h at T = 80 °C and p = 10 mbar (Step 2). The vacuum distillation was continuously performed by rotary evaporation (T = 80 °C and p = 10 mbar) and the mixtures of volatile products were collected to evaluate the yields, the mass balance and the volatiles composition.

Following that procedure, in the case of the reaction between AA and iPAc the iPAc-CLM and the volatiles were isolated in 42 wt% and 53 wt% yield, respectively, with respect to the total amount of initial reagents (AA and iPAc) with a mass balance of 95 wt%. The volatiles mixture comprised iPAc:Ac_2O:acetone:AcOH = 1:4.4:4.0:3.4 (mol:mol:mol:mol). The formation of Ac_2O during the reaction was expected, as we recently observed.[23,24] Similarly, from the reaction between AA and Ac_2O the Ac_2O-CLM and the volatiles were isolated in 45 wt% and 53 wt%, respectively, with respect to the total amount of initial reagents (AA and iPAc) with a mass balance of 98%. The volatiles mixture composition was Ac_2O:AcOH = 1:1.8 (mol:mol). The mixtures composition was evaluated by ^{1}H and ^{13}C NMR (see Figures S13-S16).

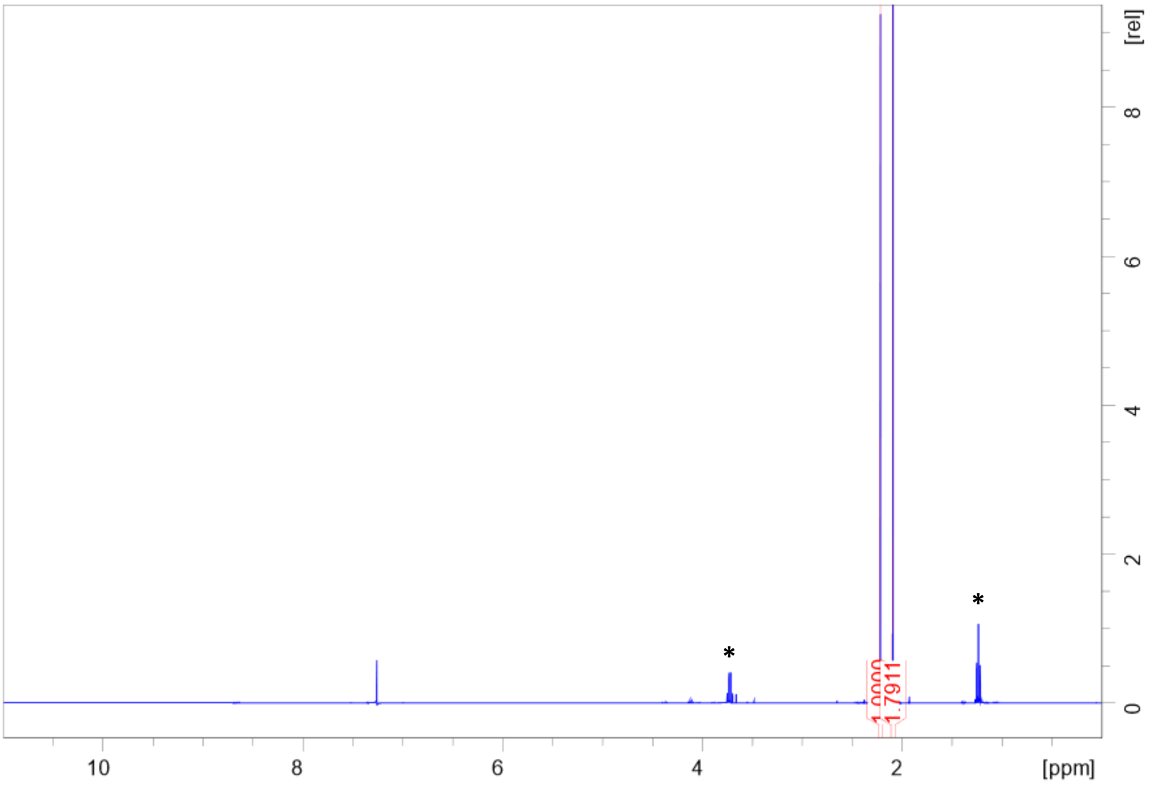

Figure S14. ^{1}H NMR spectrum of the volatiles mixture collected from the reaction of AA and Ac_2O. The * indicates the residual ethanol used to clean the NMR tubes. Solvent: $CDCl_3$.

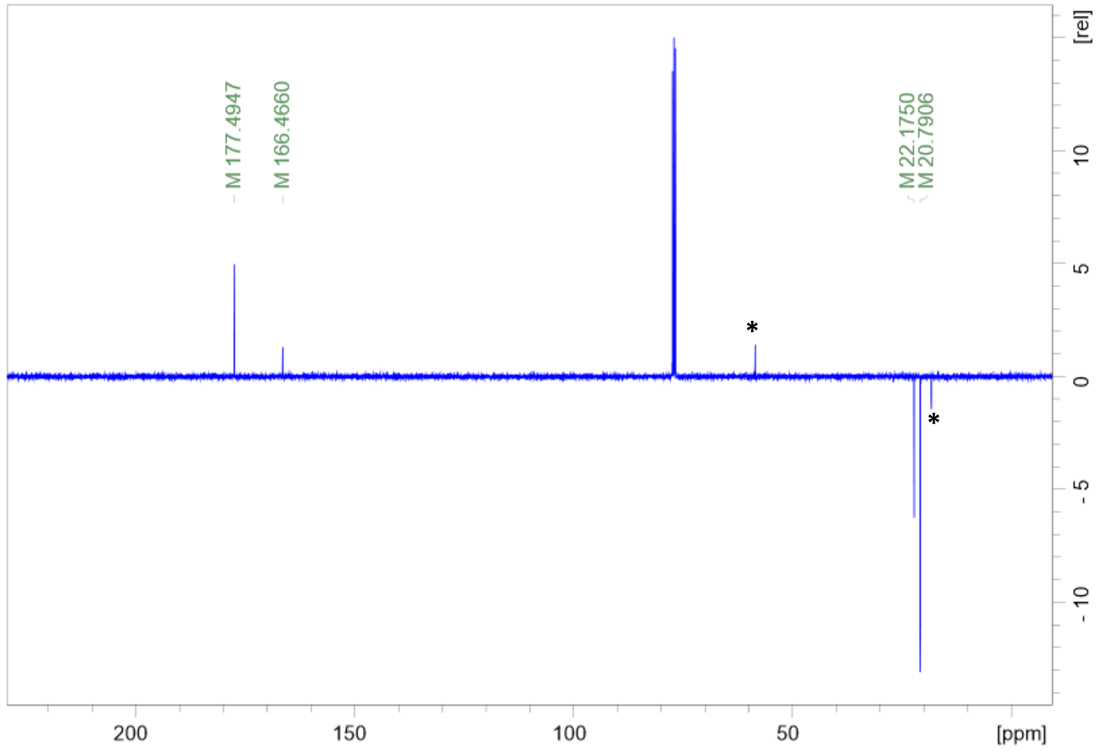

Figure S15. ^{13}C NMR spectrum of the volatiles mixture collected from the reaction of AA and Ac_2O. The * indicates the residual ethanol used to clean the NMR tubes. Solvent: $CDCl_3$.

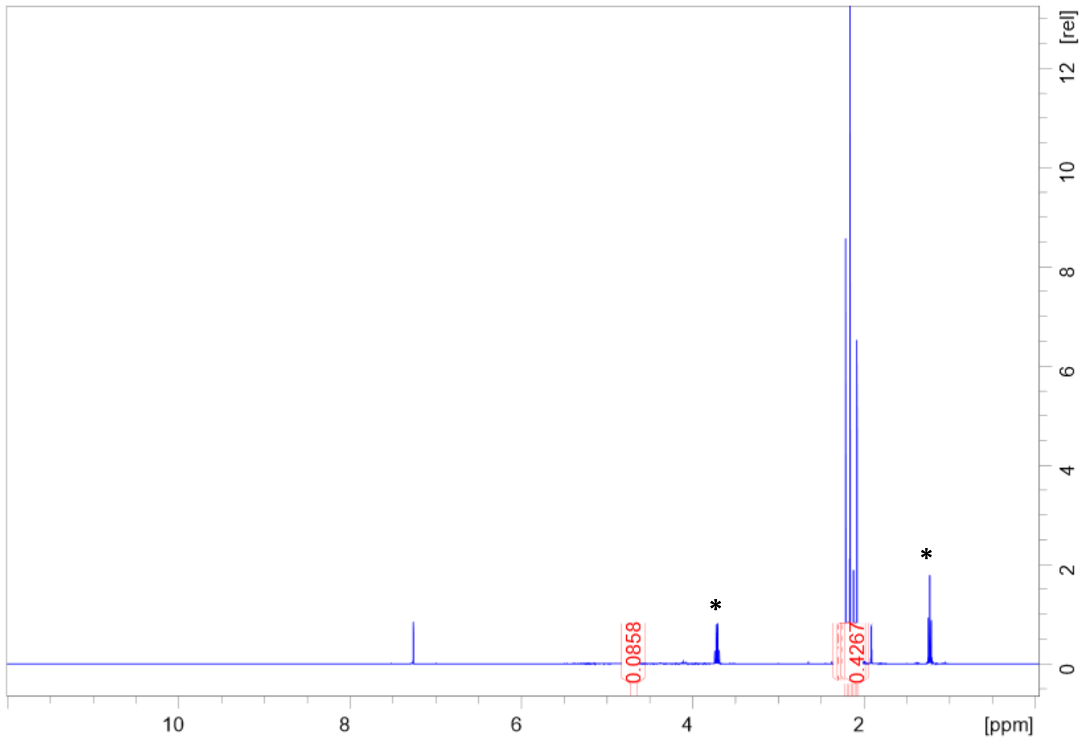

Figure S16. ^{1}H NMR spectrum of the volatiles mixture collected from the reaction of AA and iPAc. The * indicates the residual ethanol used to clean the NMR tubes. Solvent: $CDCl_3$.

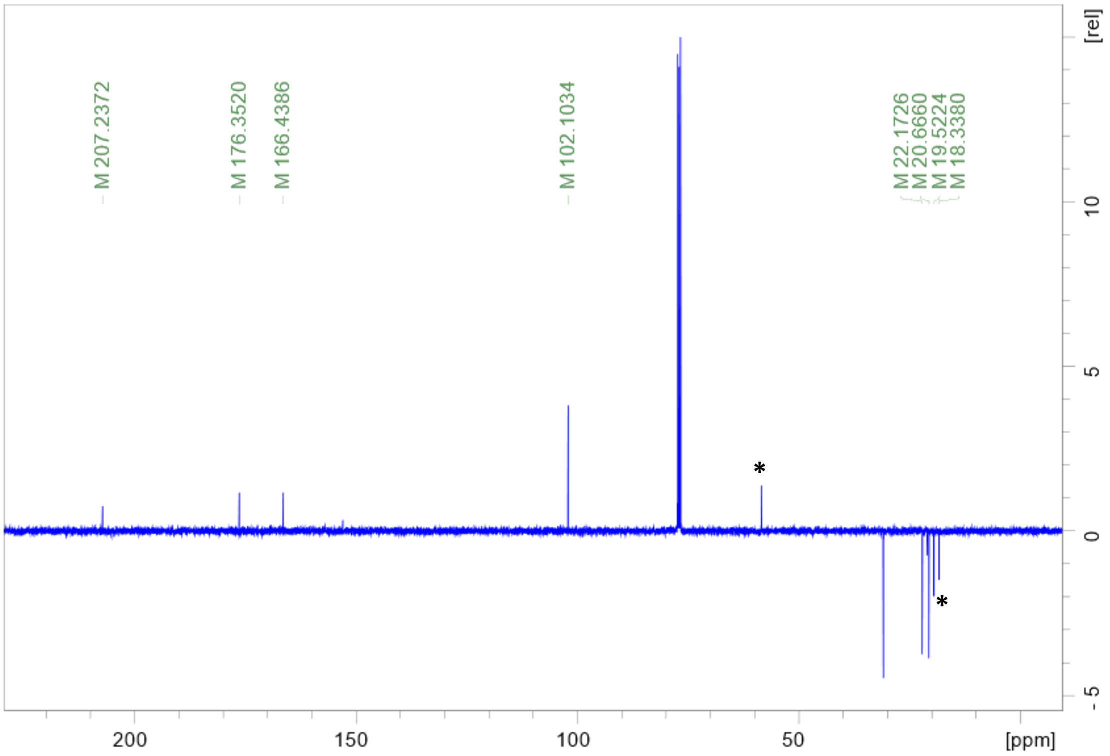

Figure S17. ^{13}C NMR spectrum of the volatiles mixture collected from the reaction of AA and iPAc. The * indicates the residual ethanol used to clean the NMR tubes. Solvent: $CDCl_3$.

Differential scanning calorimetry (DSC) curves of the glycerol/sorbitol+CLM mixtures

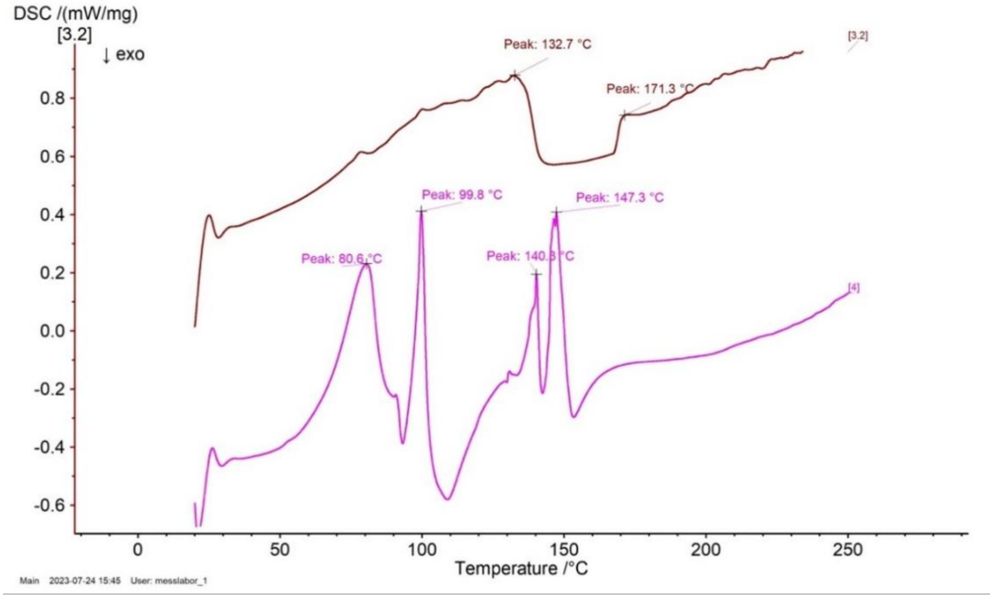

Figure S18. DSC thermograms of a mixture of glycerol and CLM (top, brown) and sorbitol and CLM (bottom, pink), mixed at room temperature and heated up to 250 °C to analyse the curing behaviour.

The formation of volatiles and the mass balance during the pre-polymer synthesis and during the materials curing

The formation of volatile and the mass balance of the procedure was investigated for all the samples. In addition, for S_1 and G_1 the by-products were characterized by GC-MS and NMR. to resemble the outcomes of the reaction for glycerol and sorbitol, respectively. The yields of volatiles obtained during the pre-polymer synthesis (V1), during the materials curing (V2) and of the materials (M) are reported in Table S3. The V1 fraction were simply isolated by distillation of the products using the apparatus of Figure S18.

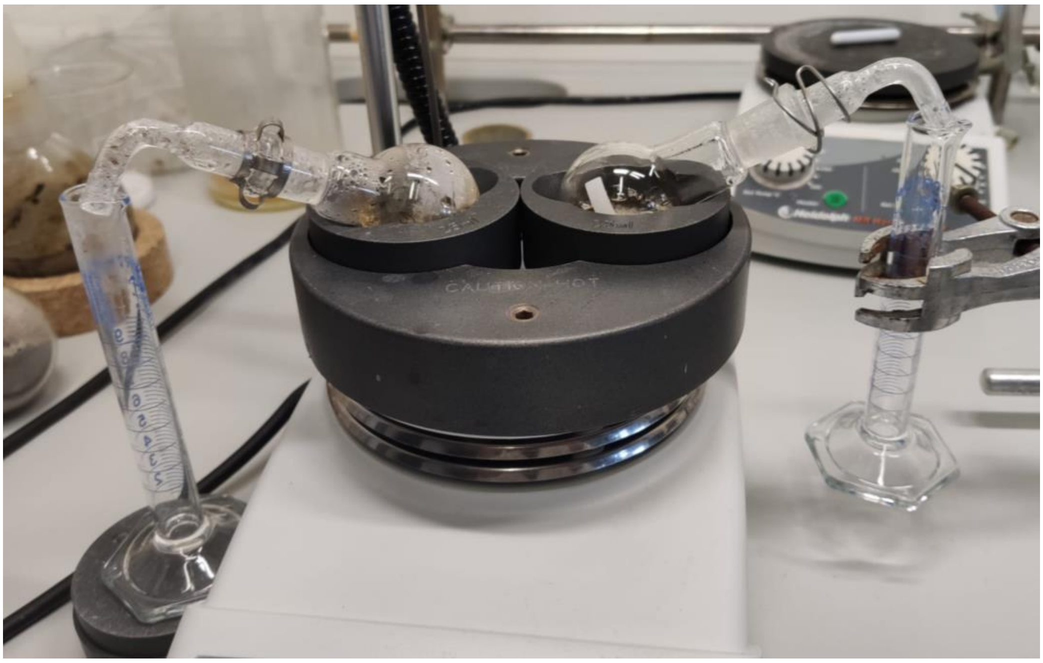

Figure S19. Apparatus used to isolate the volatiles of fractions V1 for S_1 and G_1.

The V2 fractions were isolated by washing the flasks of Figure S18 after the pre-polymer synthesis with acetone (100 mL each). Then, the V2 fractions were isolated by rotary evaporation (T = 40 °C; p = 10 mbar).

Table S3. The formation of volatiles during the pre-polymer preparation and during the material curing and the mass balance.

Entry	Sample	Glyc:Sorb:CLM (weight ratio)	Yield V1 (wt%)[a]	Yield V2 (wt%)[b]	Yield M (wt%)[c]	Mass balance (wt%)
1	G_1	1:0:1	12	3	85	>99
2	$G_{0.5}$	0.5:0:1	8	2	89	>99
3	S_1	0:1:1	11	3	86	>99
4	$S_{0.5}$	0:0.5:1	8	5	87	>99
5	$GS_{0.5}$	0.5:0.5:1	12	3	86	>99
6	$GS_{0.25}$	0.25:0.25:1	13	3	85	>99
7	$G_{0.2}S_{0.8}$	0.2:0.8:1	15	3	83	>99
8	$G_{0.8}S_{0.2}$	0.8:0.2:1	15	4	82	>99

[a]Yield of volatiles during the pre-polymer synthesis. [b]Yield of volatiles during the material curing. [c]Yield of the thermoset materials.

As mentioned, the chemical composition of the volatiles fraction V1 for S_1 and G_1 has been investigated by NMR. The ^1H NMR spectra of V1-G_1 shows that AcOH and acrolein are formed during the pre-polymer synthesis in a 7.4:1 mol:mol ratio (Figure S19). The fraction V1 for S_1 was solely composed by AcOH as confirmed by the ^1H NMR spectrum (Figure S20).

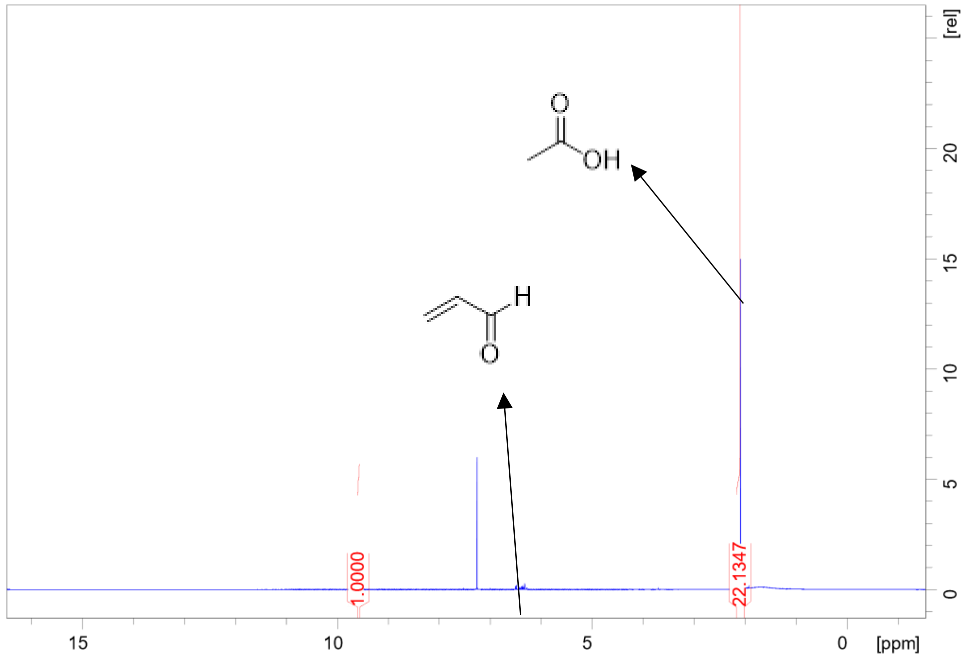

Figure S20. ^1H NMR spectrum of the fraction V1 for samples G_1. (400 MHz, 298 K, $CDCL_3$).

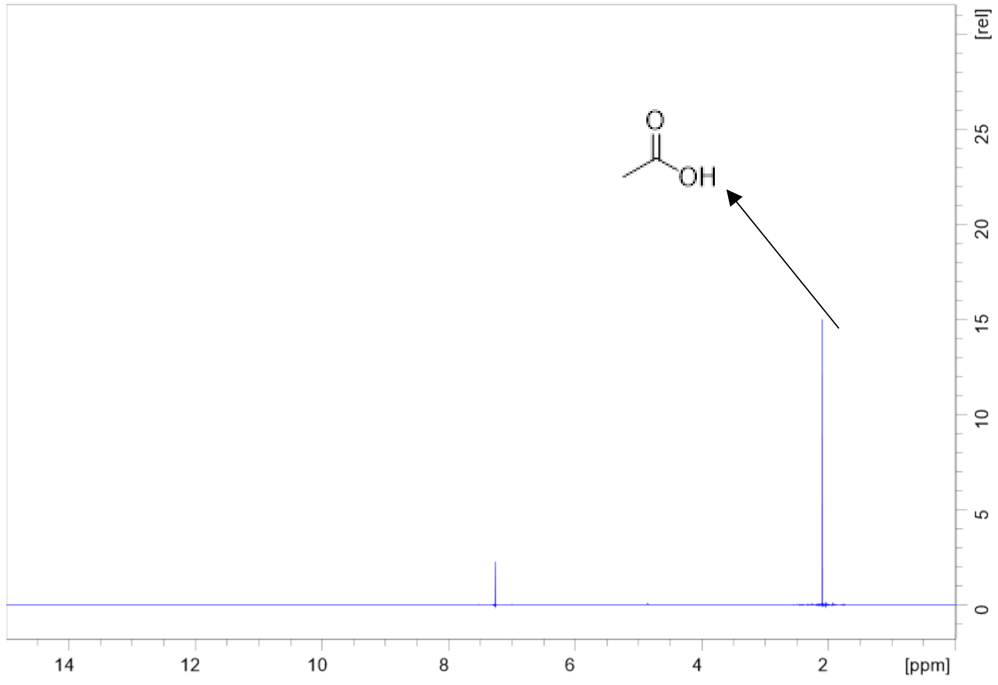

Figure S21. ^1H NMR spectrum of the fraction V1 for samples S_1. (400 MHz, 298 K, $CDCL_3$).

Due to the complexity of the V2 fractions GC-MS analysis were requested to evaluate their composition. The GC-MS chromatogram of the fraction V2 for sample G_1 (V2-G_1) is reported in Figure S21. The observed and characterized products are solketal, solketal acetate, mono-/di-acetates of glycerol and (1,4-dioxane-2,5-diyl)dimethanol. Solketal and solketal acetate are formed during the acetone washing for the isolation of the V2 fractions (see procedure above). They most likely suggest the presence of glycerol and glycerol monoacetate as volatile products in the V2-G_1 fraction.

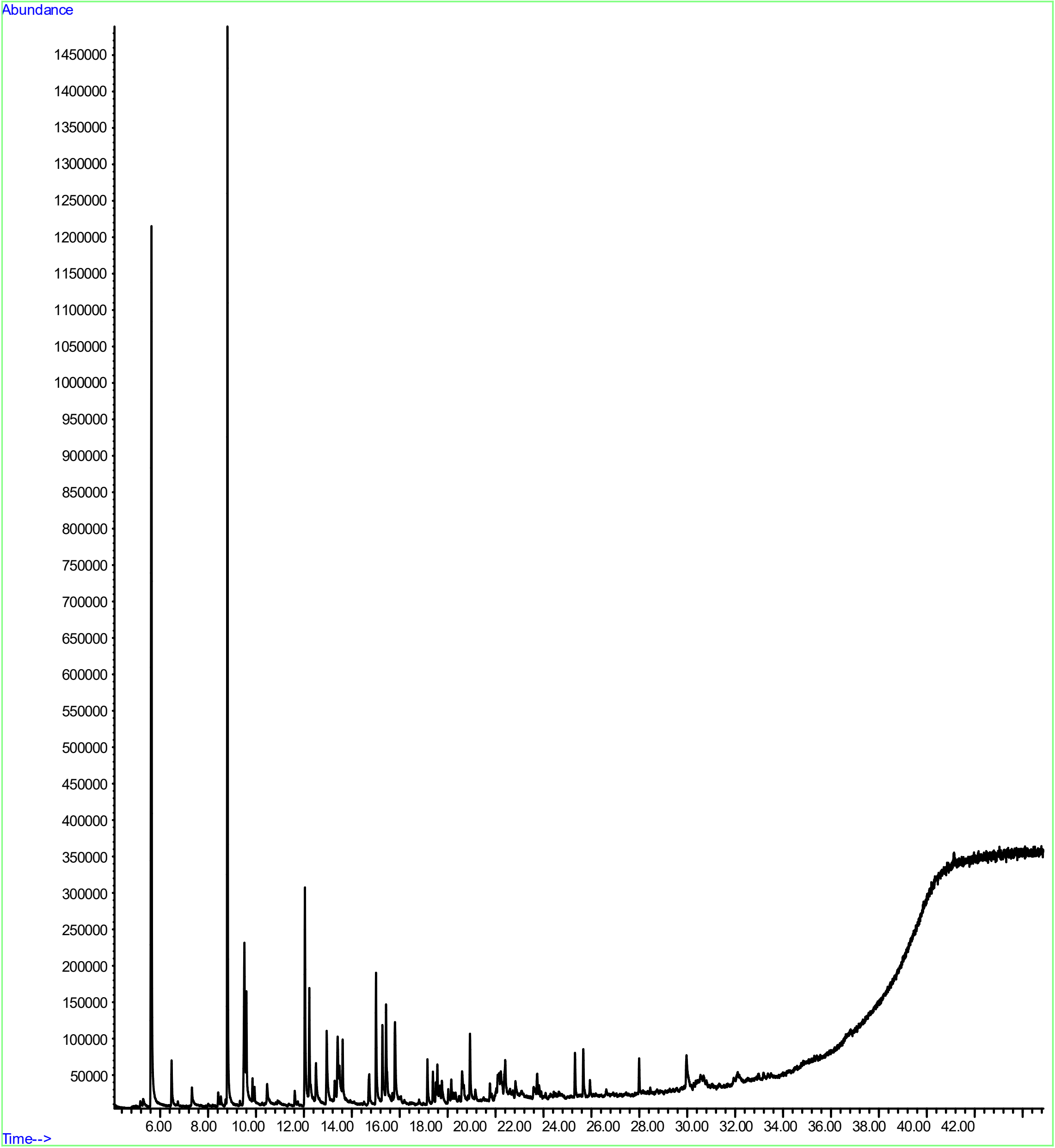

Figure S22. Chromatogram of the V2 fraction for sample G_1.

Table S4. Volatile products detected by GC-MS analysis of V2-G_1 fraction.

Compound	Retention time (min)	Structure	Amount (% by GC)
Solketal	5.62	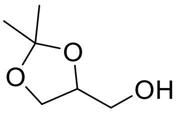	18
Solketal acetate	8.81	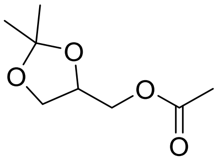	16
Glycerol monoacetates	9.51 and 9.59	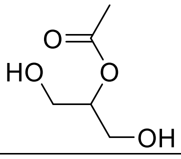 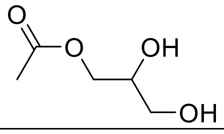	8
Glycerol diacetates	12.05 and 12.24	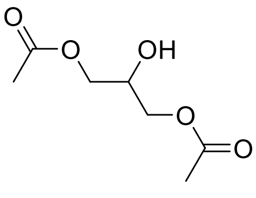 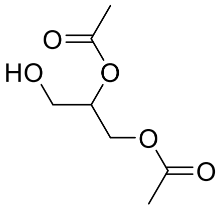	9
(1,4-dioxane-2,5-diyl)dimethanol	12.50	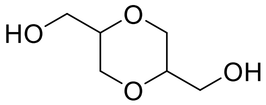	7
Others	-	-	42

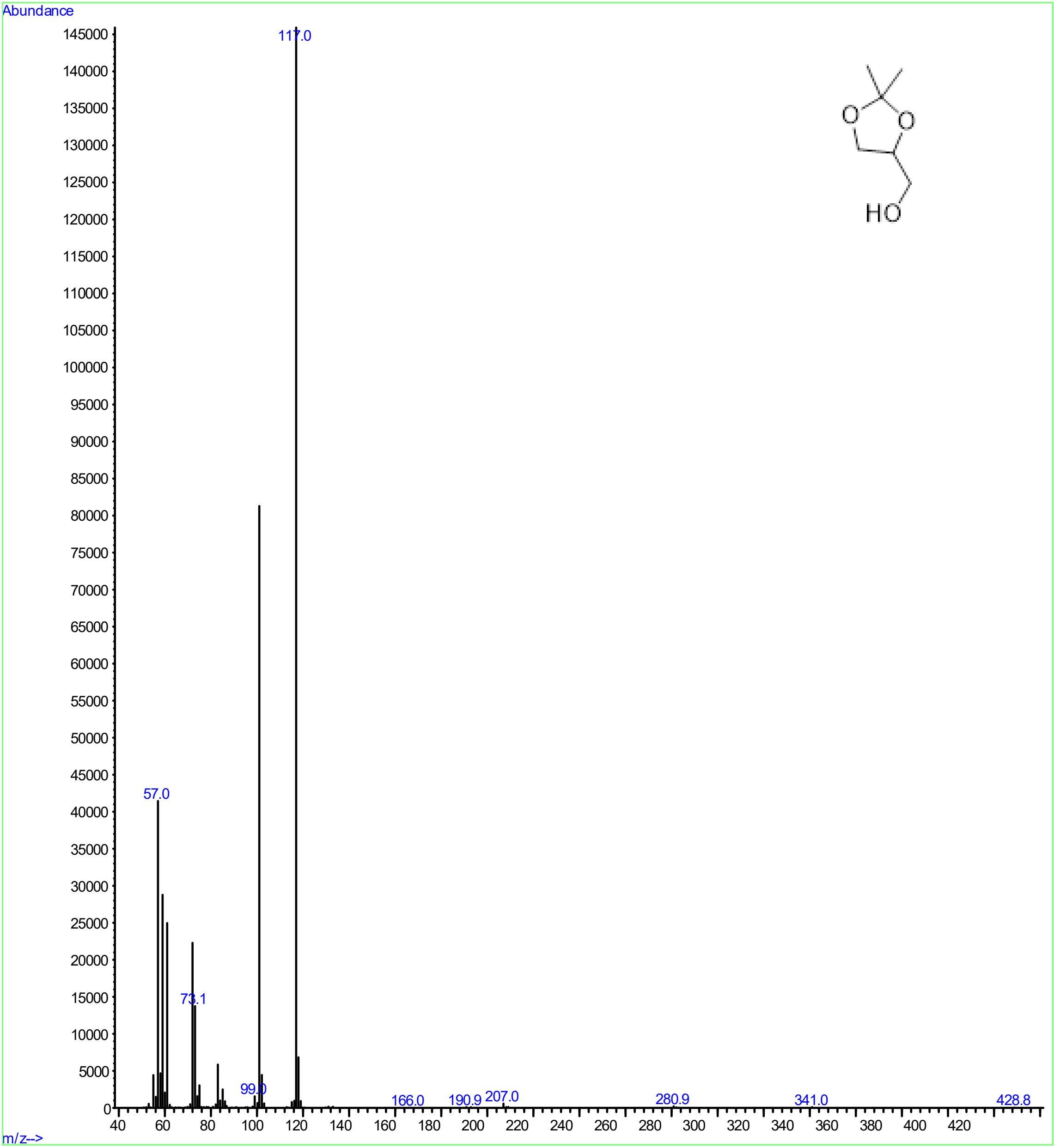

Figure S23. MS spectrum of solketal (EI, 70 eV).

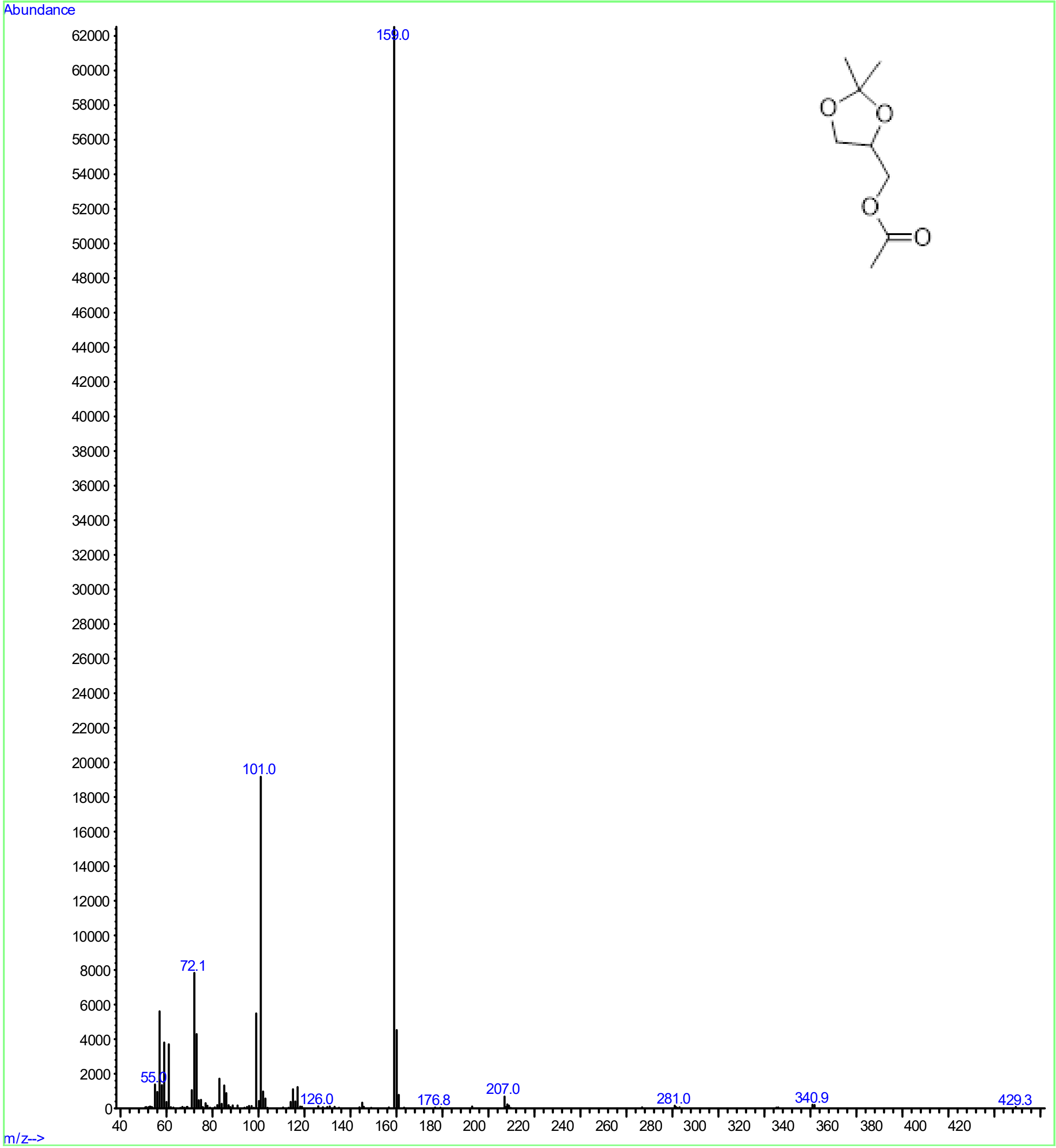

Figure S24. MS spectrum of solketal acetate (EI, 70 eV).

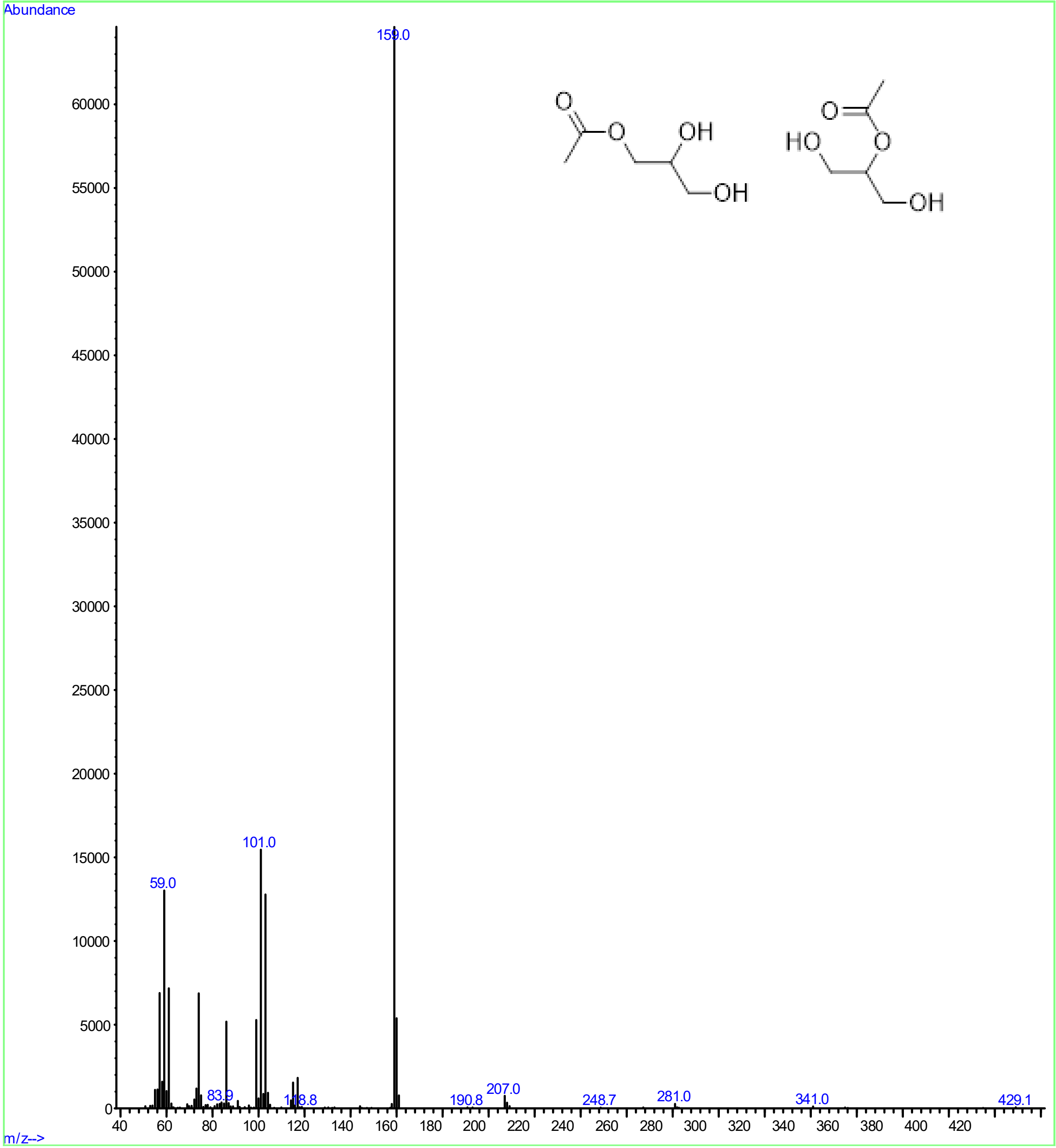

Figure S25. MS spectrum of glycerol monoacetates (EI, 70 eV).

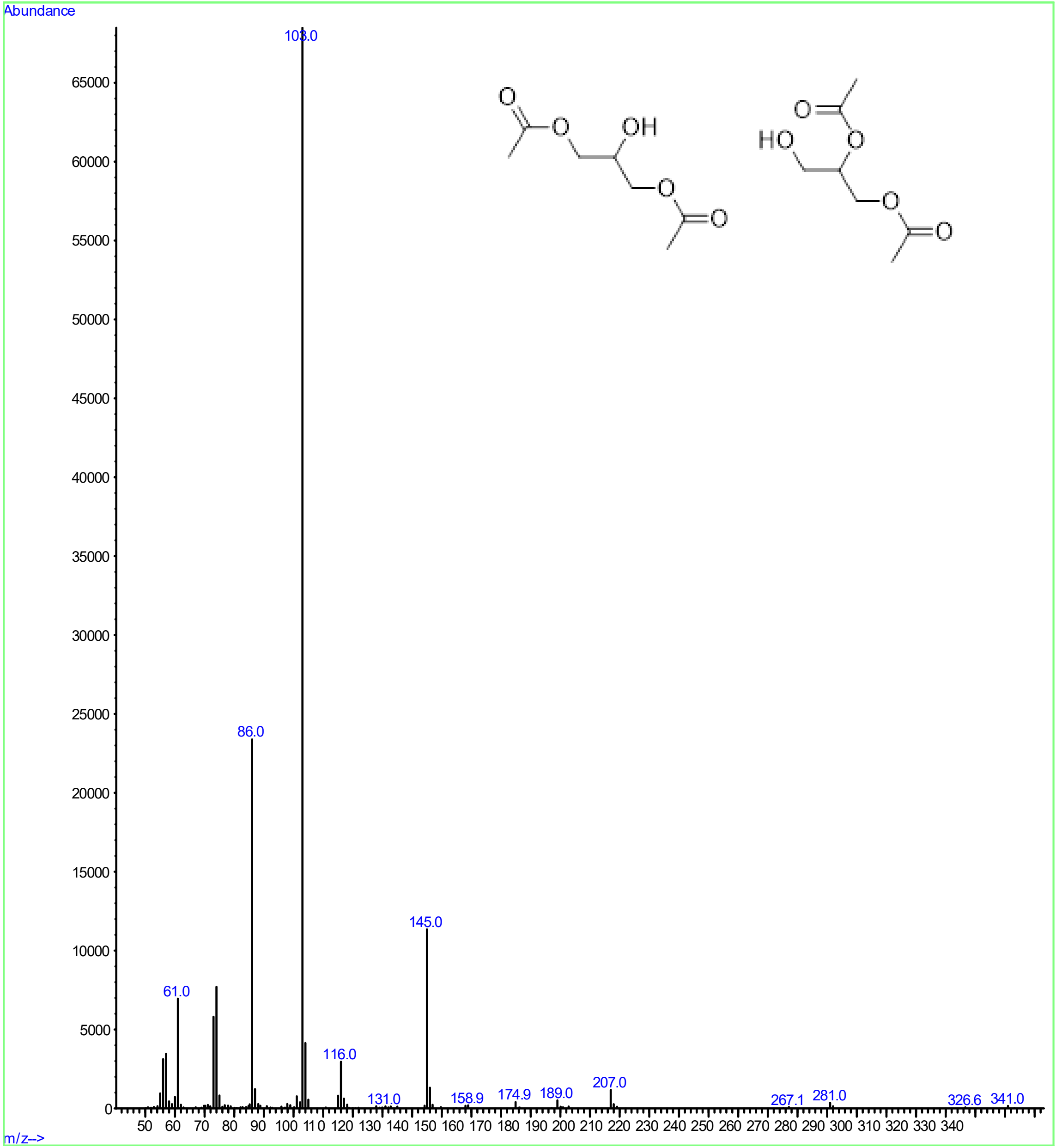

Figure S26. MS spectrum of glycerol diacetates (EI, 70 eV).

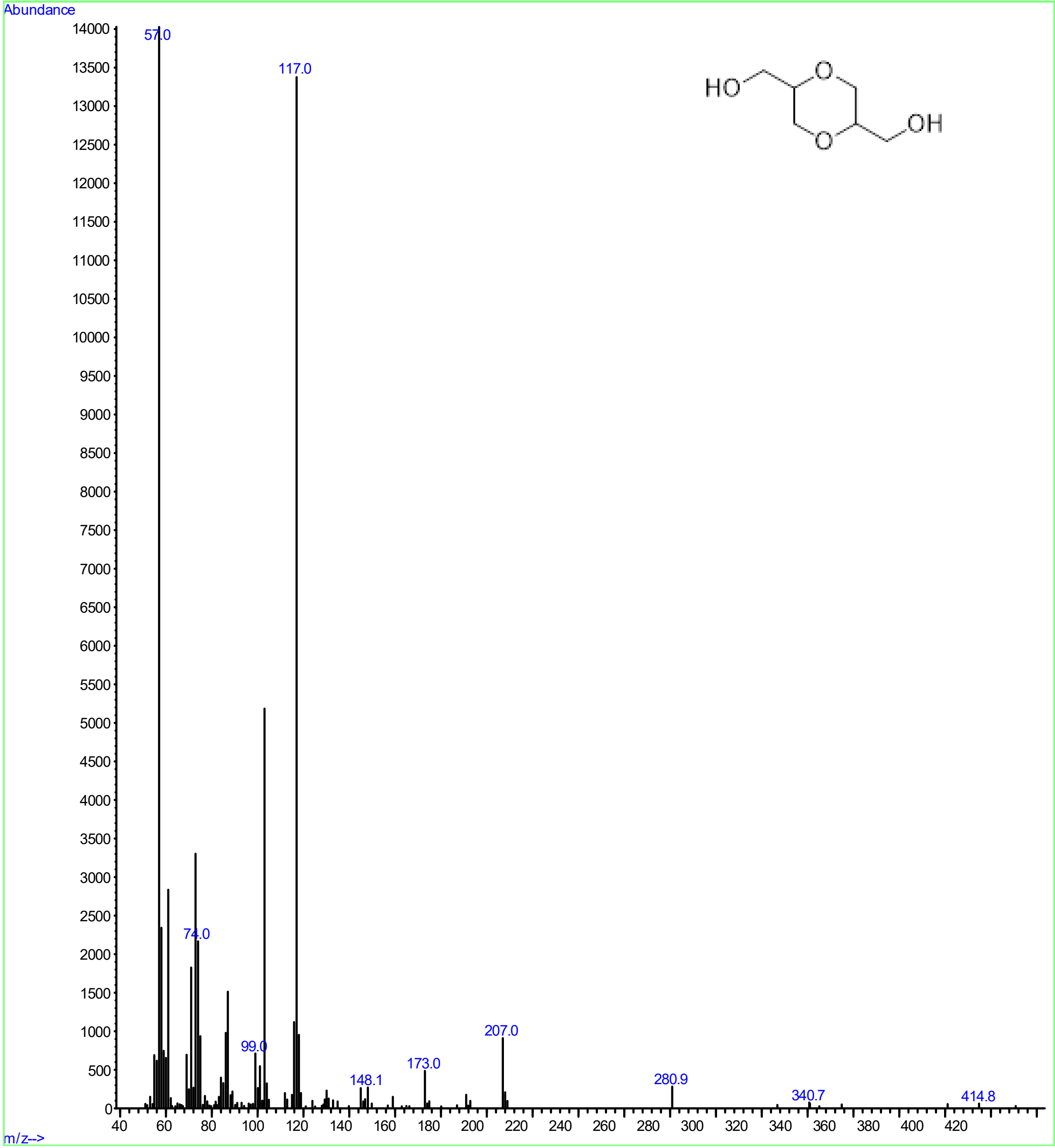

Figure S27. MS spectrum of (1,4-dioxane-2,5-diyl)dimethanol (EI, 70 eV).

The chromatogram of the V2 fraction for sample S_1 (V2-S_1) is reported in Figure S27. The complexity of the mixture allowed for the characterization of two products, namely isosorbide and diacetyl isosorbide (Table S5).

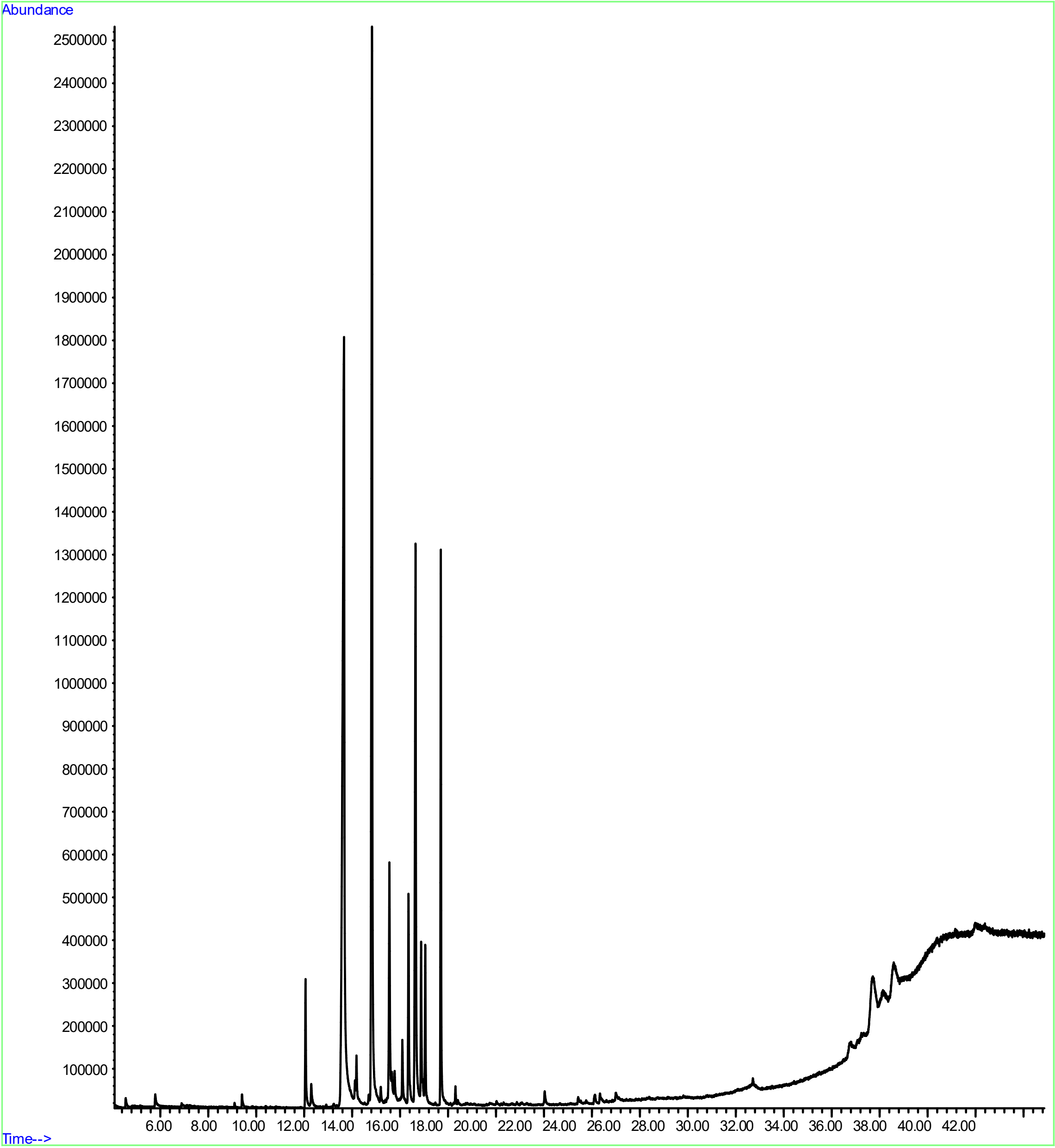

Figure S28. Chromatogram of the V2 fraction for sample S_1.

Table S5. Volatile products detected by GC-MS analysis of V2-S_1 fraction.

Compound	Retention time (min)	Structure	Amount (% by GC)
Isosorbide	13.57	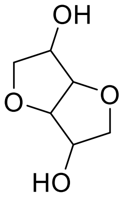	33
Diacetyl isosorbide	17.71	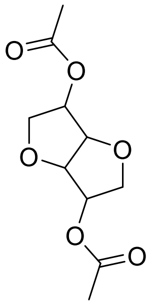	8
Others	-	-	59

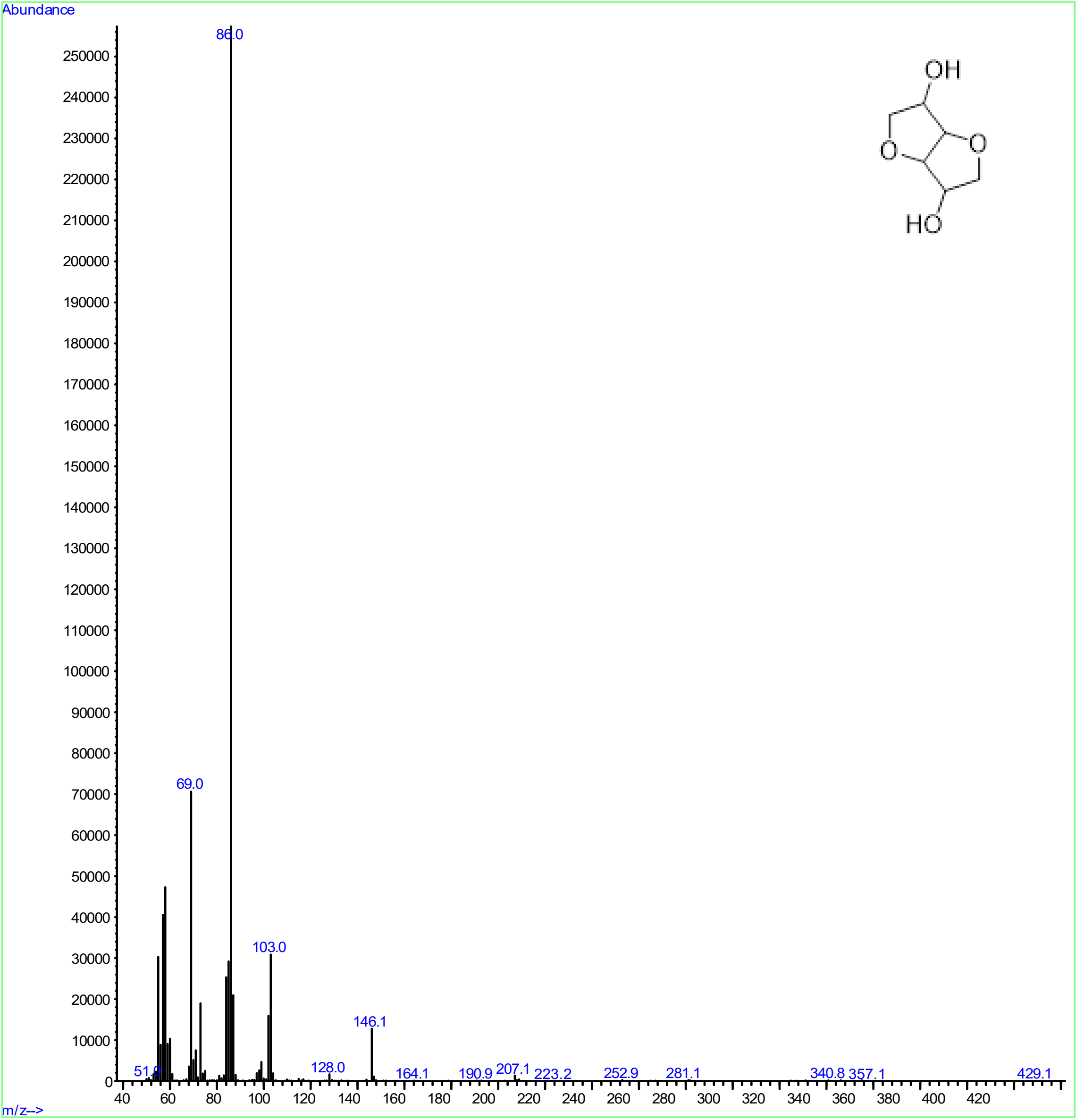

Figure S29. MS spectrum of isosorbide (EI, 70 eV).

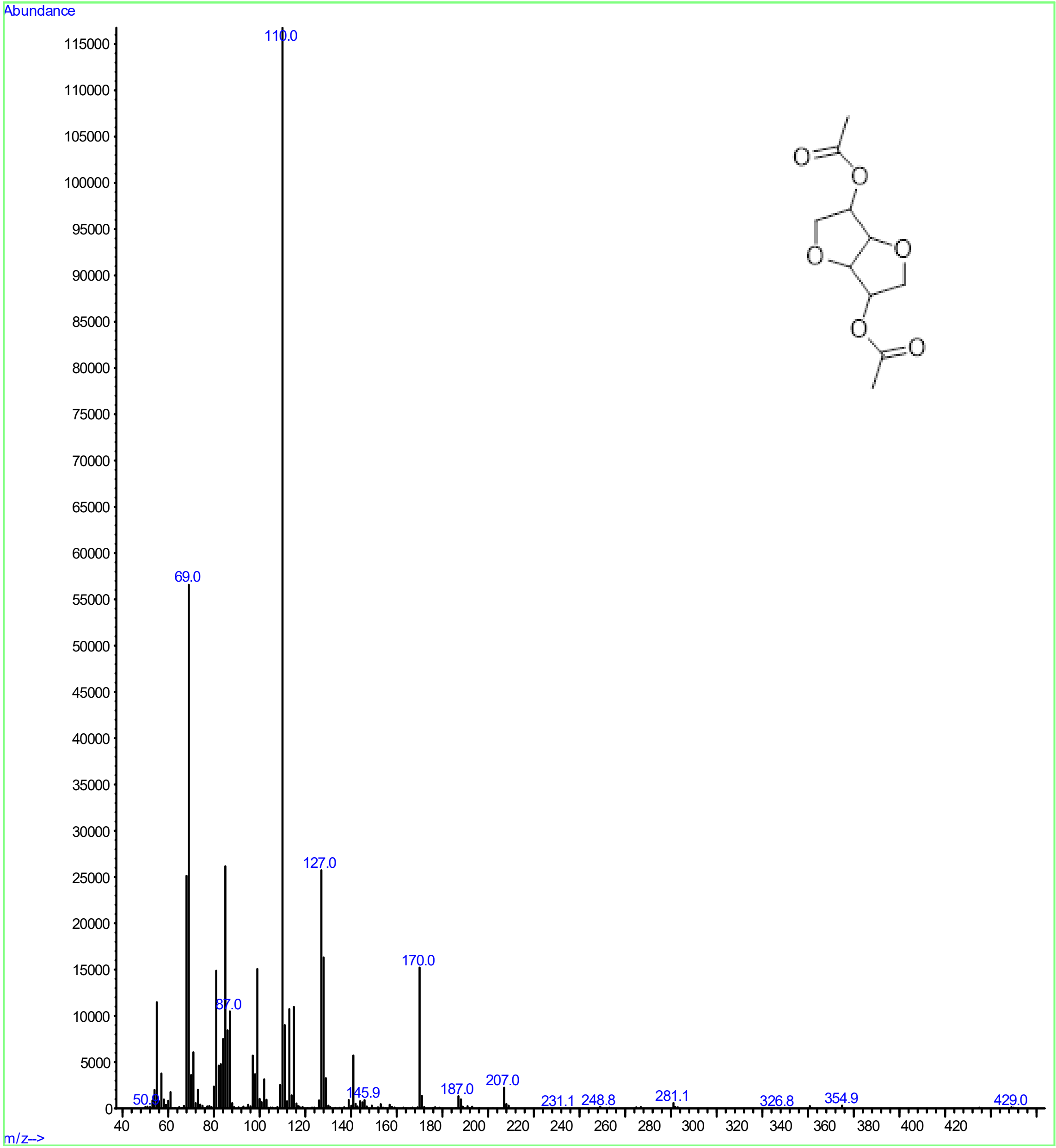

Figure S30. MS spectrum of diacetyl isosorbide (EI, 70 eV).

The synthesis of elastomeric foams

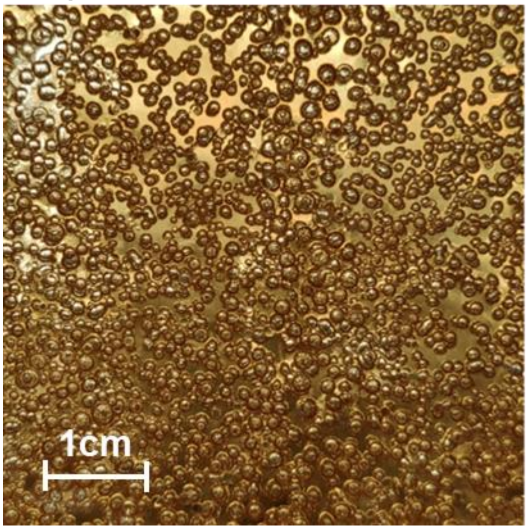

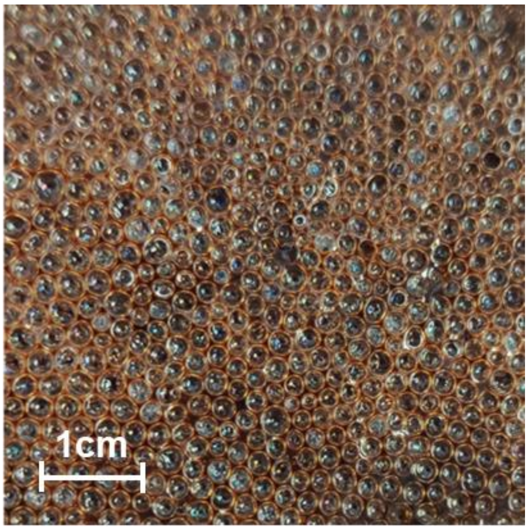

Figure S31. Elastomeric foams produced when the pre-polymer synthesis was shortened to 30 min before molding. Left: G_1-foam. Right: S_1 foam.

The synthesis/characterization of these polymers were beyond the scope of the present work, but from a macroscopic standpoint, their appearance, flexibility and stretching looked more than promising for applications in low-density plastic packaging which will be investigated in a follow-up work.

FT-IR spectra

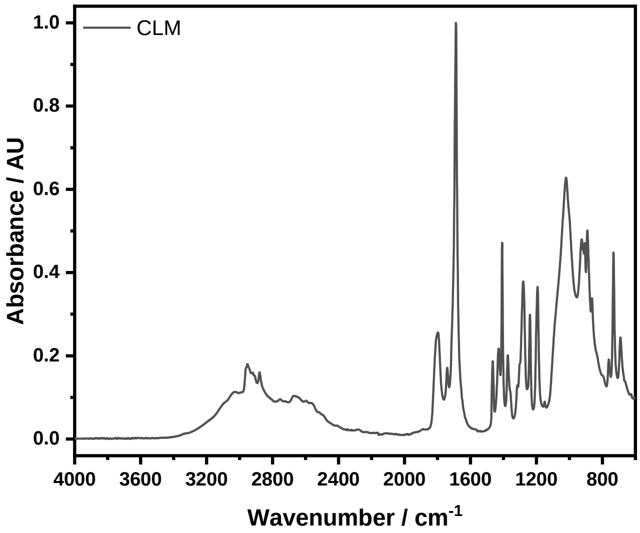

Figure S32. Normalized Fourier-transform infrared spectra of the CLM starting material.

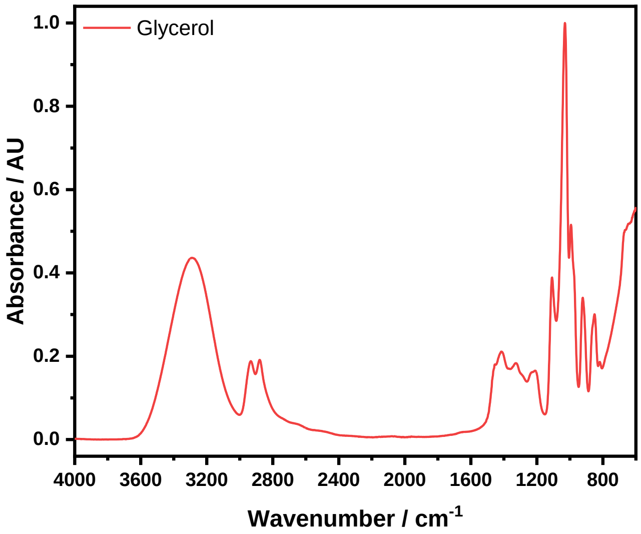

Figure S33. Normalized Fourier-transform infrared spectra of the glycerol starting material.

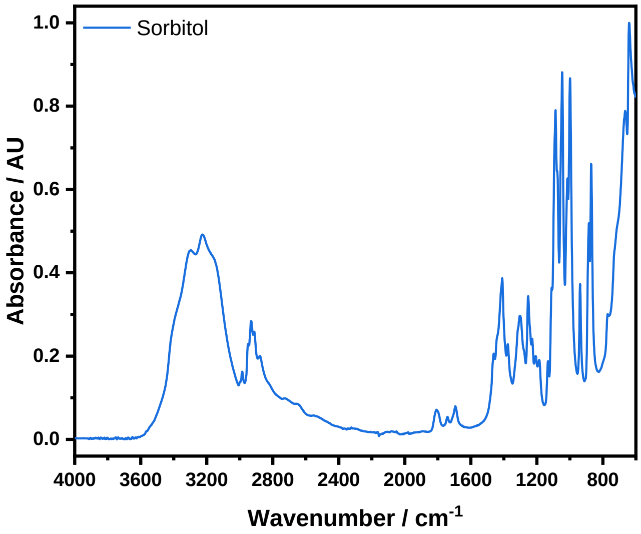

Figure S34. Normalized Fourier-transform infrared spectra of the sorbitol starting material.

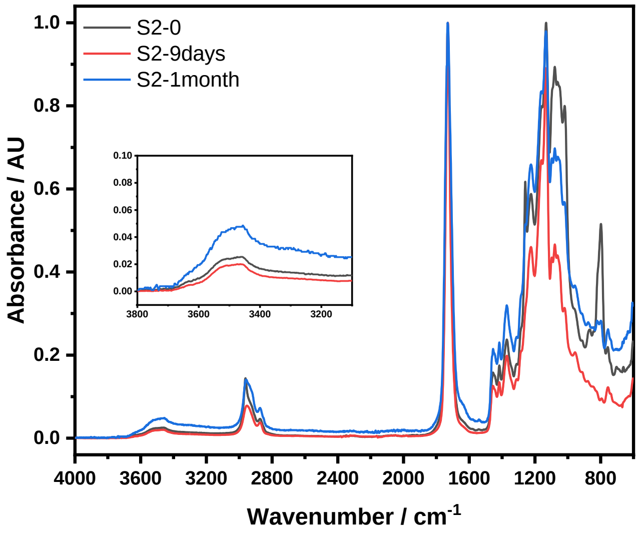

Figure S35. Normalized Fourier-transform infrared spectra of sample $G_{0.5}$ at three different time intervals: after preparation, after 9 days of storage, and after 1 month of storage.

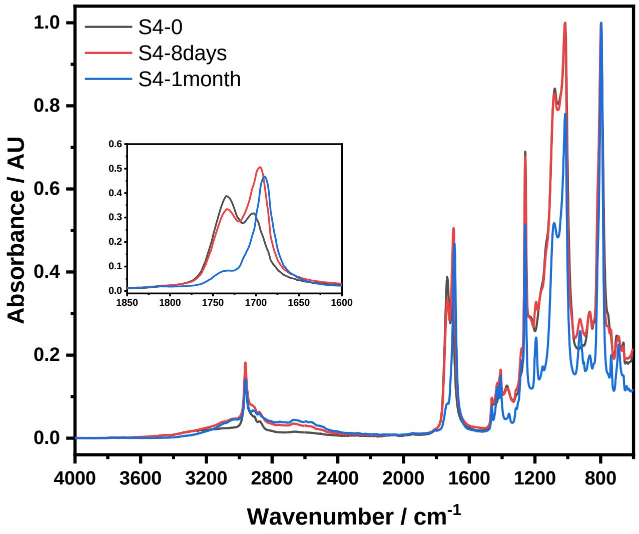

Figure S36. Normalized Fourier-transform infrared spectra of sample $S_{0.5}$ at three different time intervals: after preparation, after 8 days of storage, and after 1 month of storage.

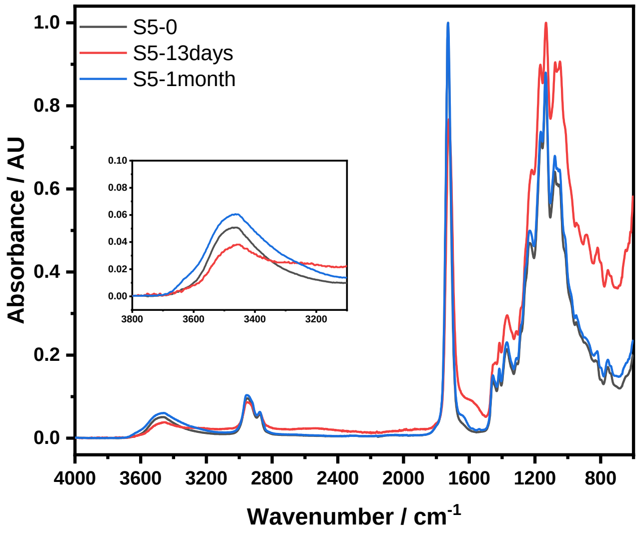

Figure S37. Normalized Fourier-transform infrared spectra of sample $GS_{0.5}$ at three different time intervals: after preparation, after 13 days of storage, and after 1 month of storage.

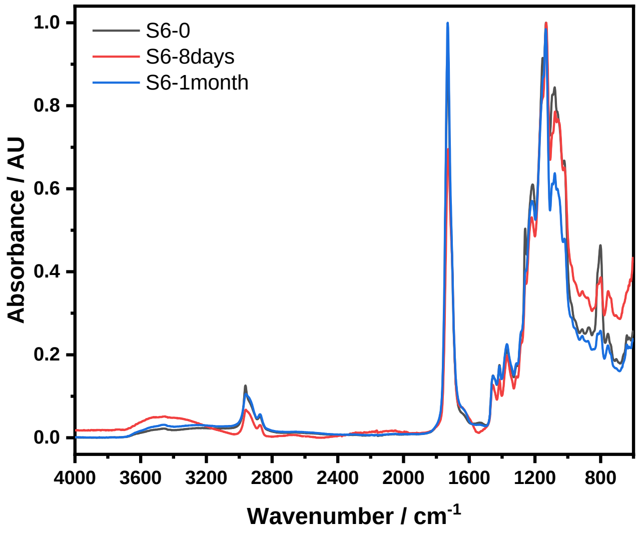

Figure S38. Normalized Fourier-transform infrared spectra of sample $GS_{0.25}$ at three different time intervals: after preparation, after 8 days of storage, and after 1 month of storage.

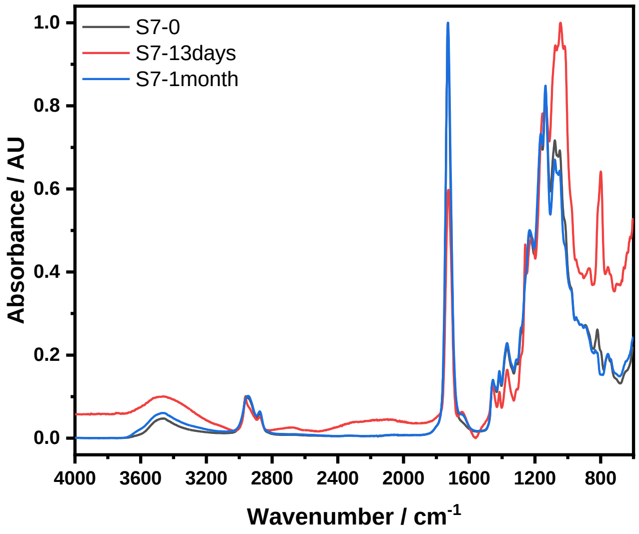

Figure S39. Normalized Fourier-transform infrared spectra of sample $G_{0.2}S_{0.8}$ at three different time intervals: after preparation, after 13 days of storage, and after 1 month of storage.

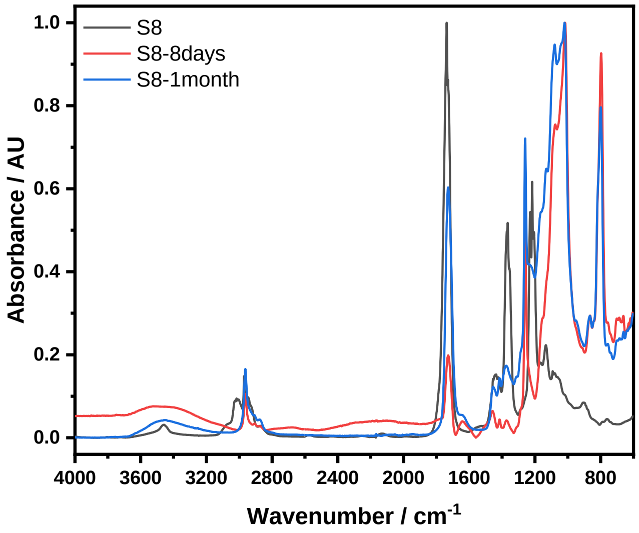

Figure S40. Normalized Fourier-transform infrared spectra of sample $G_{0.8}S_{0.2}$ at three different time intervals: after preparation, after 8 days of storage, and after 1 month of storage.

NMR characterization of the materials

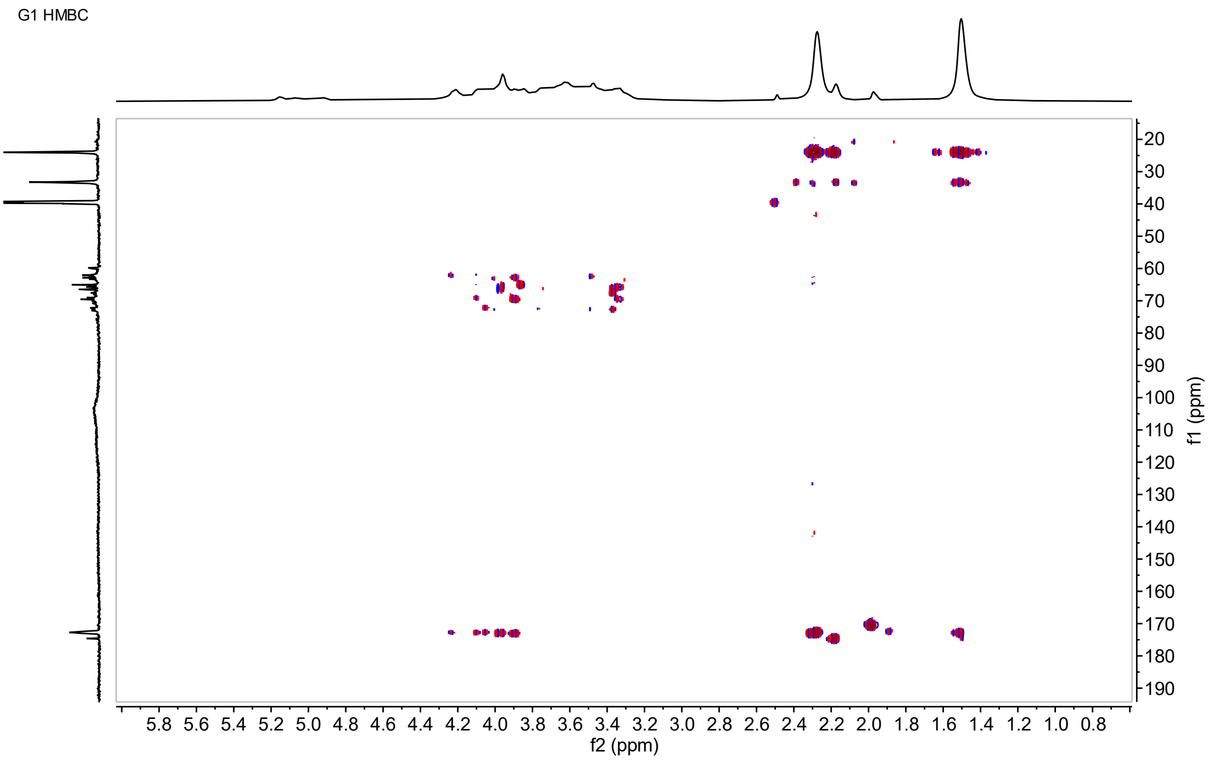

Figure S41. HR-MAS HMBC spectrum of swollen G_1. Solvent: DMSO-d_6.

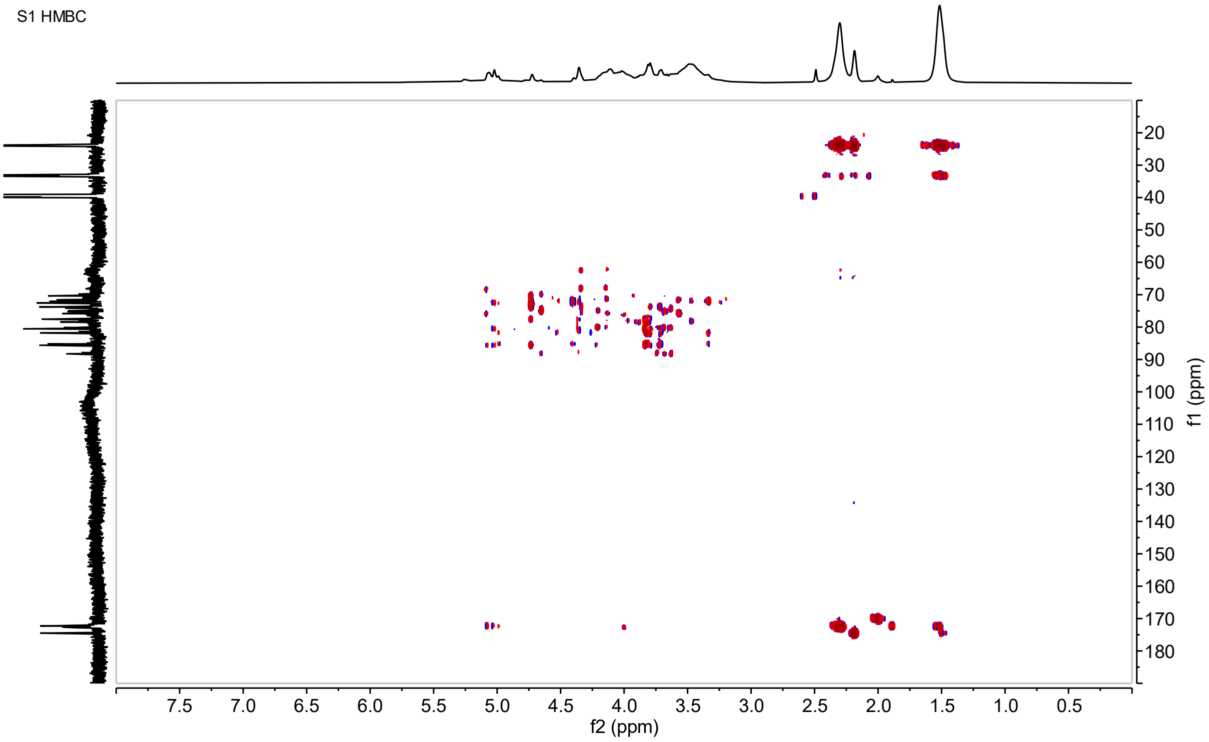

Figure S42. HR-MAS HMBC spectrum of swollen S_1. Solvent: DMSO-d_6.

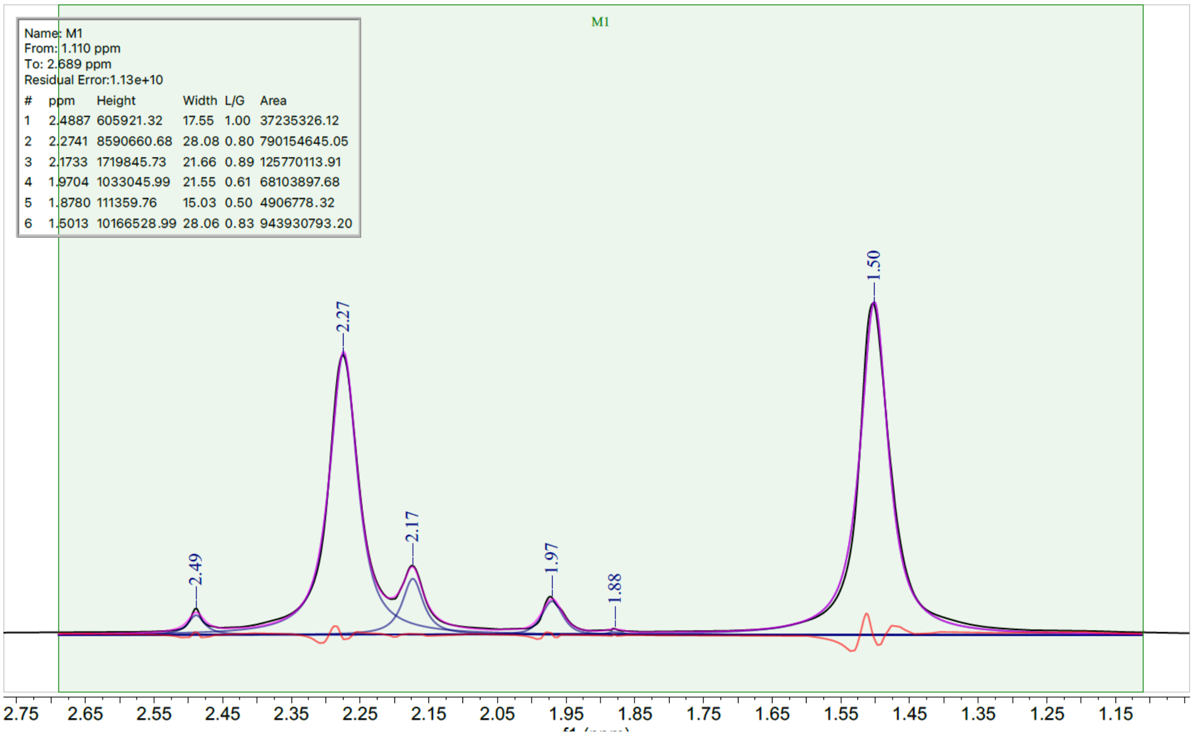

Figure S43. Aliphatic region of the ^{1}H NMR spectrum of G_1. Deconvolution of peaks are shown.

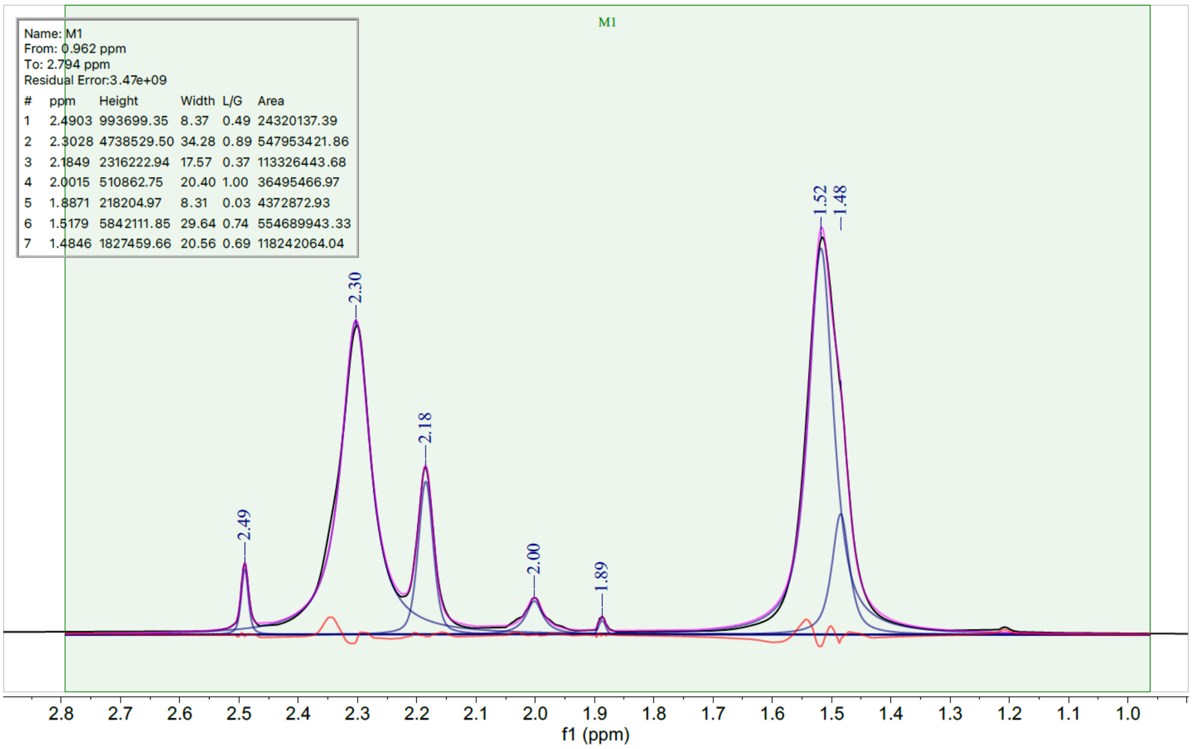

Figure S44. Aliphatic region of the ^{1}H NMR spectrum of S_1. Deconvolution of peaks are shown.

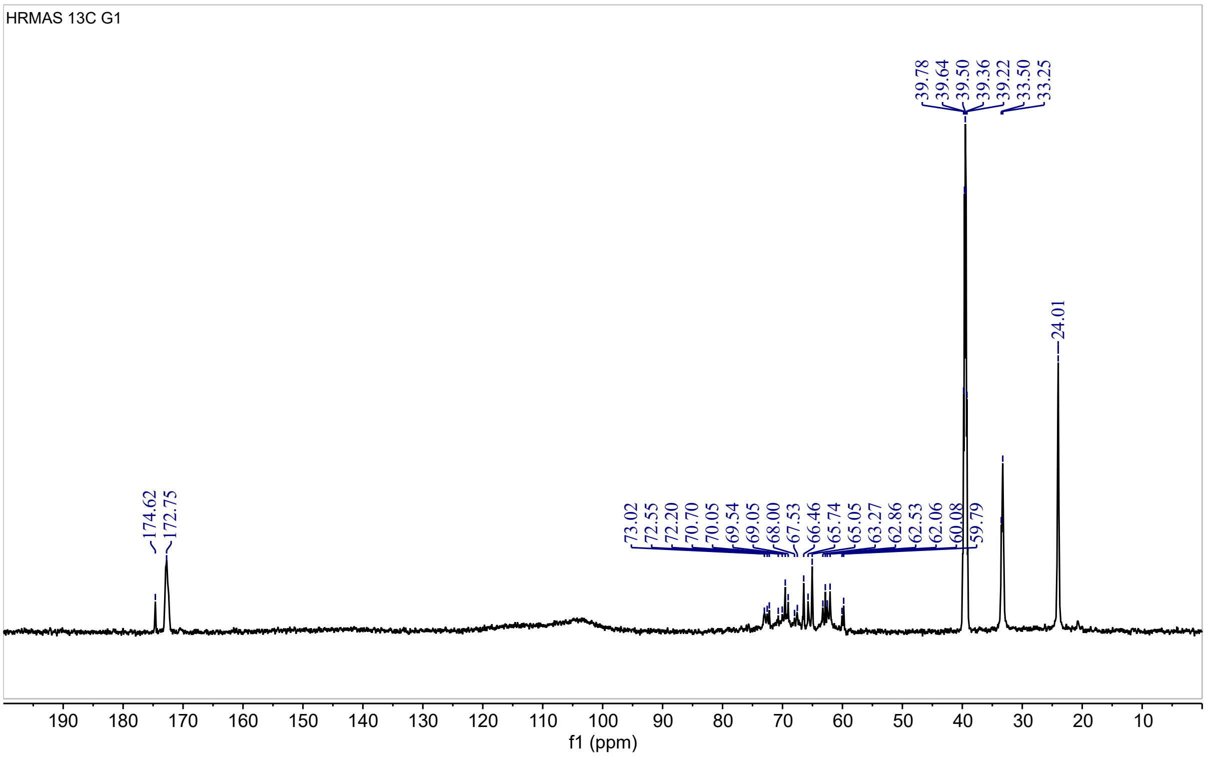

Figure S45. HR-MAS ^{13}C spectrum of swollen G_1. Solvent: DMSO-d_6.

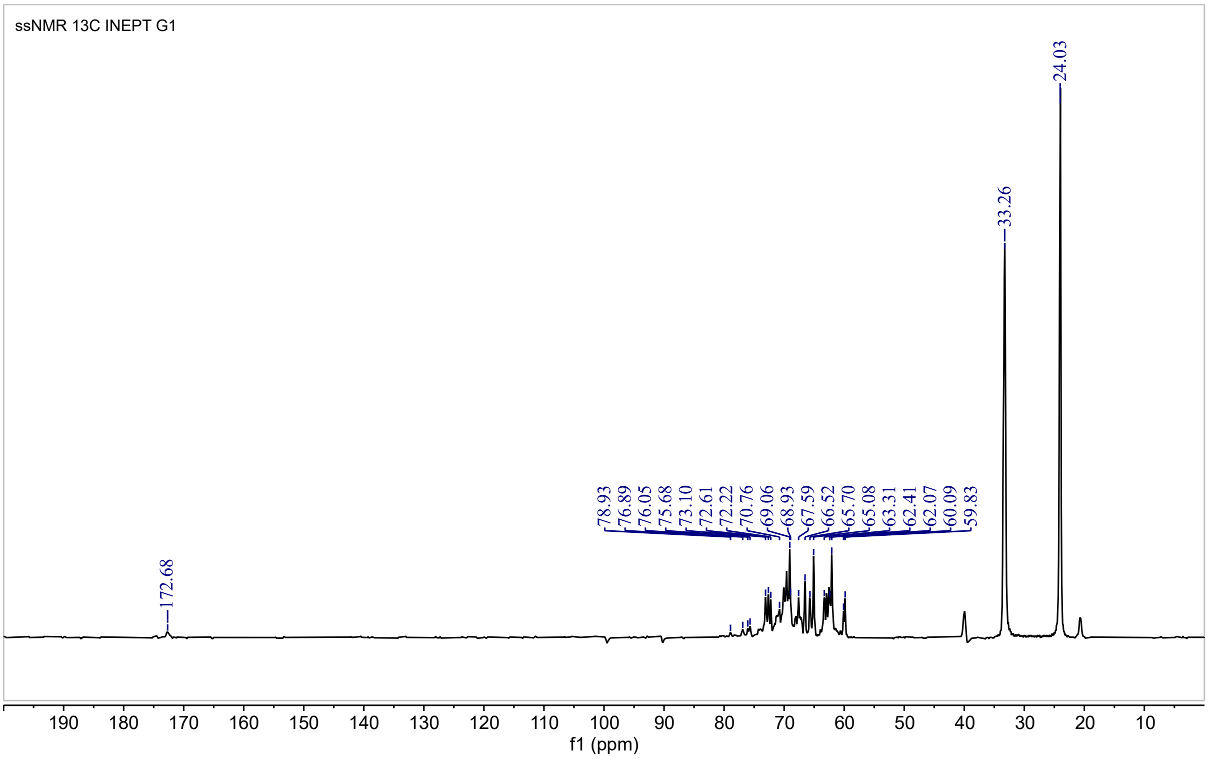

Figure S46. ssINEPT ^{13}C spectrum of G_1.

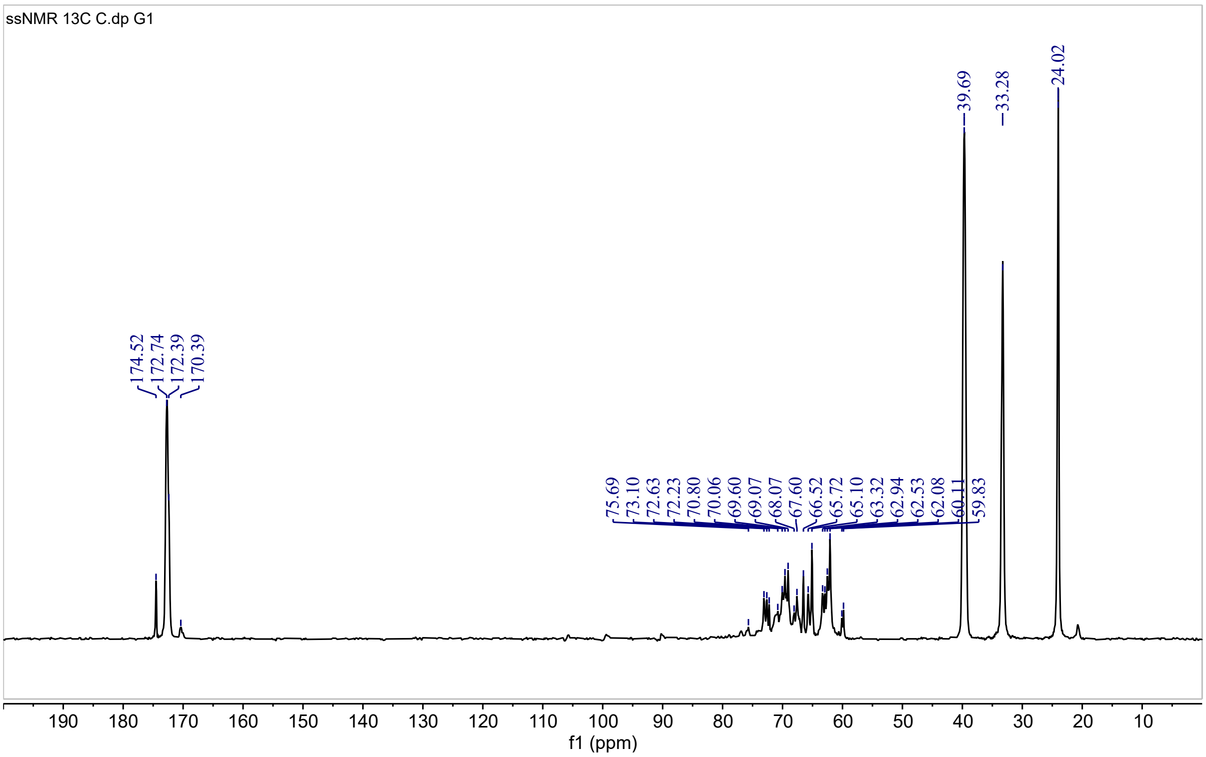

Figure S47. ssDP-MAS ^{13}C spectrum of G_1.

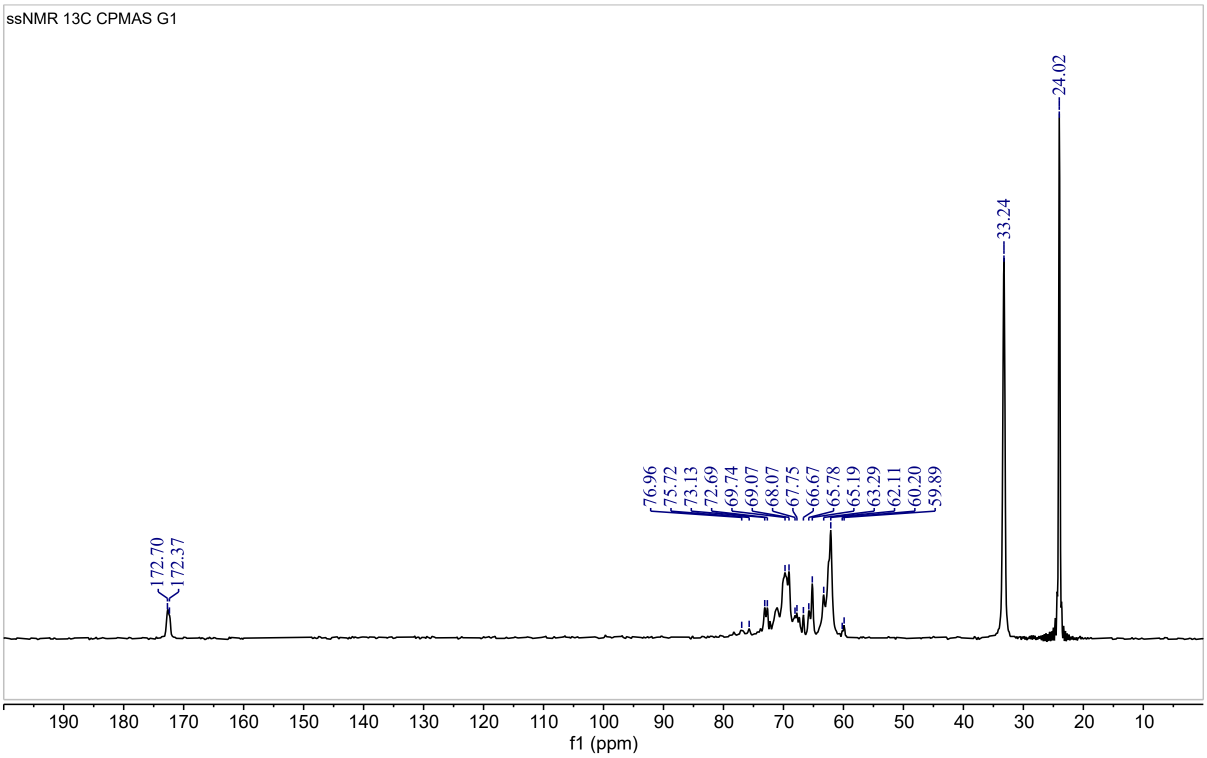

Figure S48. ssCP-MAS ^{13}C spectrum of G_1.

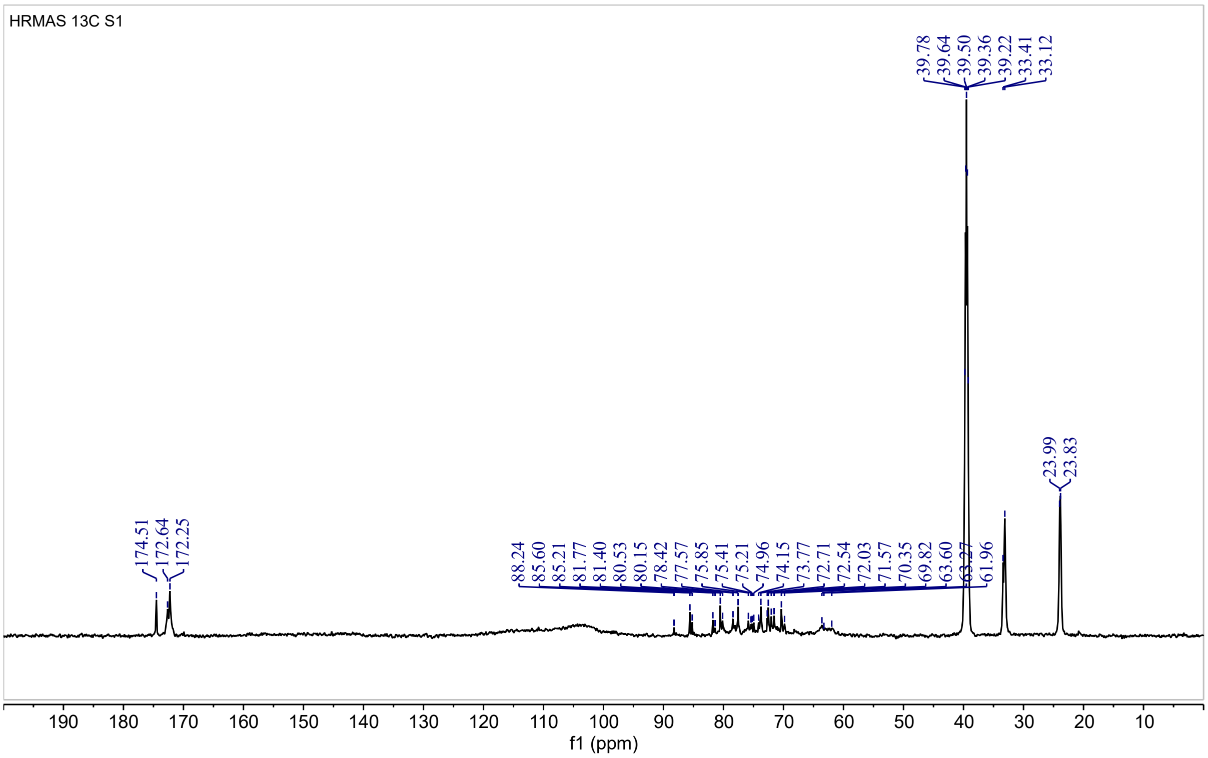

Figure S49. HR-MAS ^{13}C spectrum of swollen S_1. Solvent: DMSO-d_6.

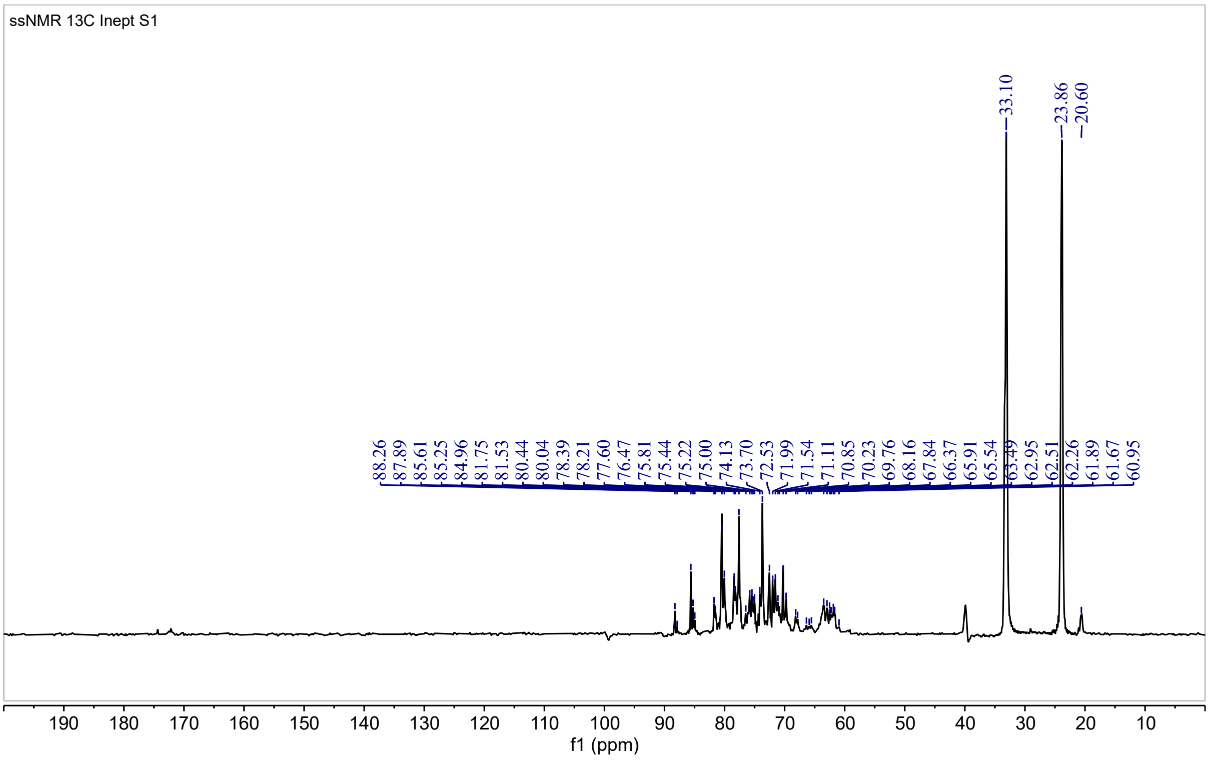

Figure S50. ssINEPT ^{13}C spectrum of S_1.

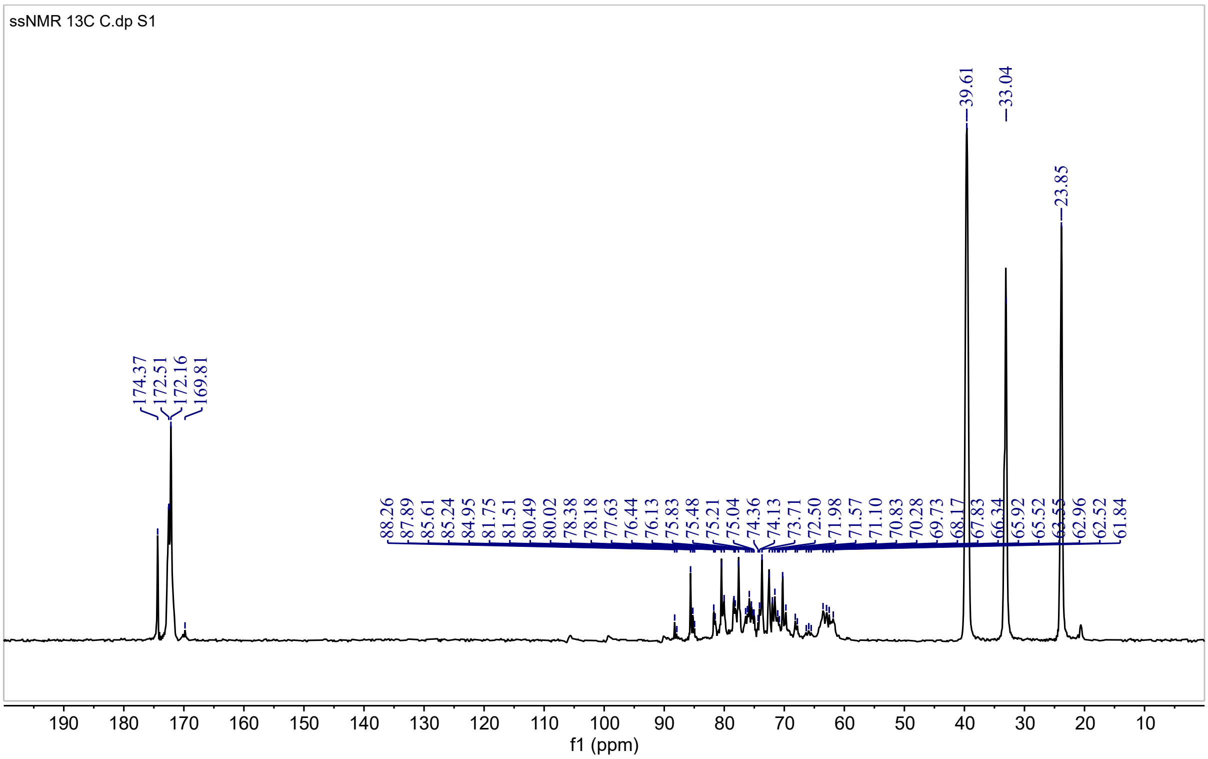

Figure S51. ssDP-MAS ^{13}C spectrum of S_1.

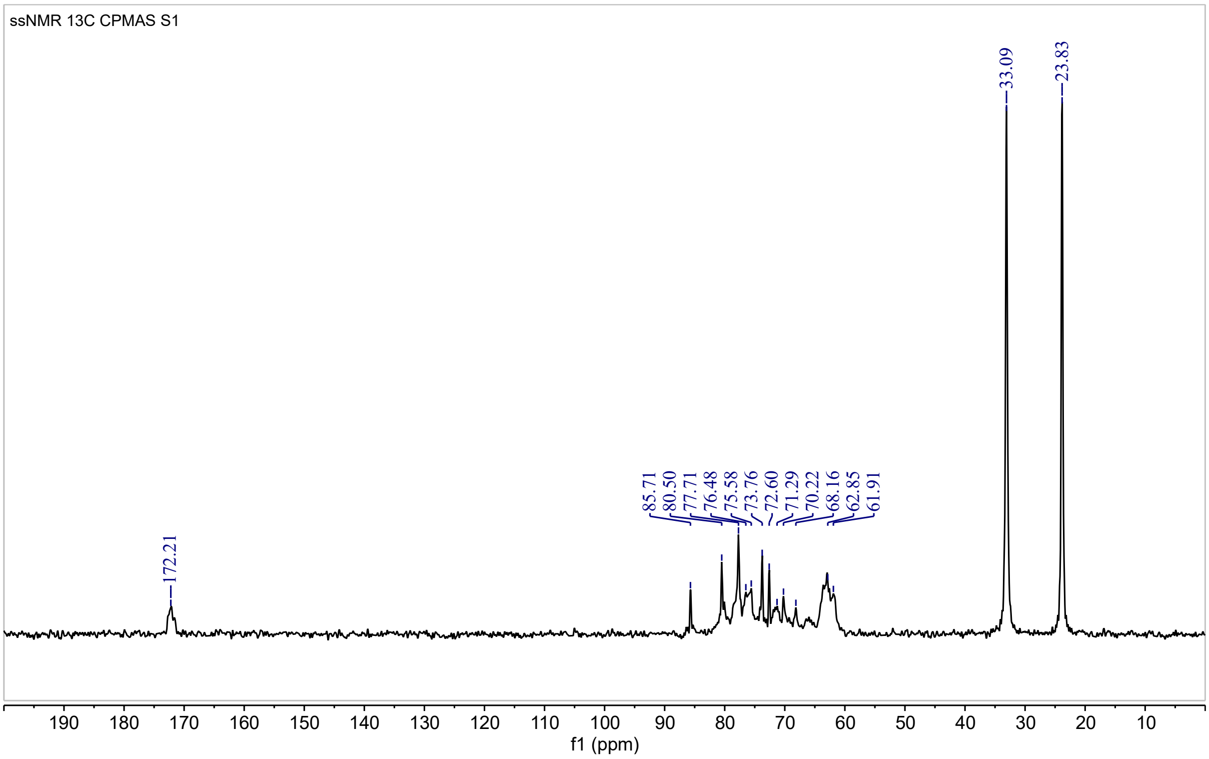

Figure S52. ssCP-MAS ^{13}C spectrum of S_1.

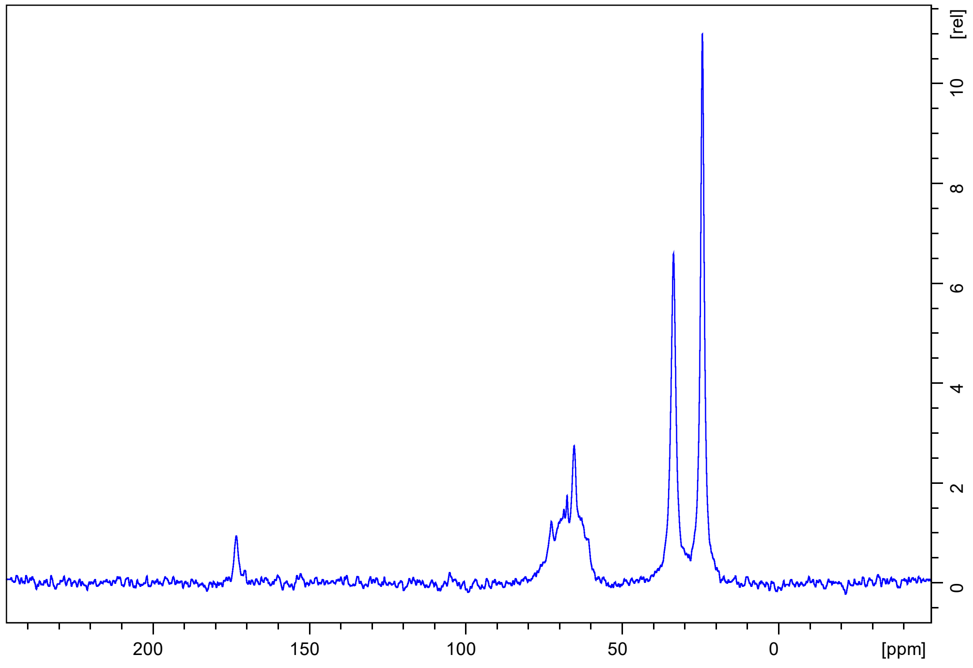

Figure S53. Solid state ^{13}C NMR spectrum of sample G_1. Conditions: glycerol:CLM = 1:1 (wt/wt).

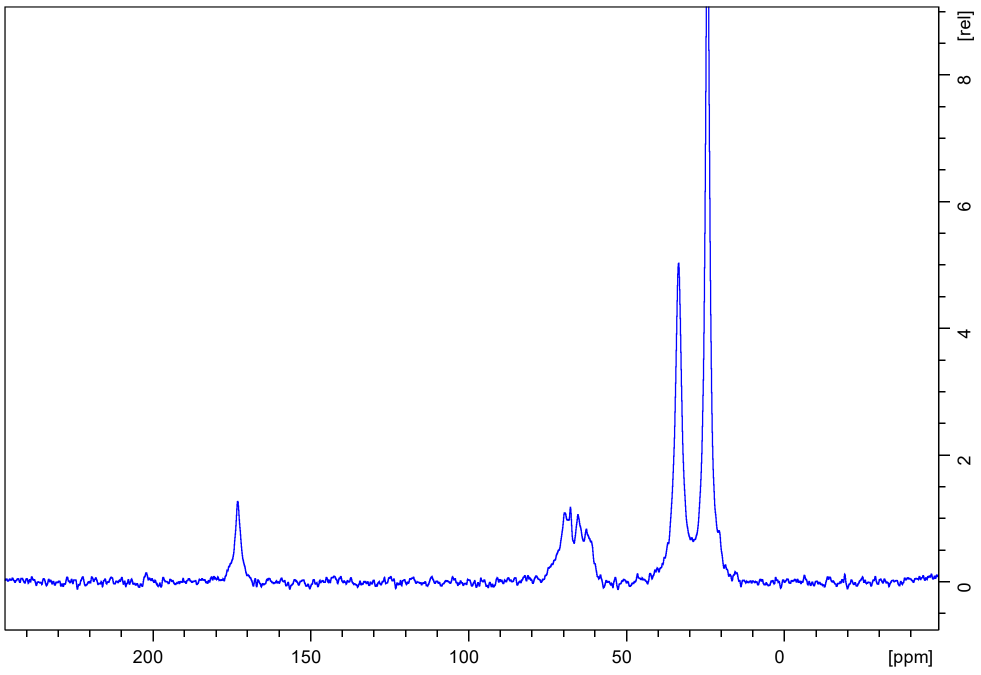

Figure S54. Solid state ^{13}C NMR spectrum of sample $G_{0.5}$. Conditions: glycerol:CLM = 0.5:1 (wt/wt).

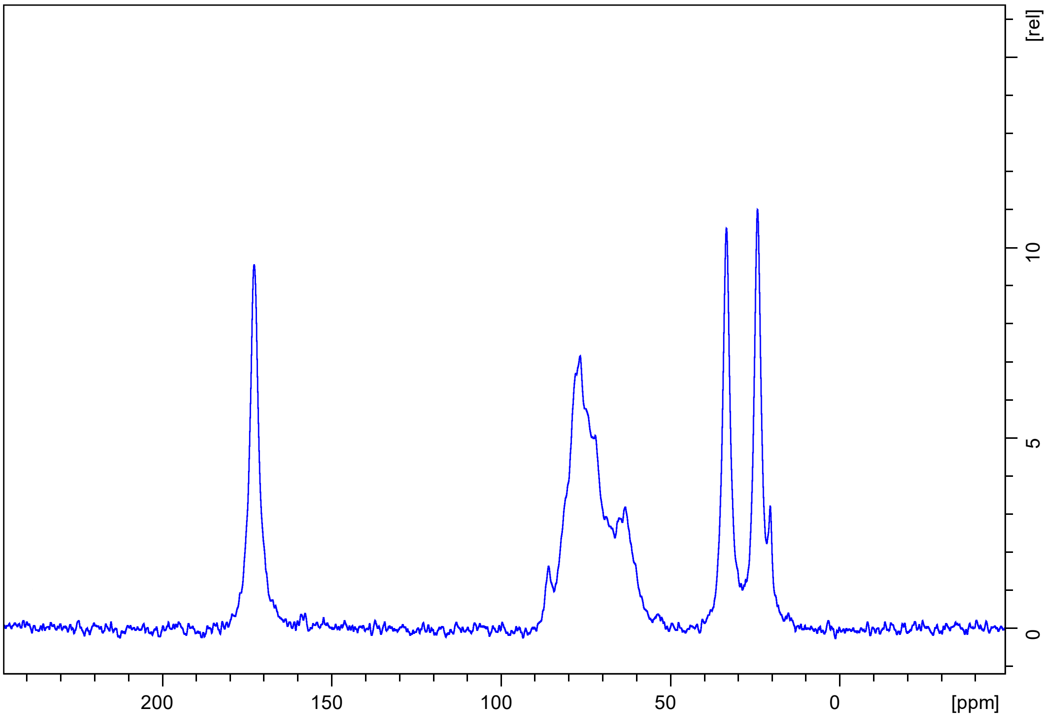

Figure S55. Solid state ^{13}C NMR spectrum of sample S_1. Conditions: sorbitol:CLM = 1:1 (wt/wt).

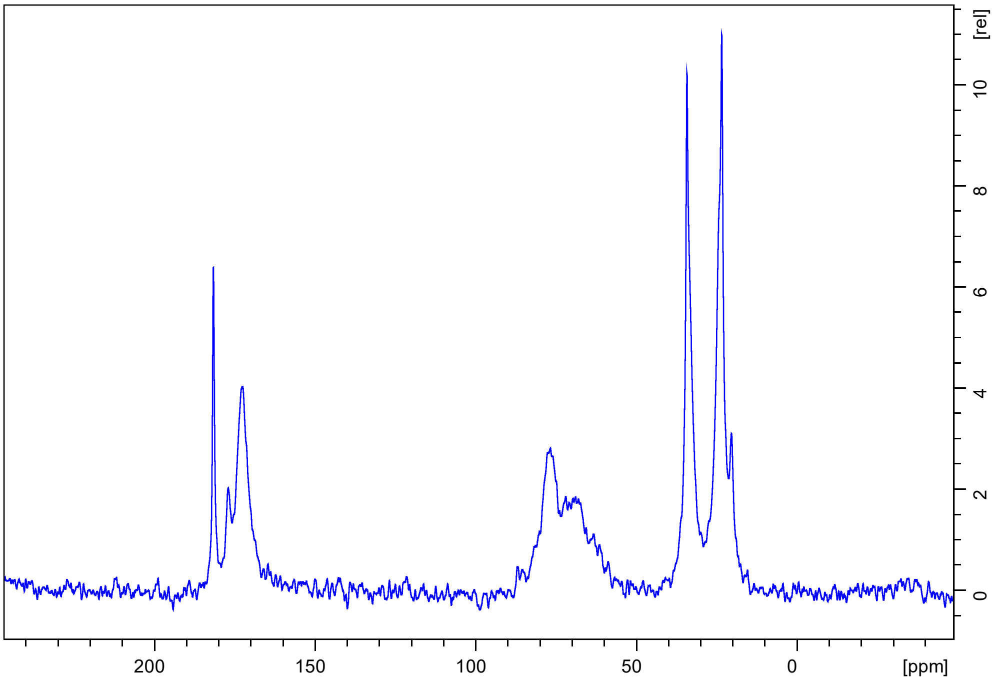

Figure S56. Solid state ^{13}C NMR spectrum of sample $S_{0.5}$. Conditions: sorbitol:CLM = 0.5:1 (wt/wt).

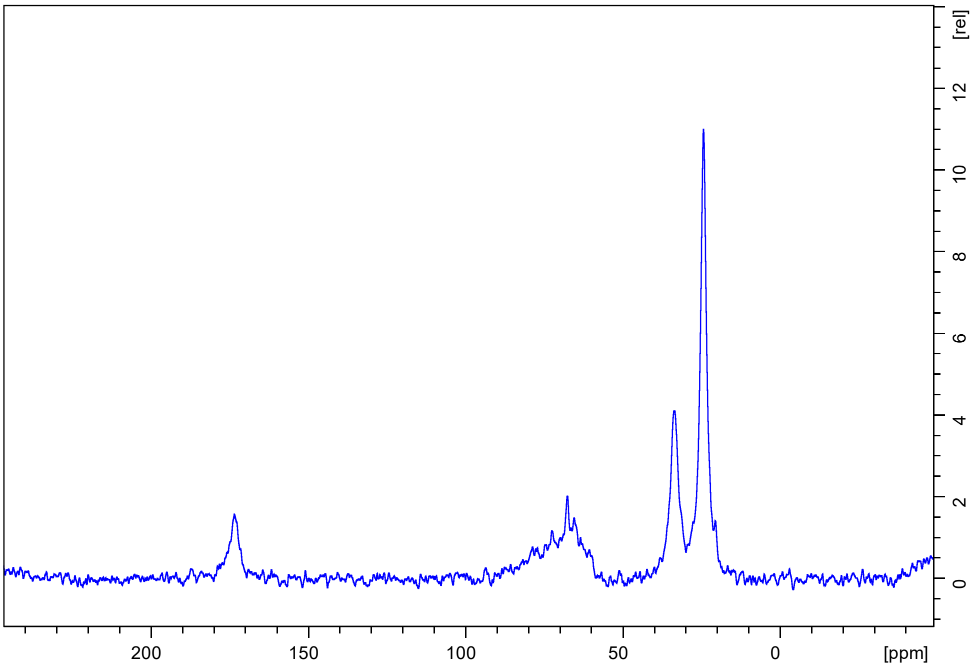

Figure S57. Solid state ^{13}C NMR spectrum of sample $GS_{0.5}$. Conditions: glycerol:sorbitol:CLM = 0.5:0.5:1 (wt/wt/wt).

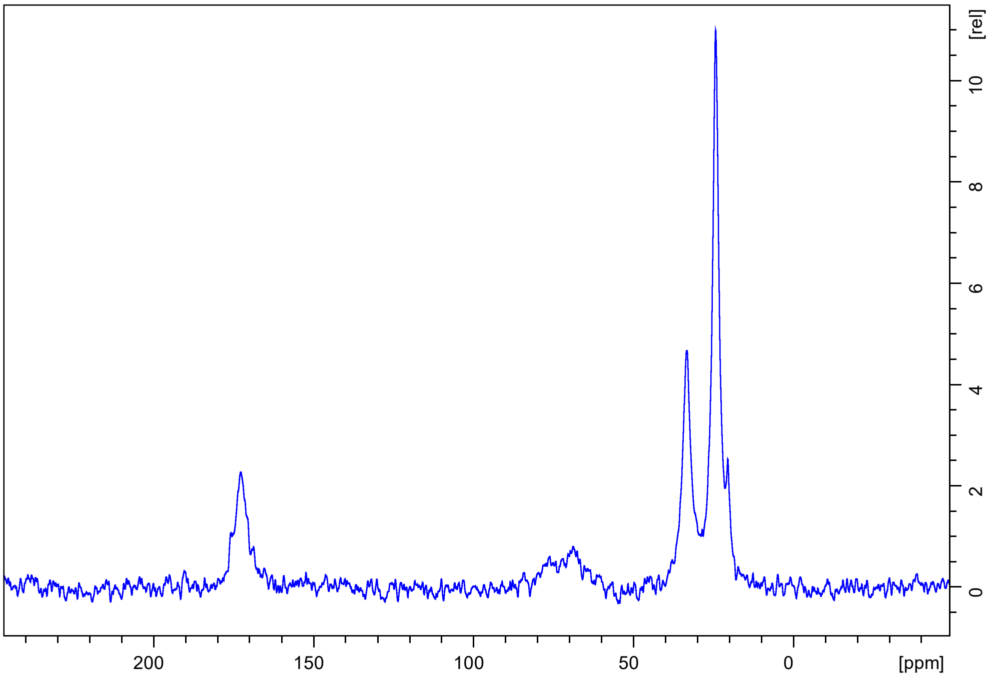

Figure S58. Solid state ^{13}C NMR spectrum of sample $GS_{0.25}$. Conditions: glycerol:sorbitol:CLM = 0.25:0.25:1 (wt/wt/wt).

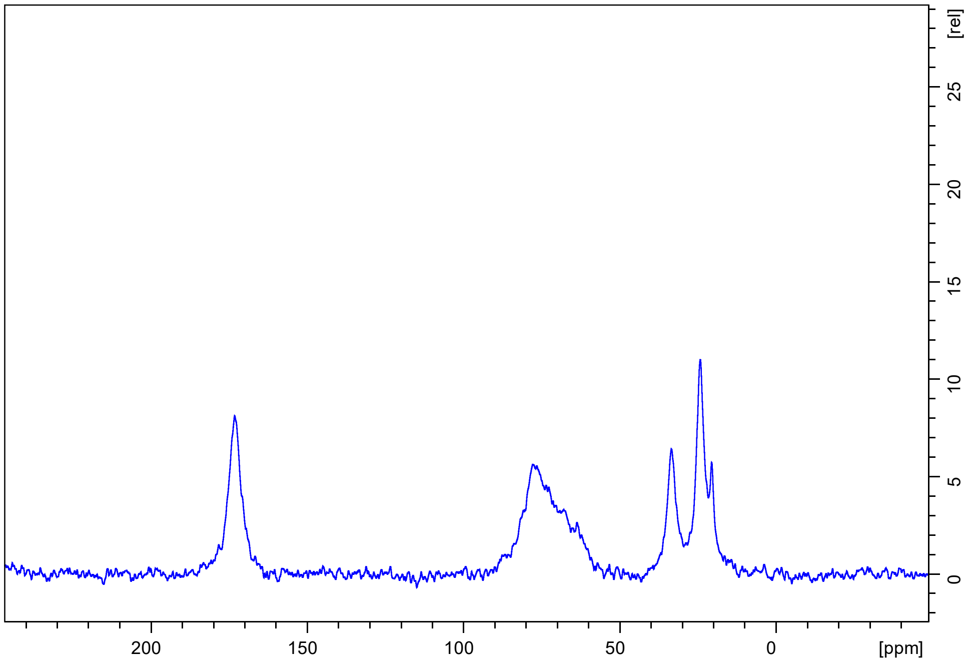

Figure S59. Solid state ^{13}C NMR spectrum of sample $G_{0.2}$ $S_{0.8}$. Conditions: glycerol:sorbitol:CLM = 0.2:0.8:1 (wt/wt/wt).

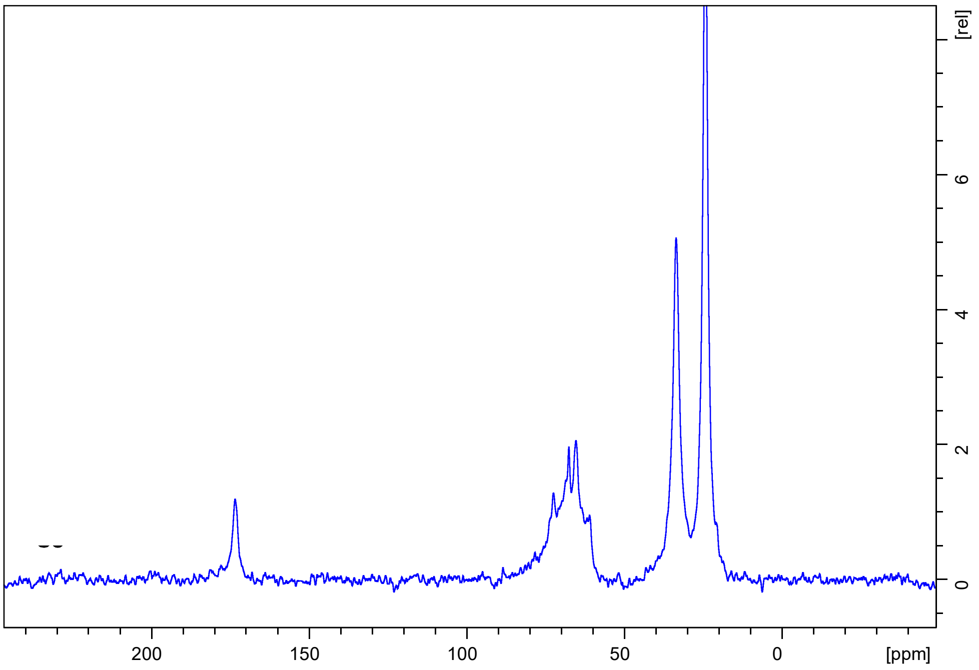

Figure S60. Solid state ^{13}C NMR spectrum of sample $G_{0.8}S_{0.2}$. Conditions: glycerol:sorbitol:CLM = 0.8:0.2:1 (wt/wt/wt).

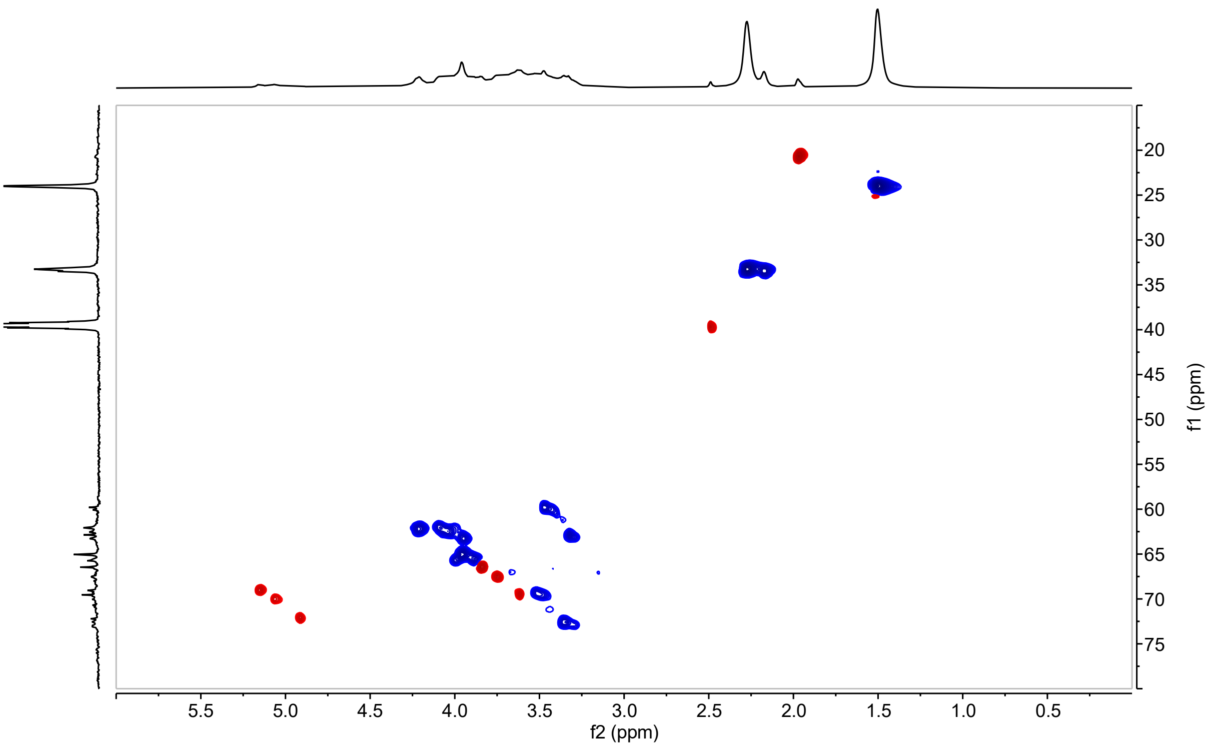

Figure S61. HR-MAS HSQC spectrum of swollen G_1. Solvent: DMSO-d_6.

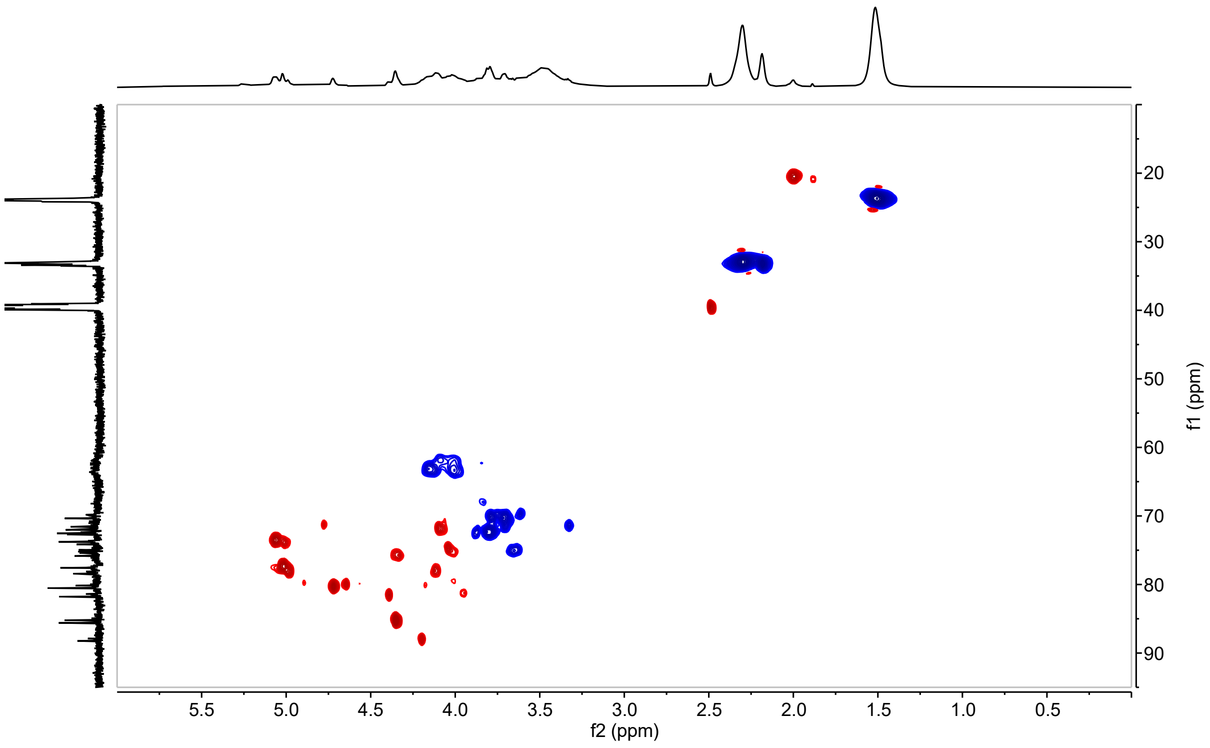

Figure S62. HR-MAS HSQC spectrum of swollen S_1. Solvent: DMSO-d_6.

Differential scanning calorimetry (DSC) curves

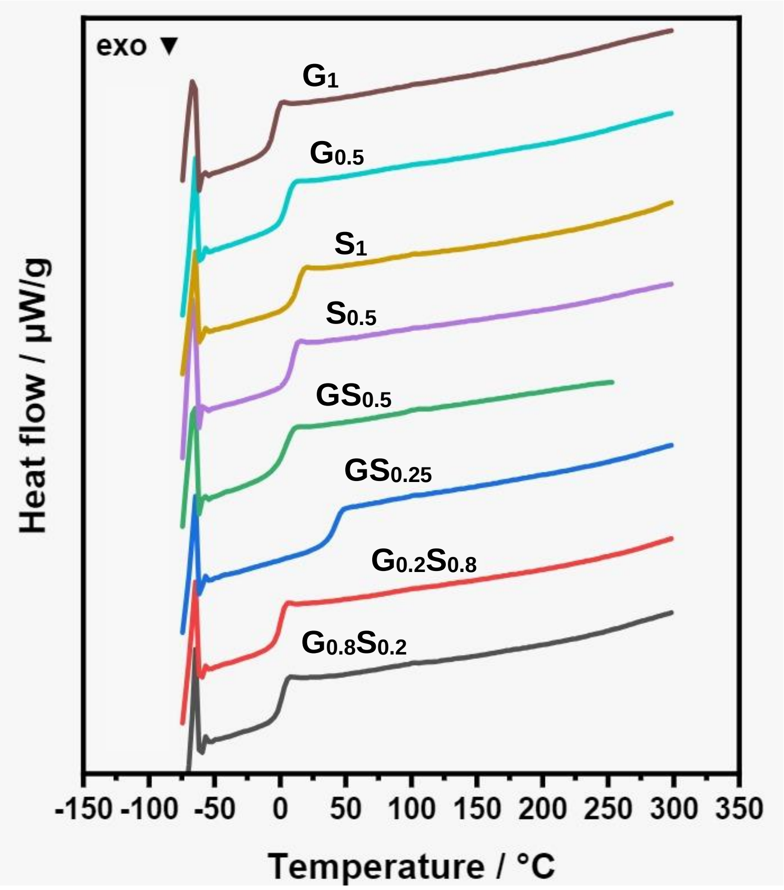

Figure S63. DSC curves for all the polyesters.

Dynamic mechanical analysis (DMA)

The DMA (dynamic mechanical analysis; dual cantiliver) was carried out on samples G_1 and S_1, in the temperature range of -50–150 °C, frequency. Below and above T_g, the value of the storage modulus (E’) was higher than the loss modulus (E’’) for both polymers, indicating that the elastic component is predominant over the viscous one. As expected, in correspondence with the tan δ peak a significant drop of E’, of about 2 orders of magnitude, is observed. The glass transition temperature, determined from the peak of tan δ, is equal to XX °C and YY°C for G_1 and S_1, respectively. The values are in good agreement with those calculated by DSC analysis.

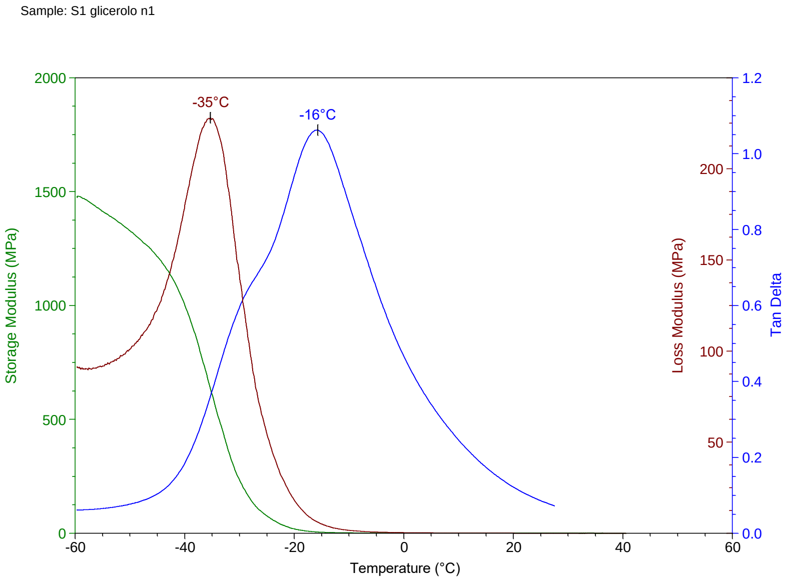

Figure S64. Dynamic mechanical analysis (DMA) for sample G_1.

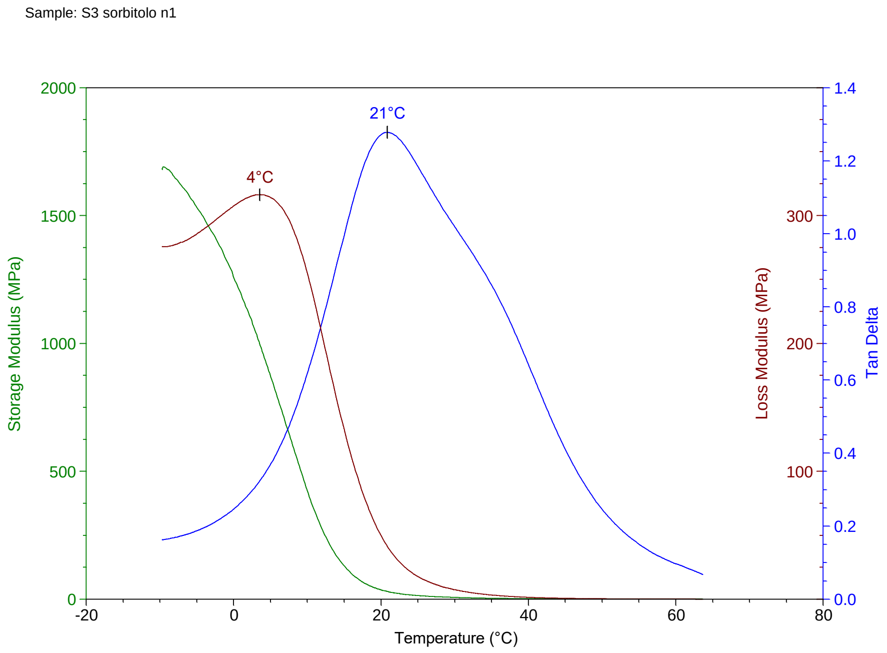

Figure S65. Dynamic mechanical analysis (DMA) for sample S_1.

The chemical recycling of the polyesters

Polyesters G_1 and S_1 were chemically recycled via methanolysis followed by liquid/liquid extraction. The polyester of choice (50 g) was set to react with a 1 wt% NaOH solution in MeOH (200 mL) at T = 50 °C for t = 1h in a round-bottom flask under stirring with a magnetic stir bar (400 rpm). Once the reaction was complete, the mixture was neutralized with 10mol% HCl, and the solvent was rotary evaporated (p = 5 mbar),

yielding a heterogeneous mixture of adipic acid methyl esters and sugar alcohols (see characterization data below). The mixture was then separated by liquid/liquid extraction using water and 2-methyl tetrahydrofuran (1:1 v/v; 100 mL each). The organic phase was rotary evaporated to obtain a light brown oil composed of adipic acid methyl esters (yield: 26.3g, 117 wt%, 99 mol%, and 25.4g, 113 wt%, 99 mol% for G_1 and S_1, respectively). The aqueous phase was treated with Amberlyst-36 for 1h at room temperature to remove the NaCl formed during the neutralization of NaOH, after which the resin was filtered, and the water was removed by rotary evaporation (p = 5 mbar). This procedure resulted in a slightly brown, highly viscous liquid composed of polyols (yield: 25.3g, 101wt%, 99mol%, and 19.5g, 79wt%, >99mol% for G_1 and S_1, respectively). The characterization of the obtained products after chemical recycling was performed via both GC-MS and ^{1}H NMR analyses (Figures S65-S92). From G_1, glycerol and diglycerols (linear and cyclic: 3,3'-oxybis(propane-1,2-diol), (1,4-dioxane-2,6-diyl)dimethanol), and (1,4-dioxane-2,5-diyl)dimethanol) were detected in the water phase, while adipic acid and its mono- and di-methyl esters were found in the organic phase. From S_1, sorbitol and sorbitans (1,4-anhydrosorbitol, and 1,5-anhydrosorbitol) were detected in the water phase, while adipic acid and its mono- and di-methyl esters were found in the organic phase. Noteworthy, other non-characterized sorbitol-derivates are expected to be present in the water phase after the depolymerization of S_1. Due to the complexity of the mixture of the water-soluble fraction obtained from S_1 (composed by isosorbide and sorbitans), acetylation of the latter mixture of products followed by GC-MS was performed to better characterized the obtained products. To carry out the acetylation, the mixture of sugar alcohols (0.1g) was treated with acetic anhydride (Ac_2O; 5 mL) and pyridine (5 mL) at room temperature for 16h under stirring with a magnetic stir bar (400 rpm). Once the reaction was complete, both Ac_2O and pyridine were removed by rotary evaporation and the mixture of product was analyzed by GC-MS, as mentioned. Such procedure corroborated the presence of isosorbide and 1,5-anhydrosorbitol in the water phase derived from S_1 (see chromatogram and MS spectra in Figures S78-S80). Even after acetylation it was difficult to identify other C6-sugar alcohols in this mixture. The evaluation of the molar recovery of the products with respect to the initial adipic acid, glycerol (for G_1) and sorbitol (for S_1) was performed via ^{1}H NMR using benzoic acid as the internal standard (Figures S81-92). The results are reported in Table S6 and S7.

Table S6. Recovery of adipic acid and its methyl esters, and sugars alcohols after the depolymerization of G_1. The results have been evaluated by ^{1}H NMR using benzoic acid as the internal standard.

Compound	Functional group	Integration ranges (ppm)	Number of protons	Recovery (mol%)	Total recovery (mol%)[c]
DMAd	-C**H**$_2$-C(=O)-	2.26-2.46[1]	2	86	99[a]
MMAd+AdA	-C(=O)-OC**H**$_3$	3.65[1]	3	13	
Glycerol	-C**H**$_2$(OH)-	3.49-3.55	2	78	99[b]
Diglycerols	-C**H**(OH)-	3.75-3.80[25]	1	21	

[a]Sum of DMAdA and MMAdA+AdA. [b]Sum of glycerol and diglycerols. [c]Molar recovery of all the products with respect to the amount of adipic acid or polyol present in the initial CLM.

Table S7. Recovery of adipic acid and its methyl esters, and sugars alcohols after the depolymerization of S_1. The results have been evaluated by ^1H NMR using benzoic acid as the internal standard.

Compound	Functional group	Integration ranges (ppm)	Number of protons	Recovery (mol%)	Total recovery (mol%)[c]
DMAdA	-C**H_2**-C(=O)-	2.26-2.46[1]	2	47	99[a]
MMAdA+AdA	-C(=O)-OC**H_3**	3.65[1]	3	52	
Isosorbide	-C**H**(-OH)-	4.54 (t)	1	25	>99[b]
Sorbitans	CH_2-C**H**(-CH_2OH)-O-	3.59-3.64 (dd)[26]	1	26	
	-O-C**H_2**-CH(-OH)-	4.15-4.17 (m)[27,28]	2	20	
	O-C**H**(-CH_2OH)-	3.96-3.98 (dd)[29]	1	28	

[a]Sum of DMAdA and MMAdA+AdA. [b]Sum of isosorbide, and sorbitans. [c]Molar recovery of all the products with respect to the amount of adipic acid or polyol present in the initial CLM.

The adipic acid mono- and di-methyl esters were eventually converted into adipic acid via a facile acid catalyzed hydrolysis procedure. The methyl adipates mixtures obtained from the depolymerization of G_1 and S_1 (26.3g and 25.4g, respectively) were placed in an autoclave with a 0.1% (v/v) H_2SO_4 solution in H_2O (100 mL) for 4 hours at 150°C under stirring. After the reaction, the water was removed by rotary evaporation, yielding a white solid, consisting of adipic acid (22.3g and 22.4g from the methyl adipates obtained from G_1 and S_1 respectively; yield >99%), which was characterized by ^1H-NMR, and ^{13}C-NMR.

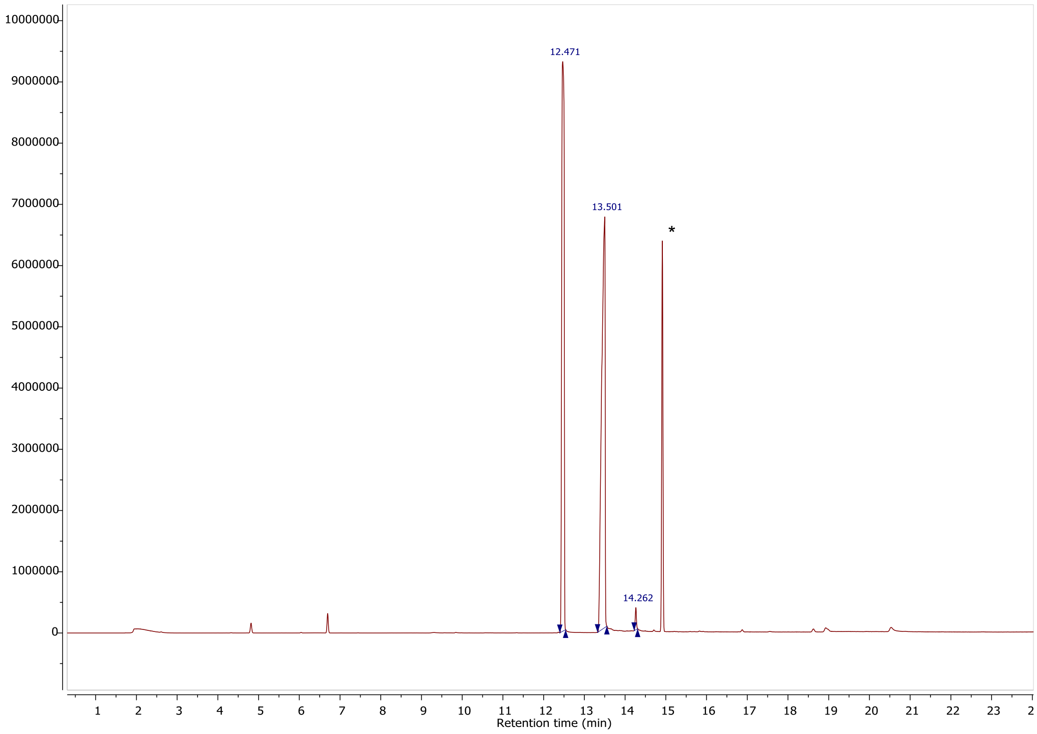

Figure S66. Typical chromatogram of a mixture of adipic acid and its methyl esters. *Indicates the residual 2,6-Di-*tert*-butyl-4-methylphenol (BHT; stabilizer) from liq/liq extraction with 2-Methyltetrahydrofuran

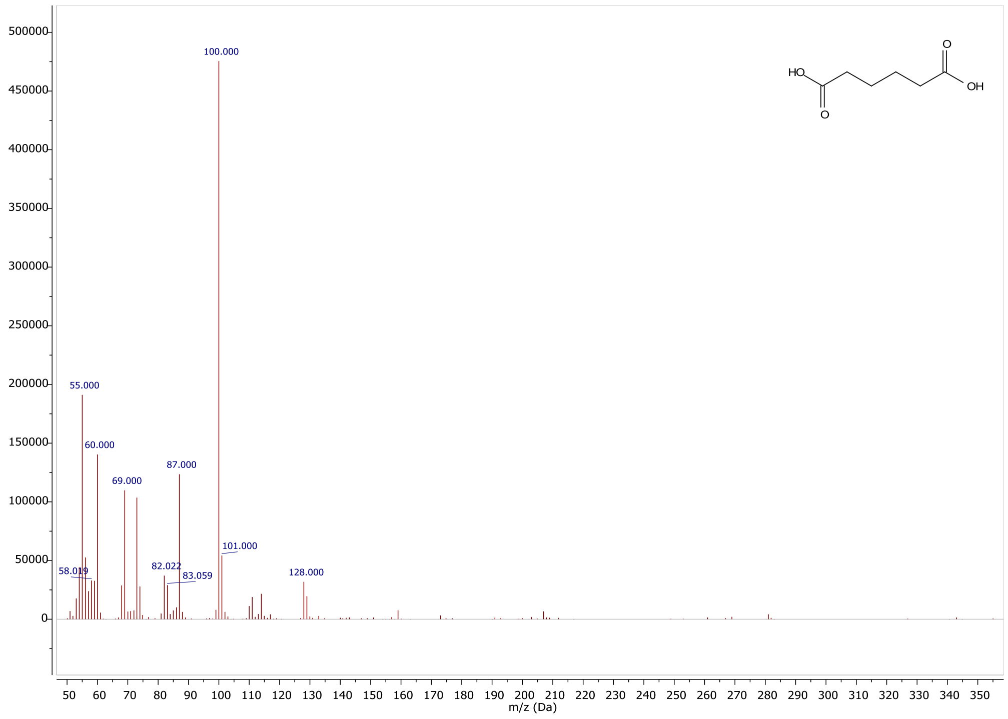

Figure S67. Mass spectrum of adipic acid (EI, 70eV).

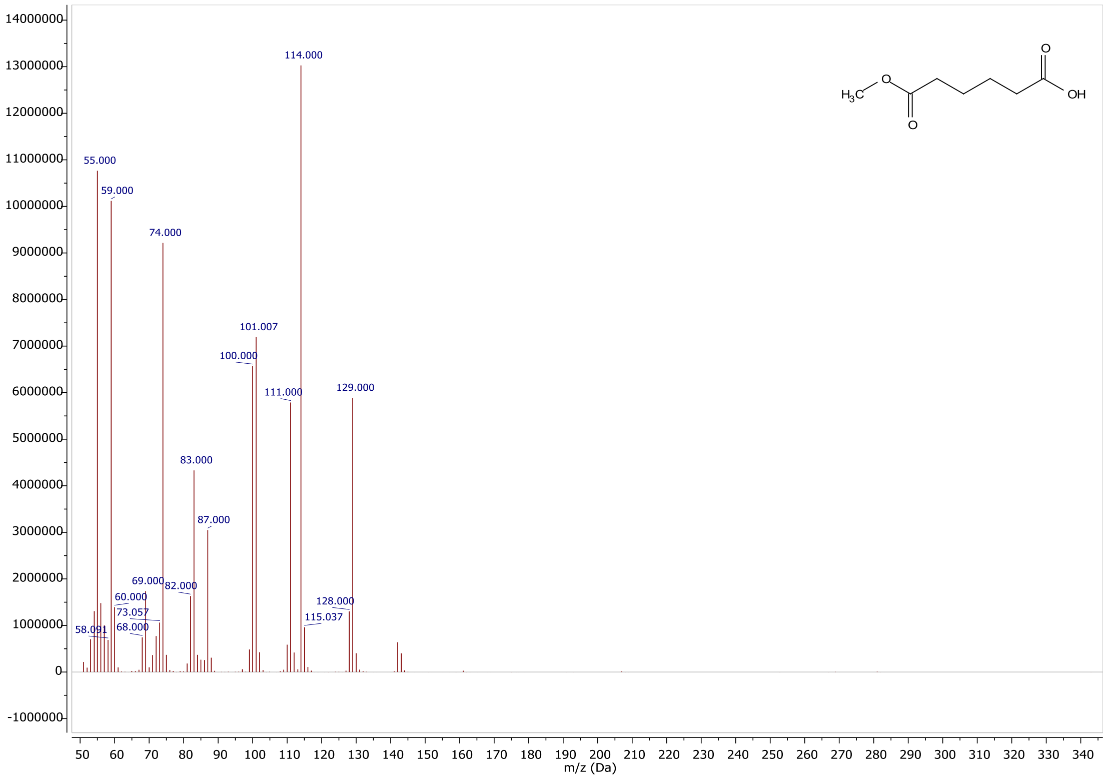

Figure S68. Mass spectrum of methyl adipate (EI, 70eV).

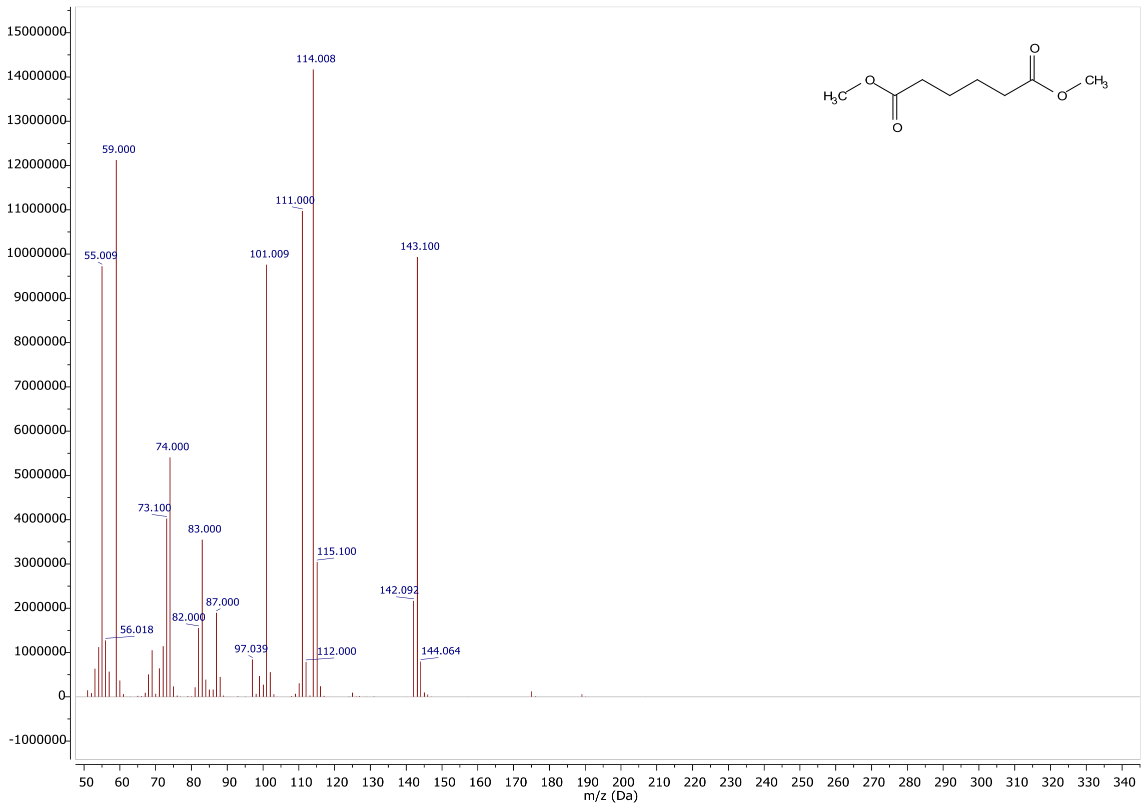

Figure S69. Mass spectrum of dimethyl adipate (EI, 70eV).

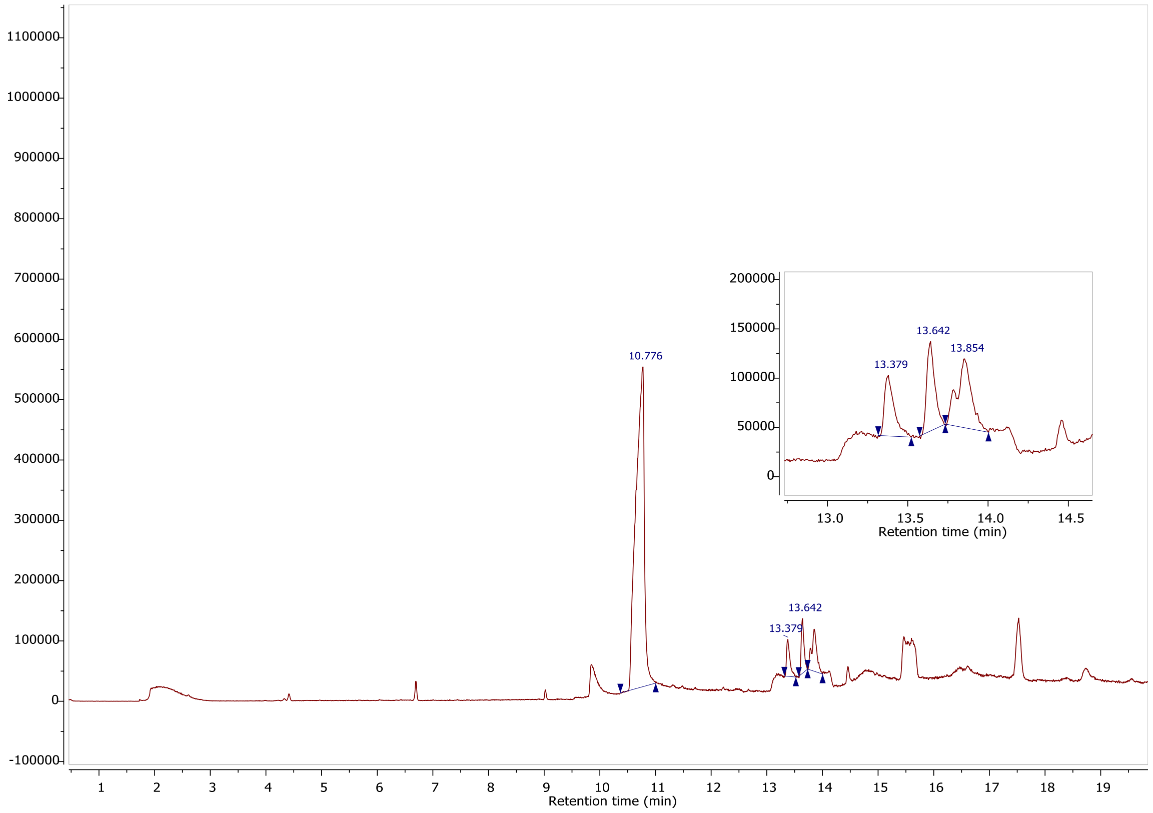

Figure S70. Chromatogram of the mixture of glycerol and glycerol ethers.

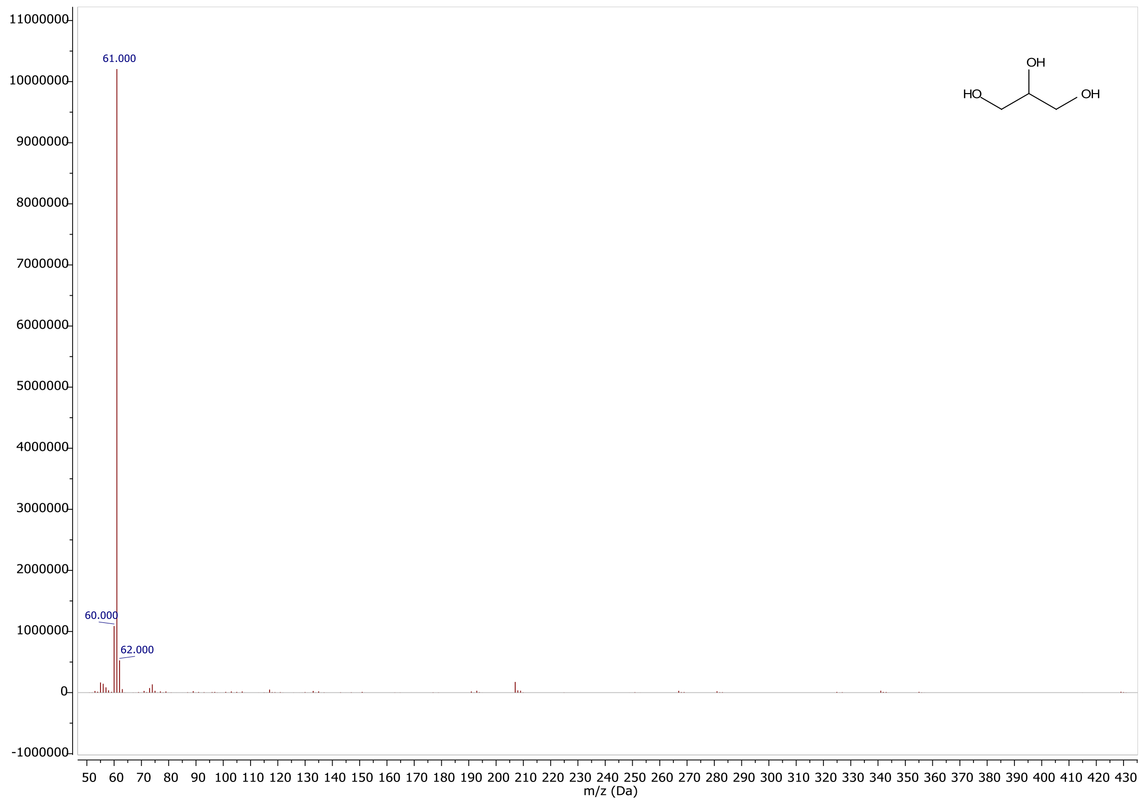

Figure S71. Mass spectrum of glycerol (EI, 70eV).

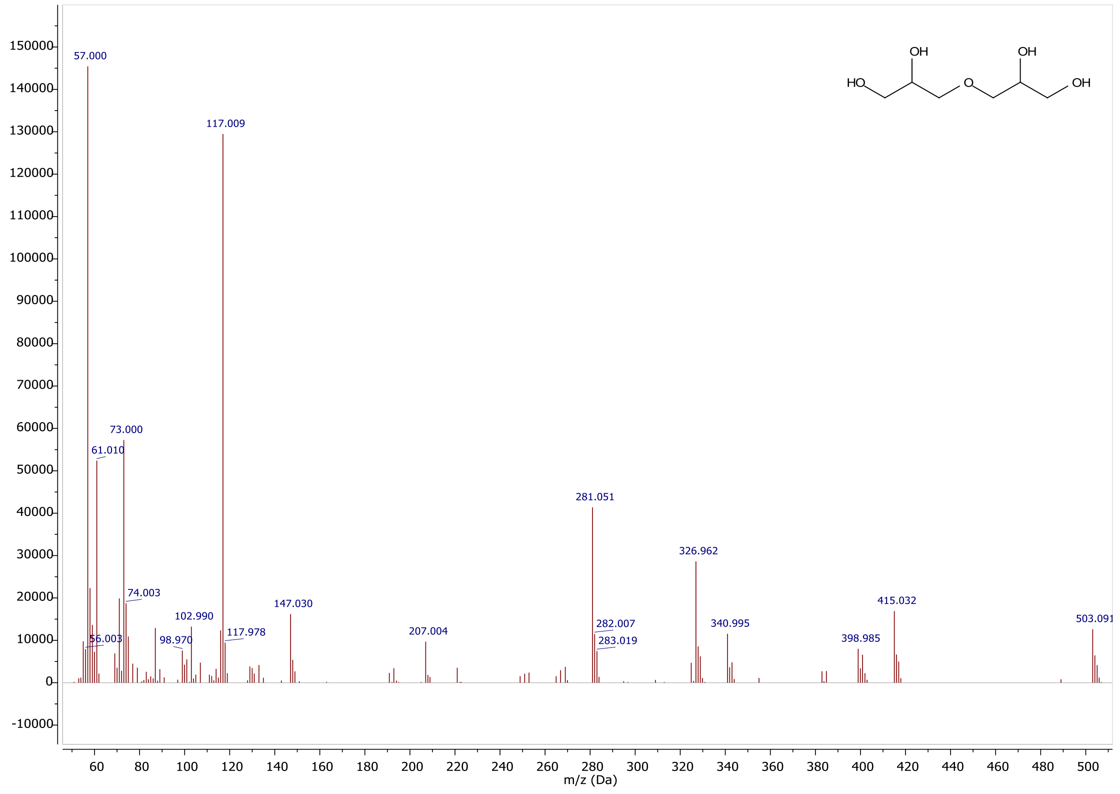

Figure S72. Mass spectrum of 3,3'-oxybis(propane-1,2-diol) (EI, 70eV).

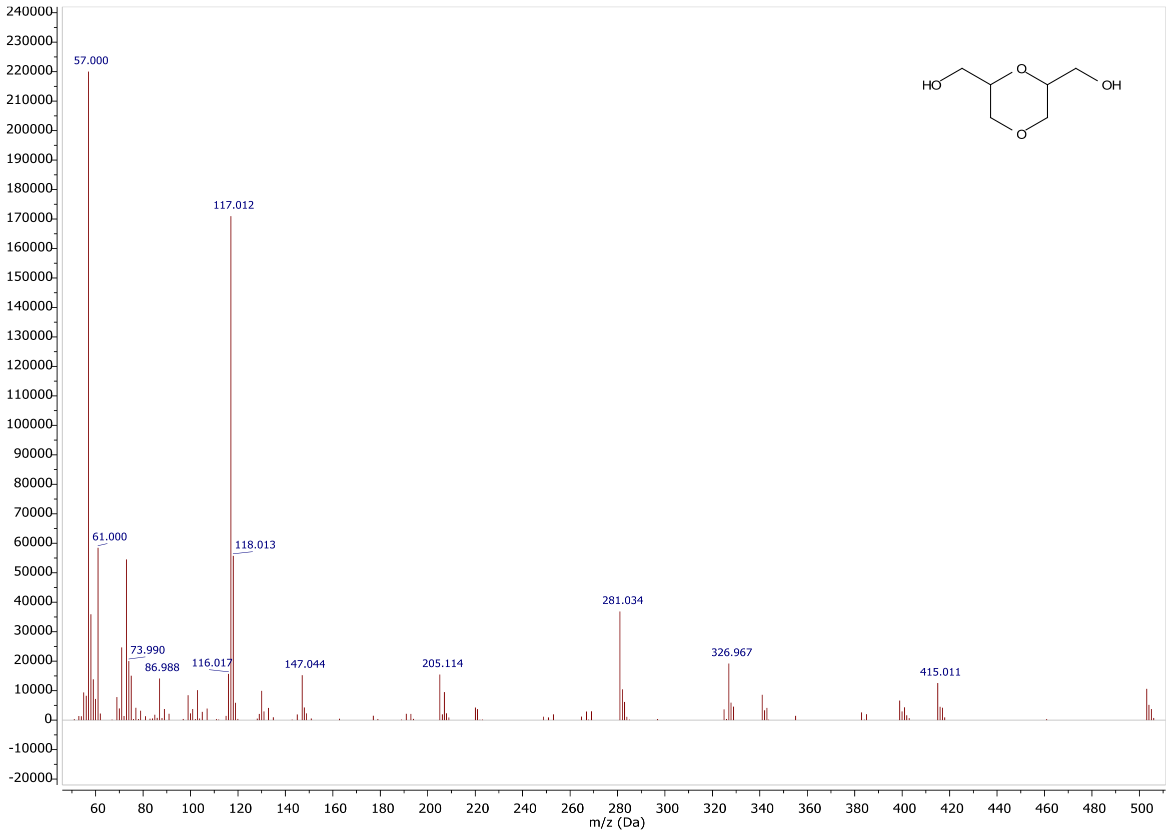

Figure S73. Mass spectrum of (1,4-dioxane-2,6-diyl)dimethanol (EI, 70eV).

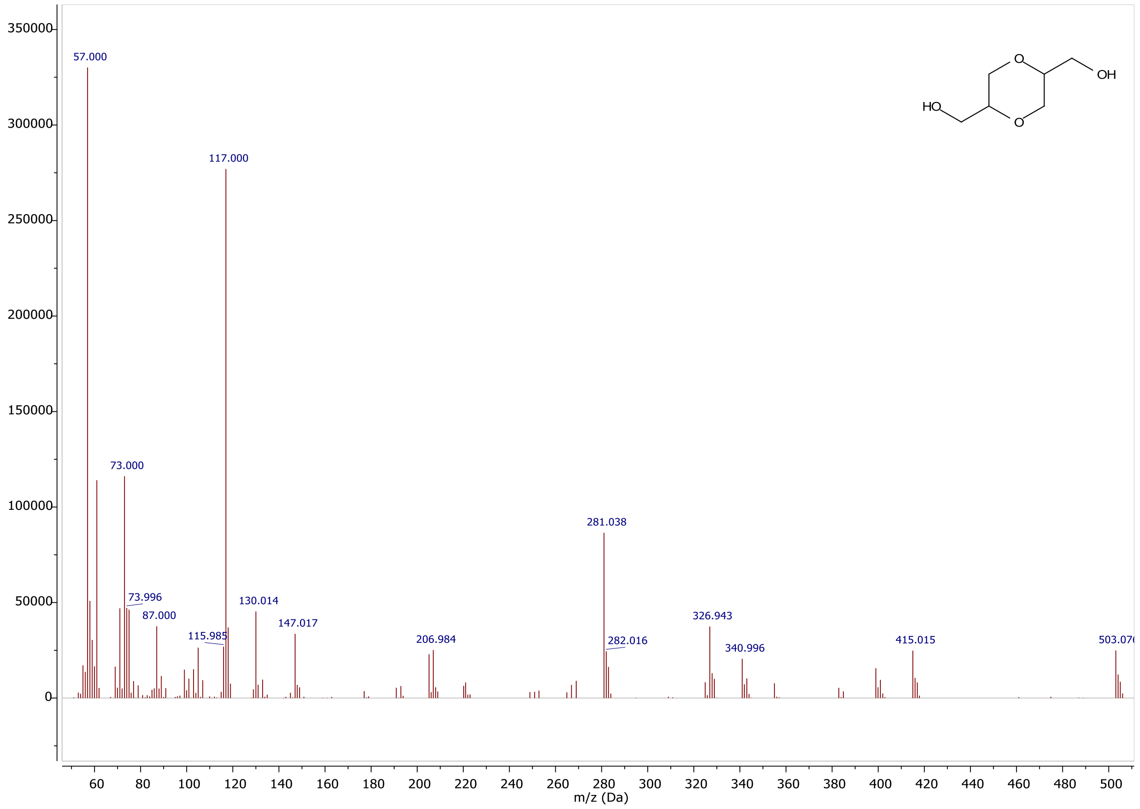

Figure S74. Mass spectrum of (1,4-dioxane-2,5-diyl)dimethanol (EI, 70eV).

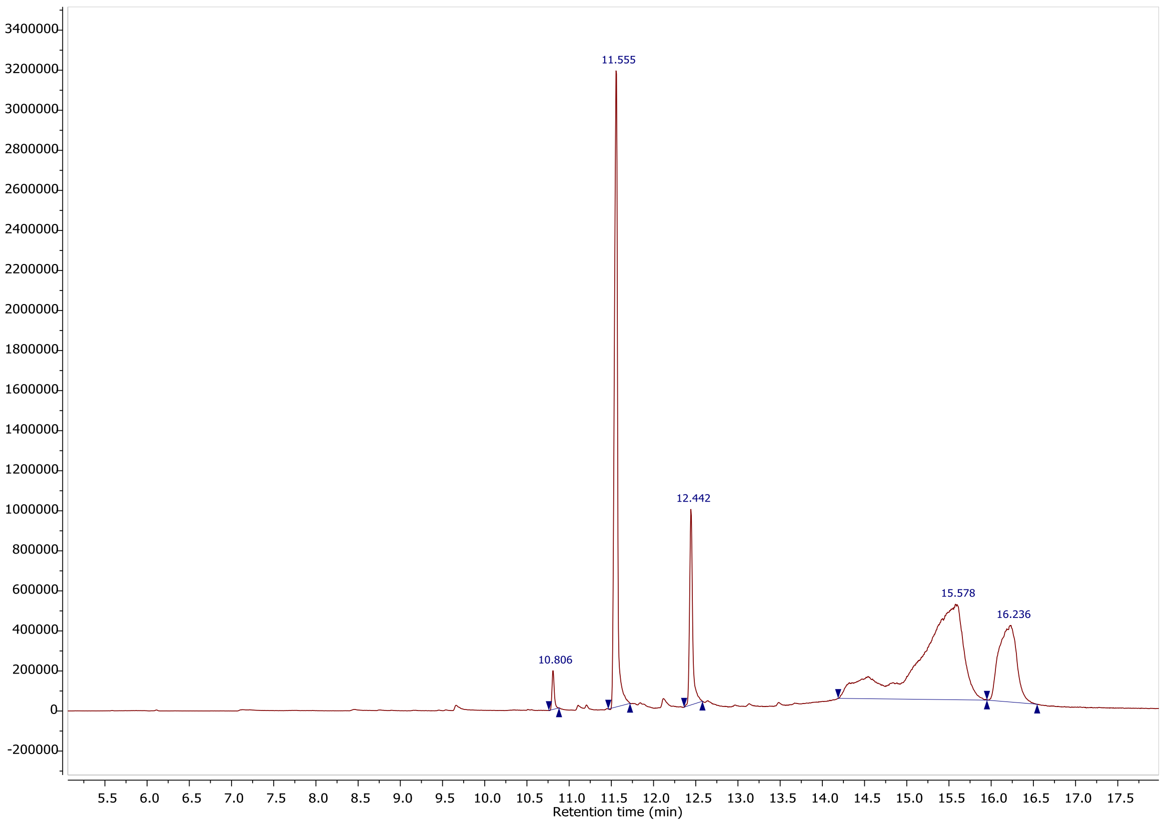

Figure S75. Chromatogram of the mixture of sorbitol and sorbitans.

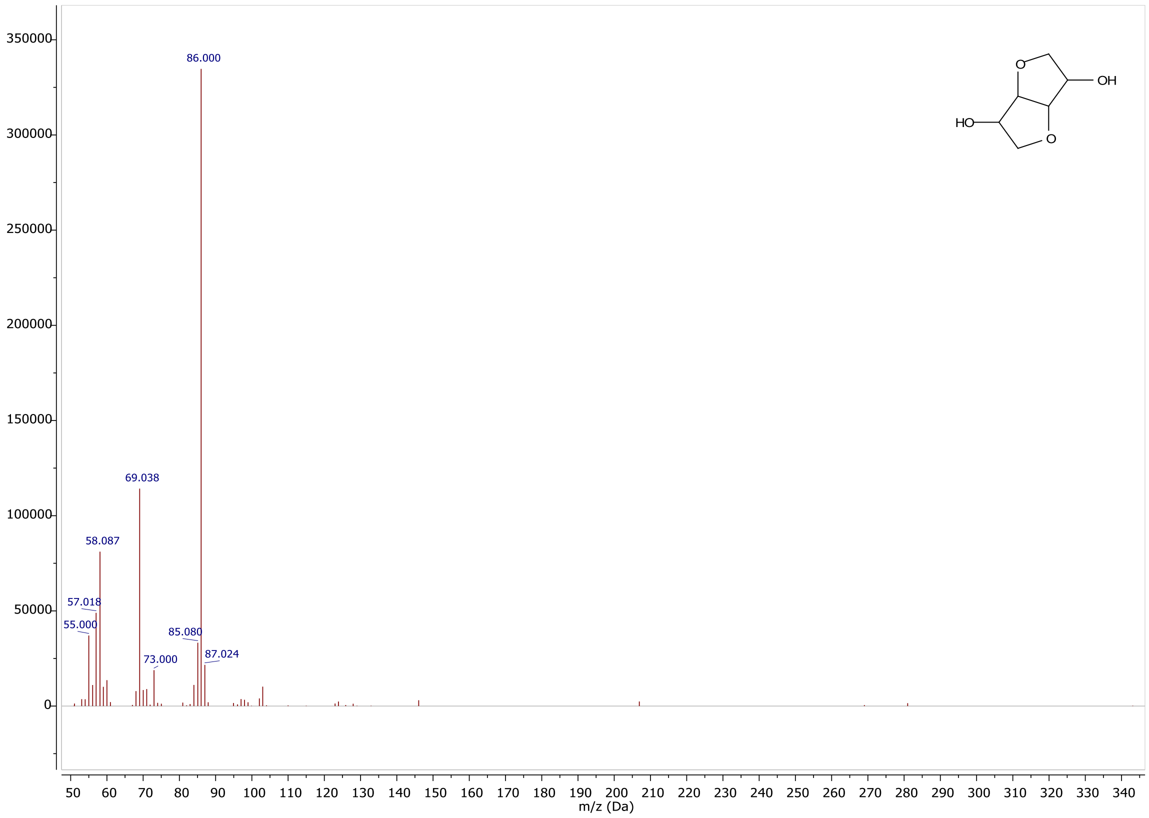

Figure S76. Mass spectrum of isosorbide (EI, 70eV).

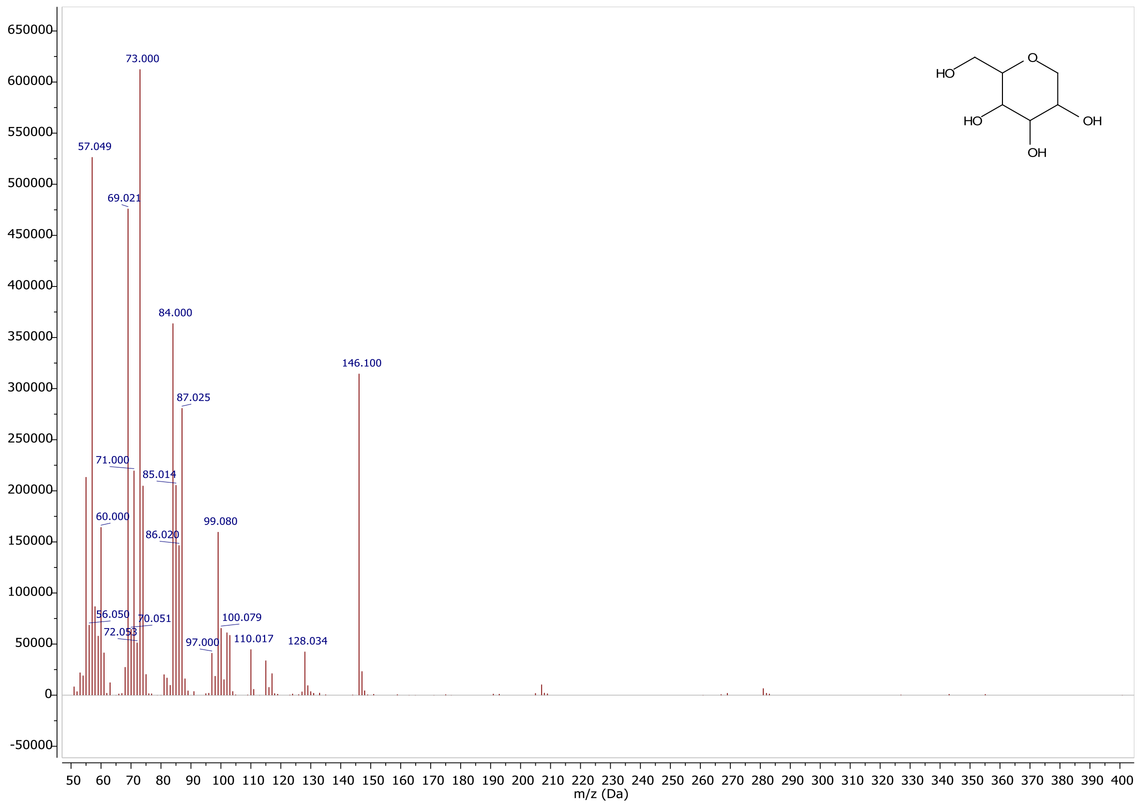

Figure S77. Mass spectrum of 2-(hydroxymethyl)tetrahydro-2H-pyran-3,4,5-triol (1,5-anhydrosorbitol) (EI, 70eV).

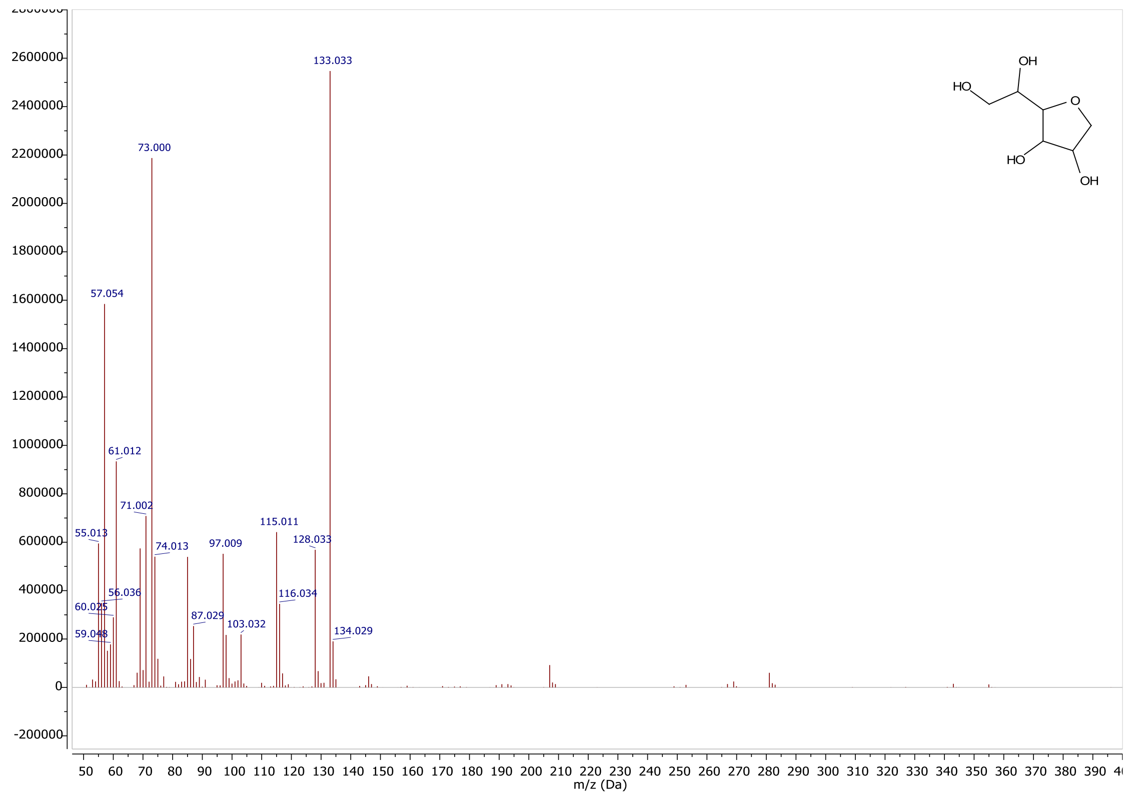

Figure S78. Mass spectrum of 2-(1,2-dihydroxyethyl)tetrahydrofuran-3,4-diol (1,4-anhydrosorbitol) (EI, 70eV).

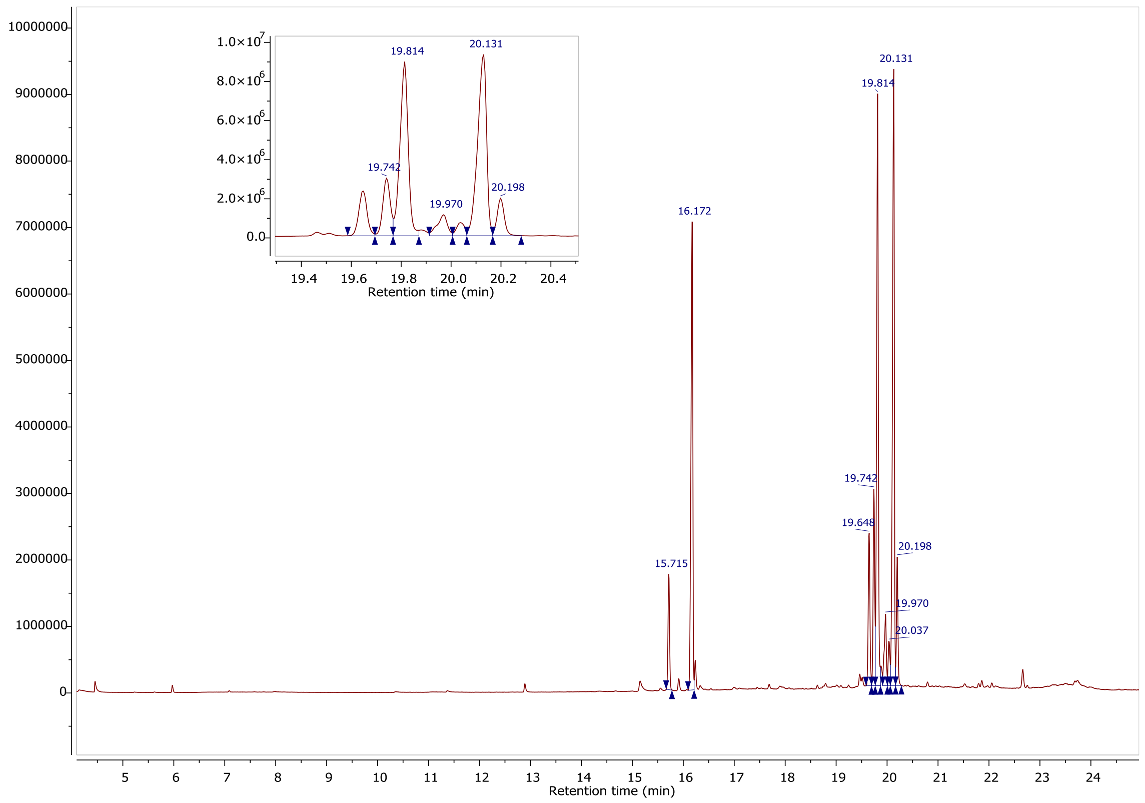

Figure S79. Chromatogram of the mixture of acetylated sorbitol and sorbitans.

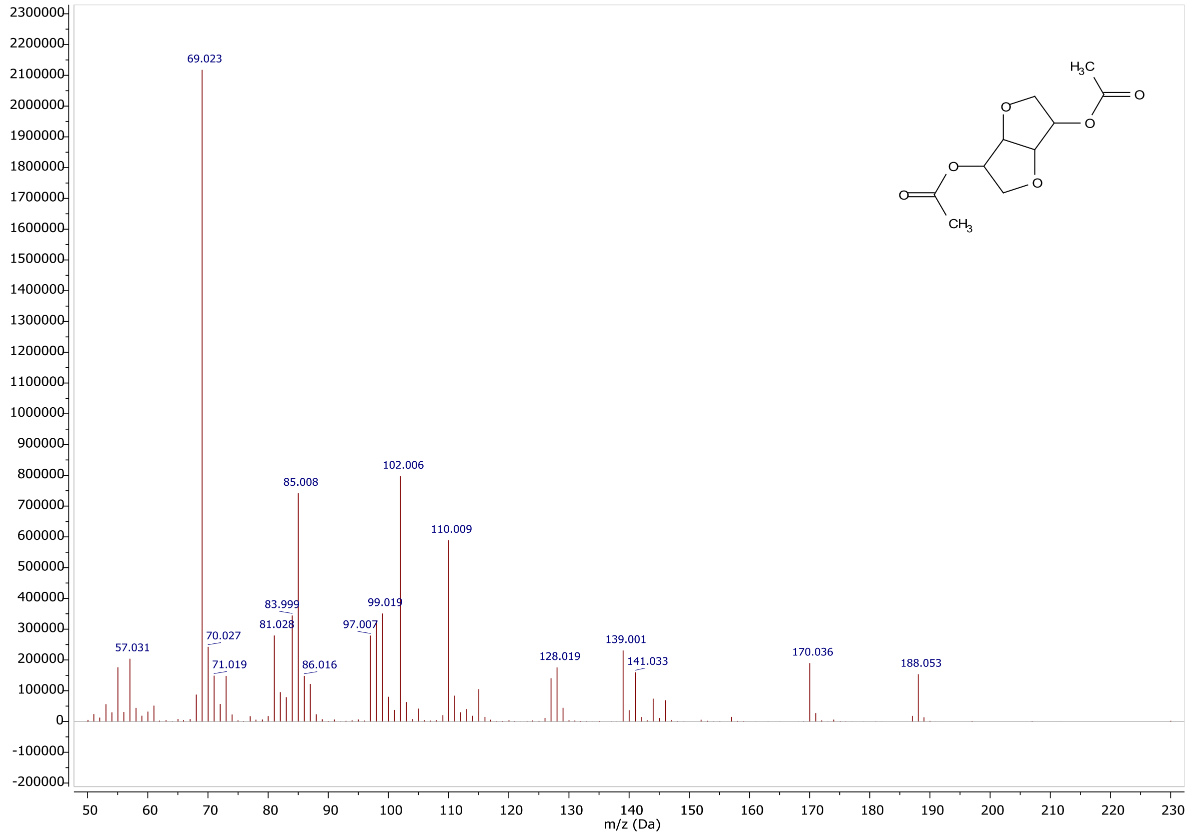

Figure S80. Mass spectrum of diacetyl isosorbide (EI, 70eV).

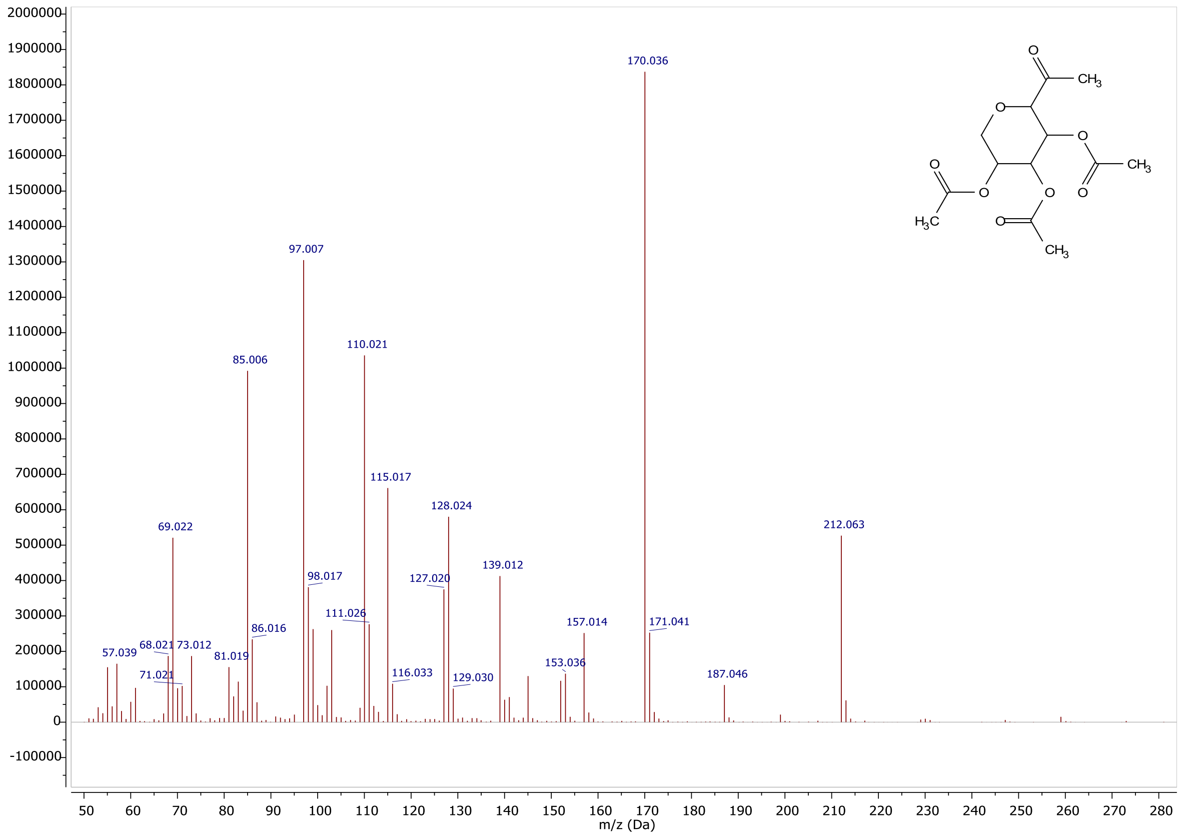

Figure S81. Mass spectrum of 2-(acetoxymethyl)tetrahydro-2H-pyran-3,4,5-triyl triacetate (peracetylated 1,5-anhydrosorbitol) (EI, 70eV).

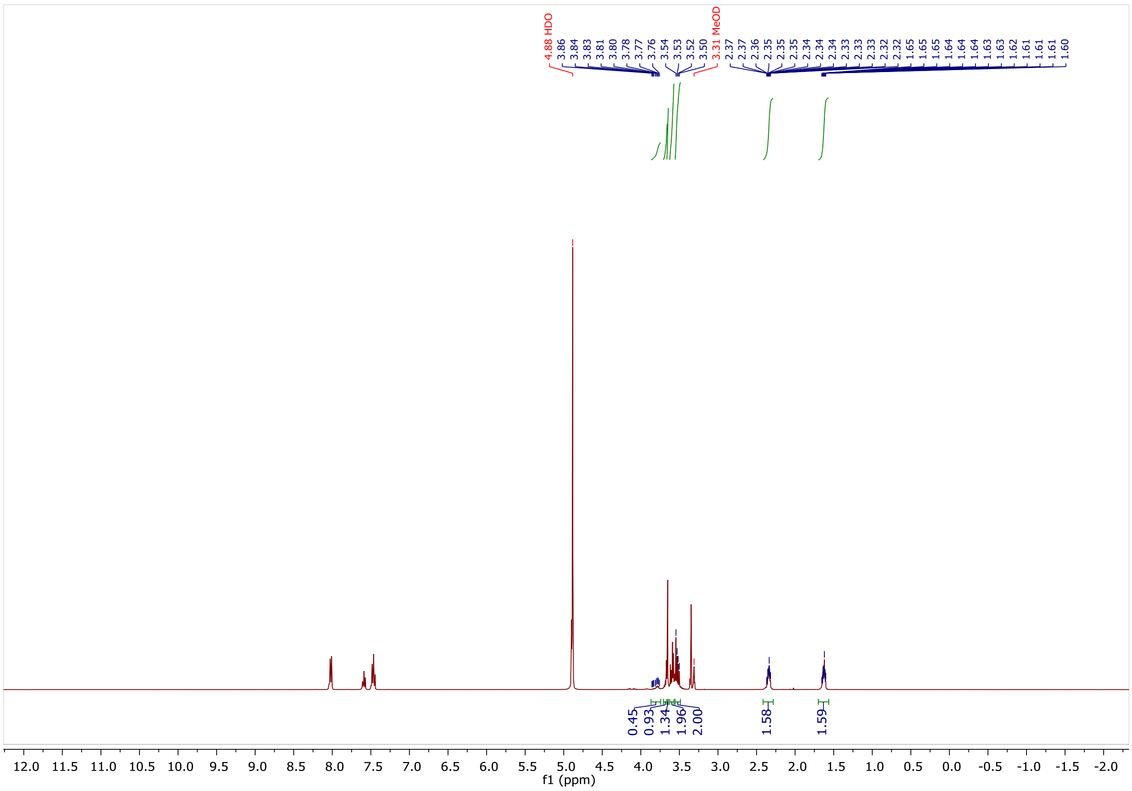

Figure S82. ^{1}H NMR spectrum of the crude depolymerization mixture obtained from G_1. Solvent: MeOH-d_4. The aromatic signals are referred to benzoic acid as the internal standard.

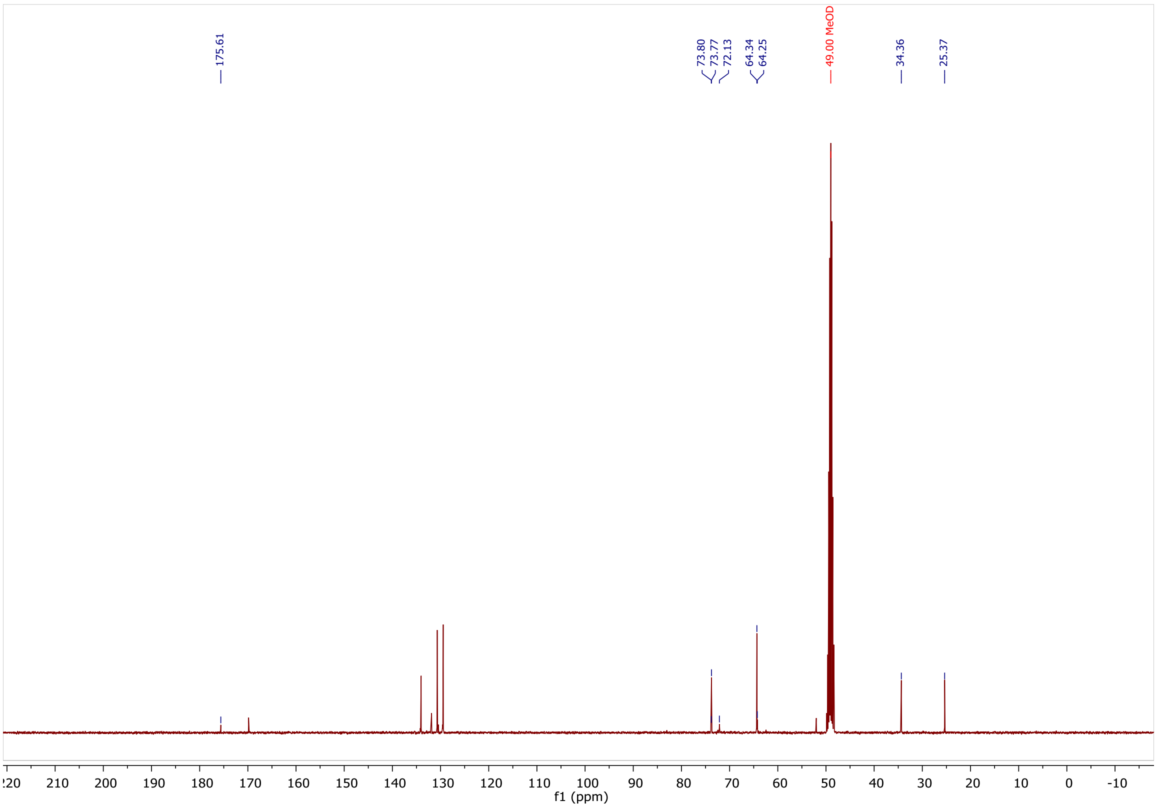

Figure S83. ^{13}C NMR spectrum of the crude depolymerization mixture obtained from G_1. Solvent: MeOH-d_4. The aromatic signals are referred to benzoic acid as the internal standard.

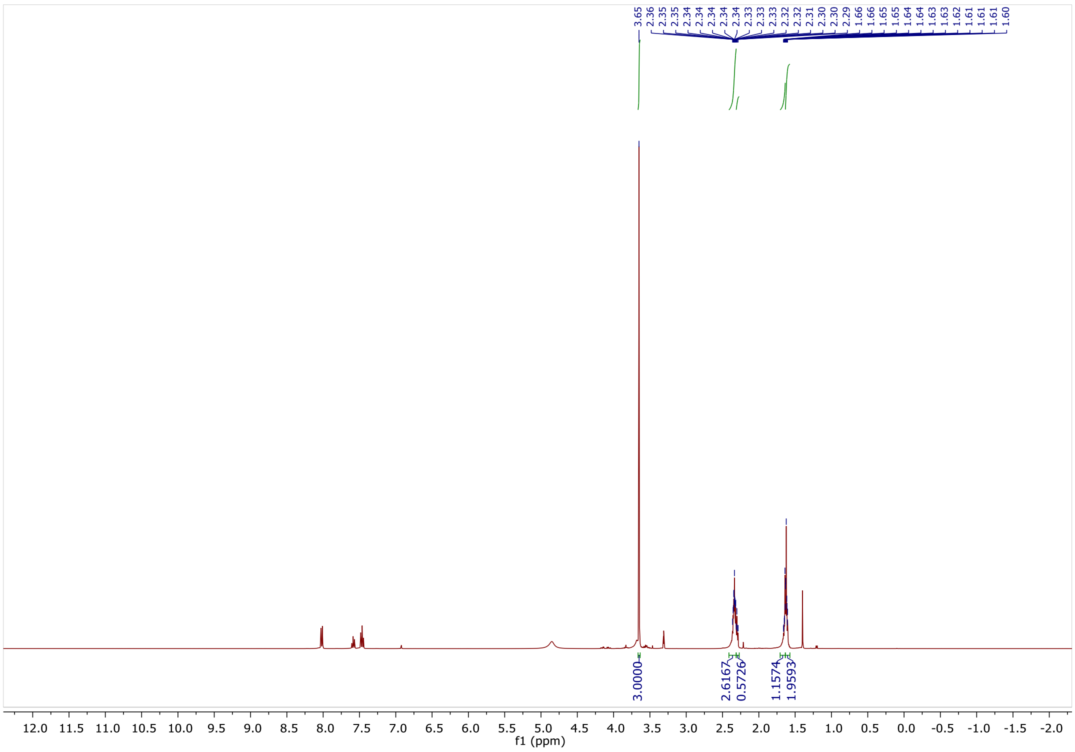

Figure S84. ^{1}H NMR spectrum of the 2MeTHF-soluble fraction (methyl esters of adipic acid) obtained from the depolymerization of G_1. Solvent: MeOH-d_4. The aromatic signals are referred to benzoic acid as the internal standard.

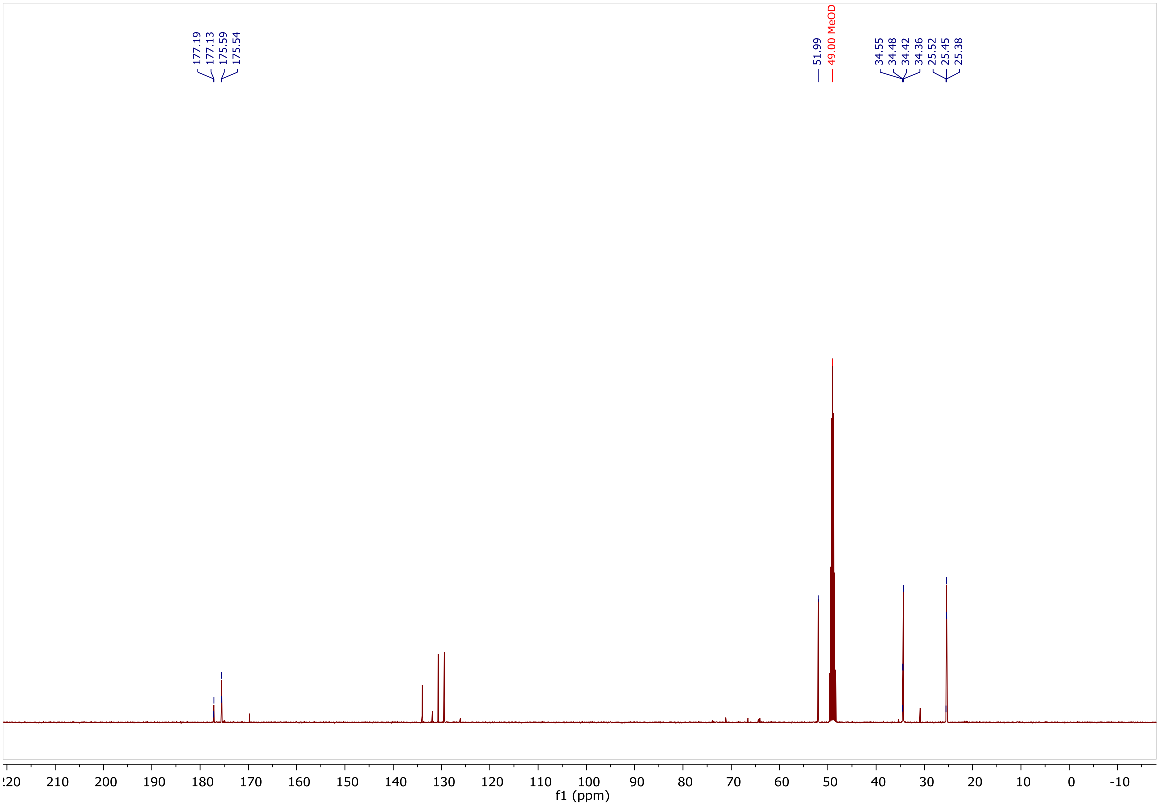

Figure S85. ^{13}C NMR spectrum of the 2MeTHF-soluble fraction (methyl esters of adipic acid) obtained from the depolymerization of G_1. Solvent: MeOH-d_4. The aromatic signals are referred to benzoic acid as the internal standard.

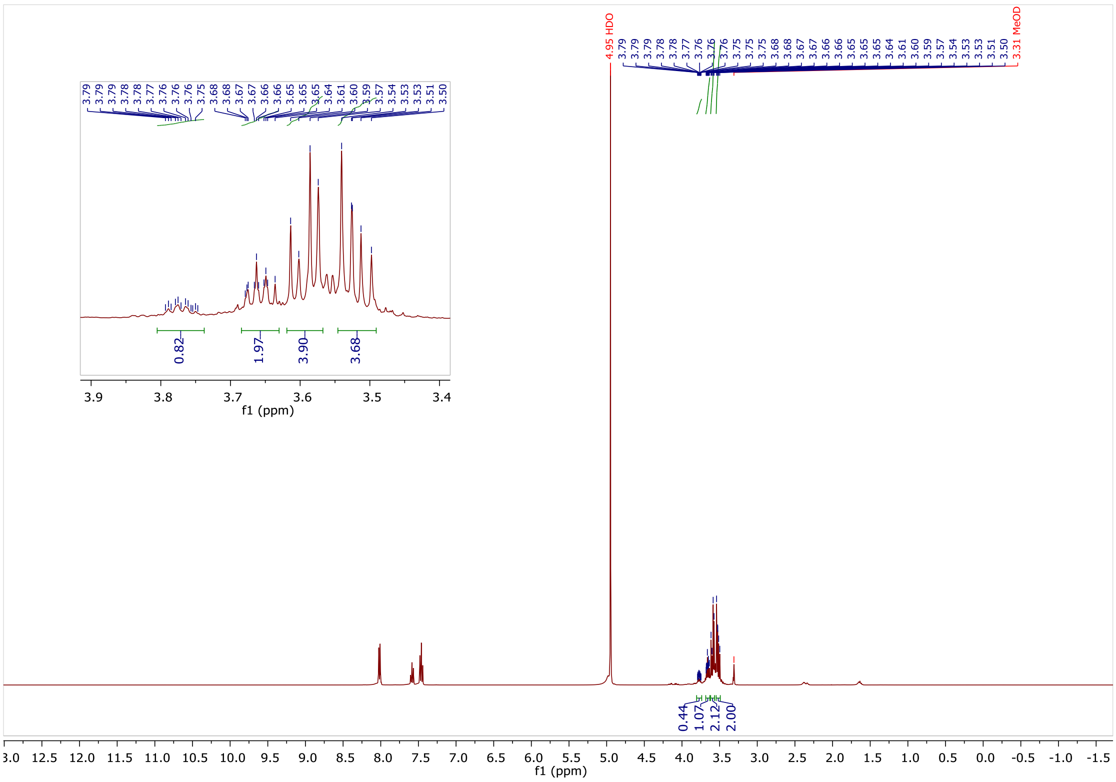

Figure S86. ^{1}H NMR spectrum of the H_2O-soluble fraction (glycerol and diglycerols) obtained from the depolymerization of G_1. Solvent: MeOH-d_4. The aromatic signals are referred to benzoic acid as the internal standard.

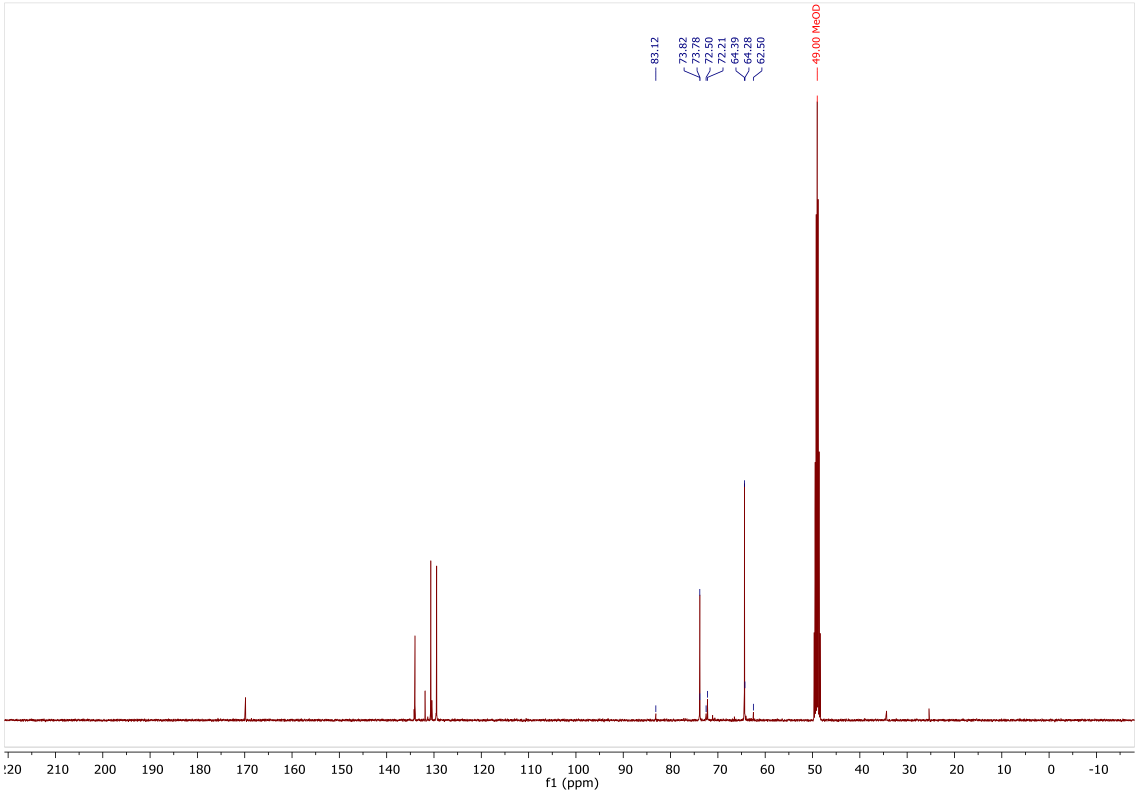

Figure S87. ^{13}C NMR spectrum of the H_2O-soluble fraction (glycerol and diglycerols) obtained from the depolymerization of G_1. Solvent: MeOH-d_4. The aromatic signals are referred to benzoic acid as the internal standard.

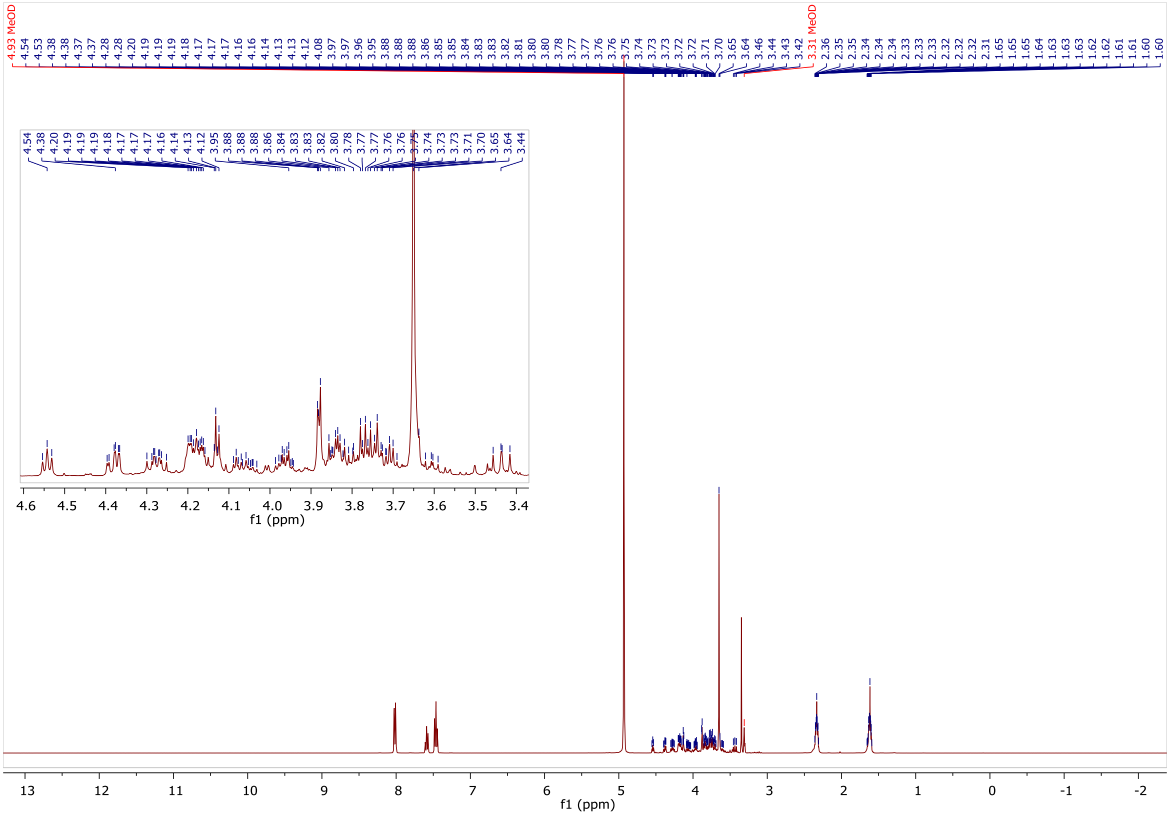

Figure S88. ^{1}H NMR spectrum of the crude depolymerization mixture obtained from S_1. Solvent: MeOH-d_4. The aromatic signals are referred to benzoic acid as the internal standard.

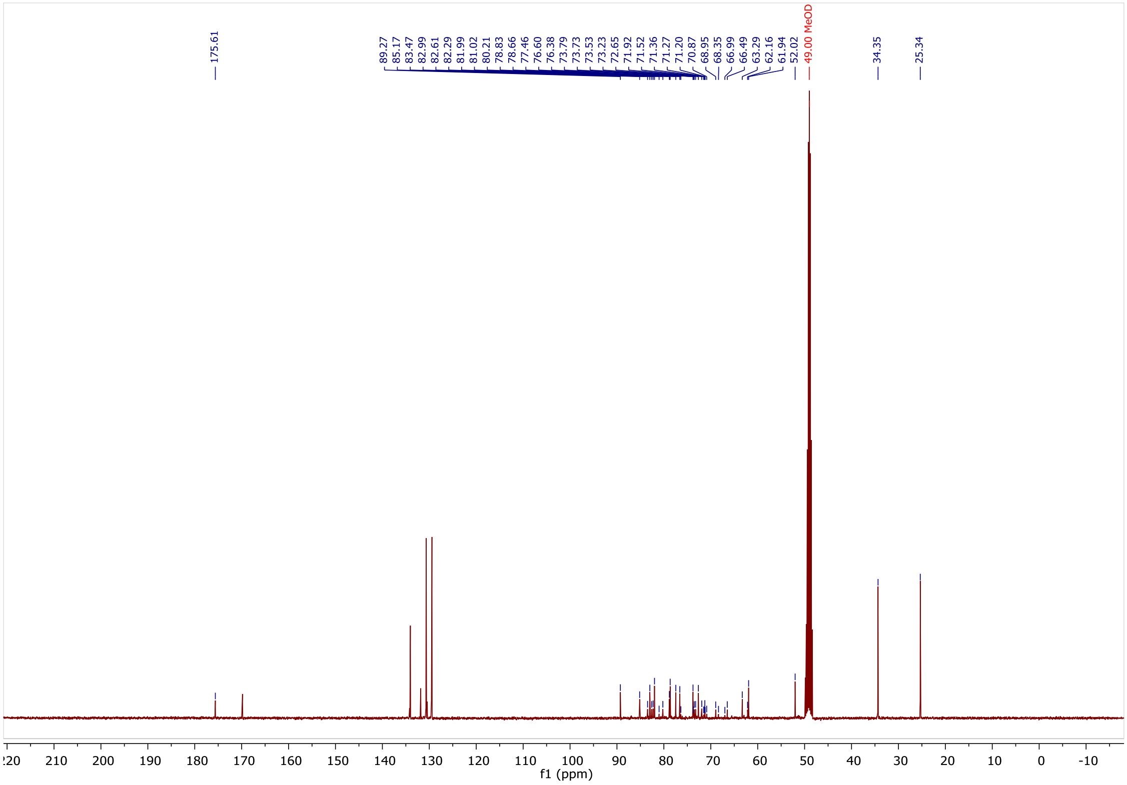

Figure S89. ^{13}C NMR spectrum of the crude depolymerization mixture obtained from S_1. Solvent: MeOH-d_4. The aromatic signals are referred to benzoic acid as the internal standard.

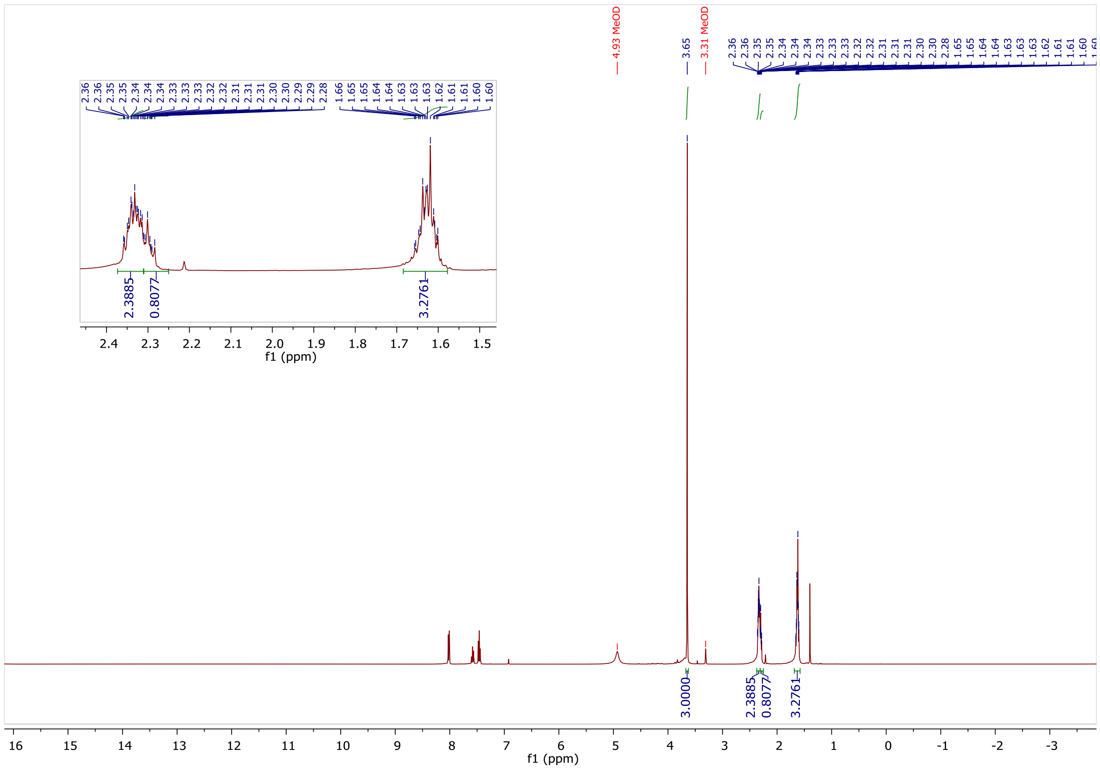

Figure S90. ^{1}H NMR spectrum of the 2MeTHF-soluble fraction (methyl esters of adipic acid) obtained from the depolymerization of S_1. Solvent: MeOH-d_4. The aromatic signals are referred to benzoic acid as the internal standard.

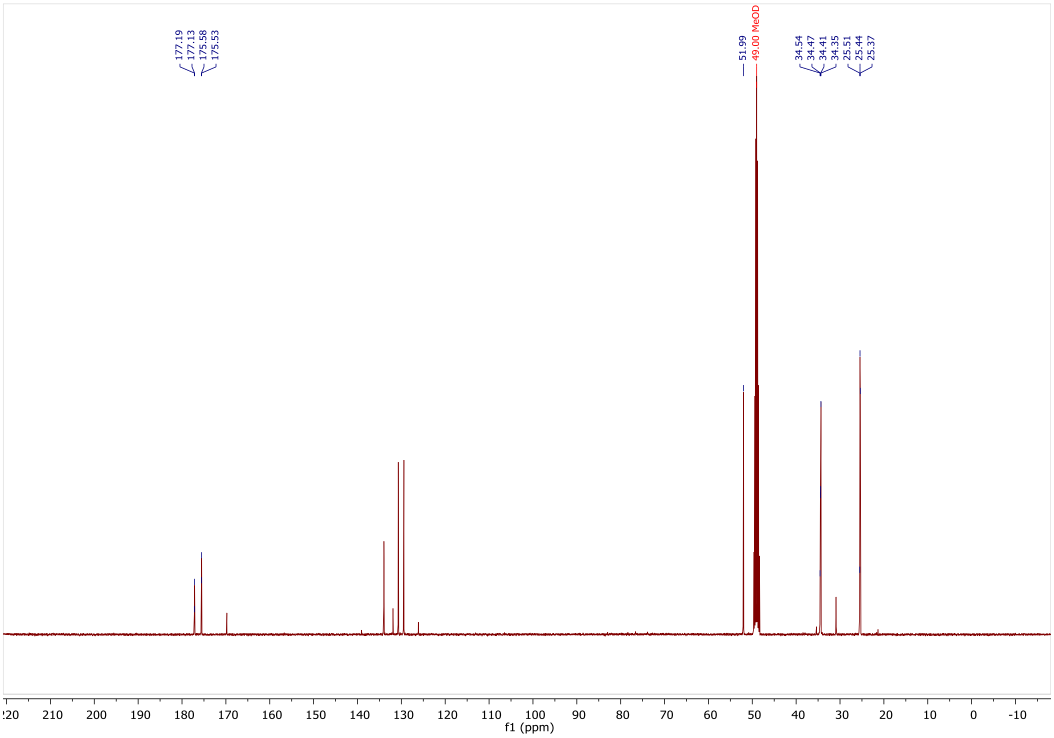

Figure S91. ^{13}C NMR spectrum of the 2MeTHF-soluble fraction (methyl esters of adipic acid) obtained from the depolymerization of S_1. Solvent: MeOH-d_4. The aromatic signals are referred to benzoic acid as the internal standard.

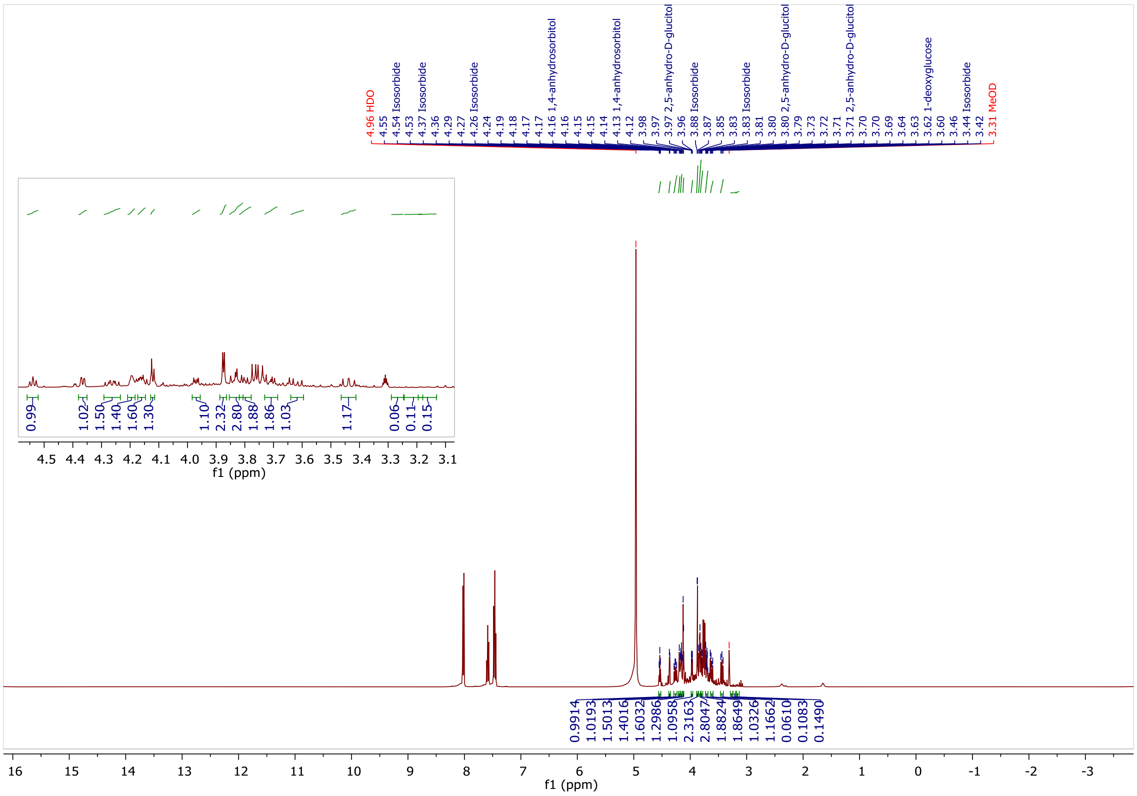

Figure S92. ^{1}H NMR spectrum of the H_2O-soluble fraction (isosorbide and sorbitans) obtained from the depolymerization of S_1. Solvent: MeOH-d_4. The aromatic signals are referred to benzoic acid as the internal standard.

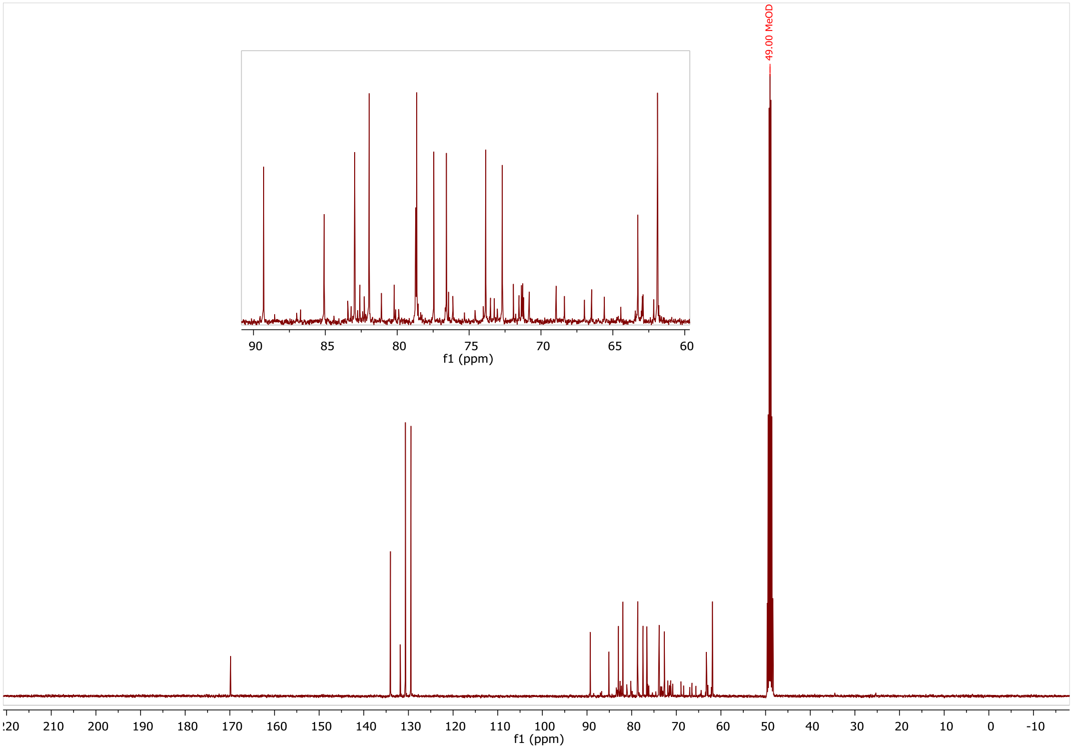

Figure S93. ^{13}C NMR spectrum of the H_2O-soluble fraction (isosorbide and sorbitans) obtained from the depolymerization of S_1. Solvent: MeOH-d_4. The aromatic signals are referred to benzoic acid as the internal standard.

The characterization of the recycled polyesters G_1 and S_1

Table S8. Properties of the recycled polyesters from glycerol (r-G_1) and sorbitol (r-S_1)

Entry	Sample	$T_{d5\%}$ (°C)	$T_{d50\%}$ (°C)	T_g (°C)^a	Gel content (wt%)^b	σ ^{c} (MPa)	ε^d (%)	E^e (MPa)
1	**r-G_1**	326	382	0.2	94.7	0.7 ± 0.1	23 ± 2	4.4 ± 0.3
2	**r-S_1**	331	423	39.8	93.8	3.4 ± 0.7	200 ± 30	5 ± 4

^{a}Evaluated by DSC. ^{b}Evaluated in acetone. ^{c}Tensile strength. ^{d}Tensile strain. ^{e}Young's modulus.

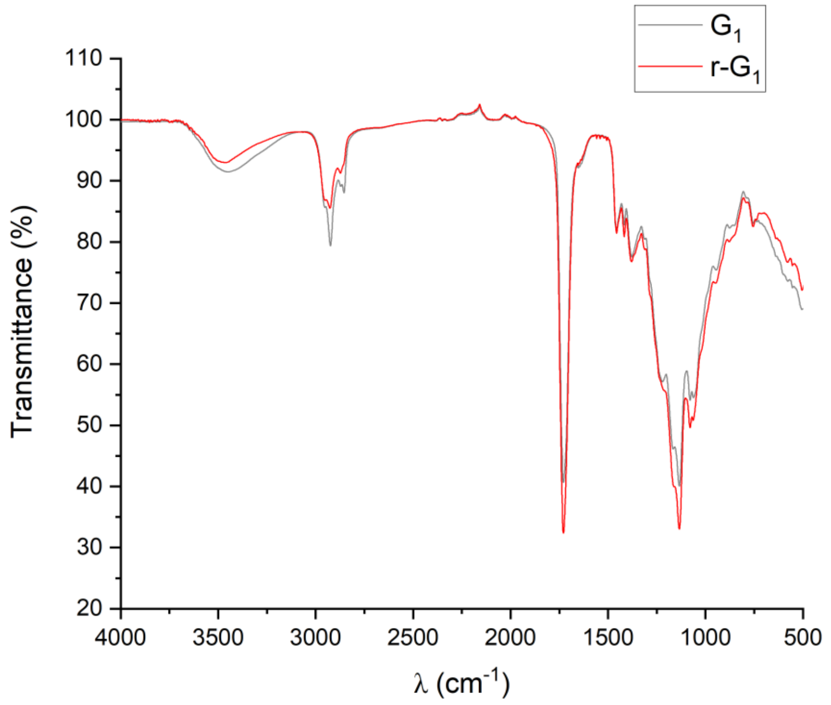

Figure S94. Superimposed normalized FT-IR spectra of fresh glycerol-based polyester (G_1) and of the chemically recycled polymer (r-G_1).

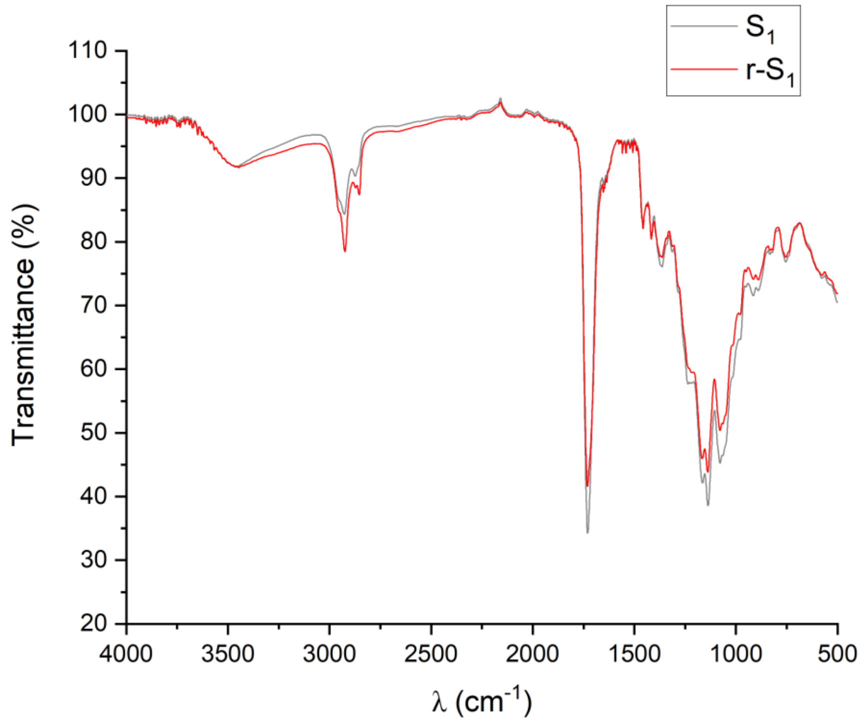

Figure S95. Superimposed normalized FT-IR spectra of fresh sorbitol-based polyester (S_1) and of the chemically recycled polymer (r-S_1).

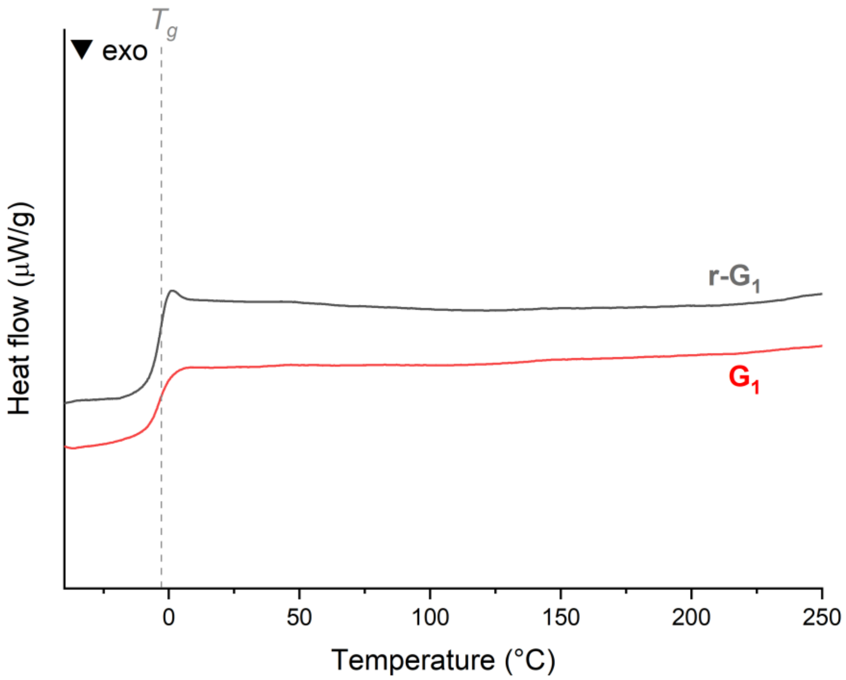

Figure S96. DSC curves of fresh glycerol-based polyester (G_1) and of the chemically recycled polymer (r-G_1).

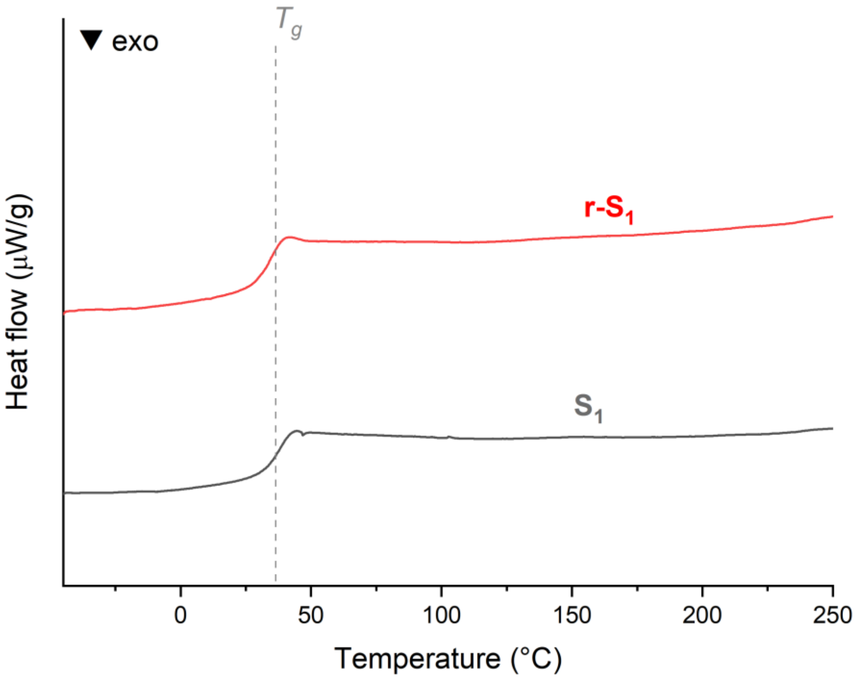

Figure S97. DSC curves of fresh sorbitol-based polyester (S_1) and of the chemically recycled polymer (r-S_1).

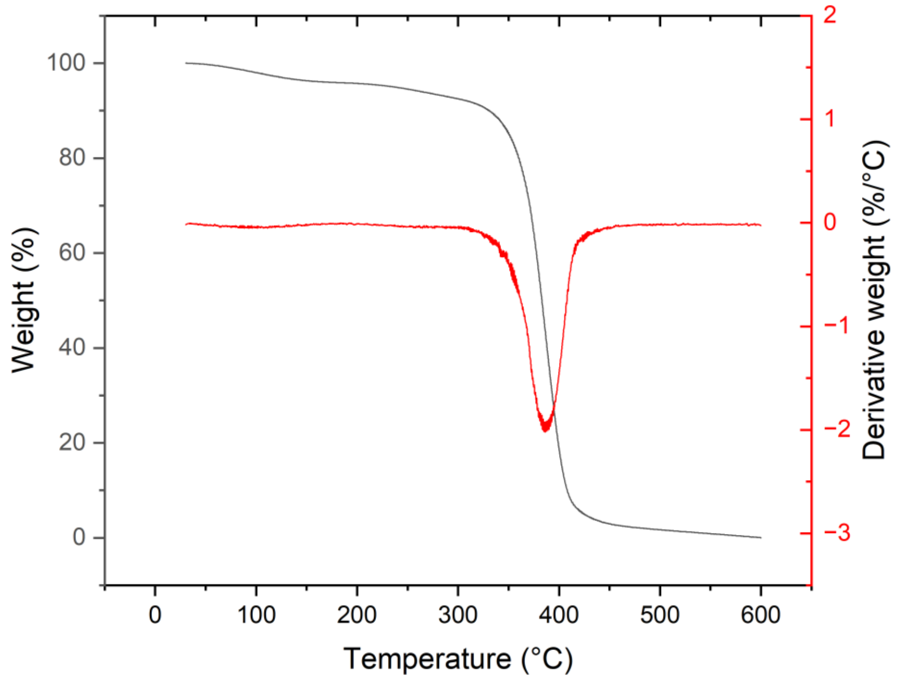

Figure S98. TGA curve of the chemically recycled glycerol-based polymer (r-G_1) in a nitrogen atmosphere.

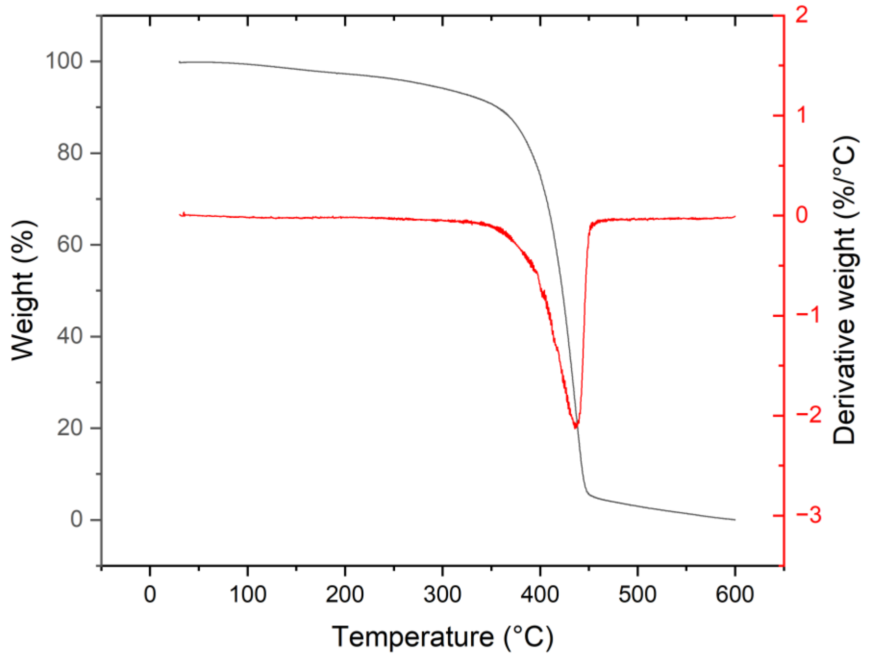

Figure S99. TGA curve of the chemically recycled sorbitol-based polymer (r-S_1) in a nitrogen atmosphere.

Thermo gravimetry analysis (TGA)

TGA was performed on a TG 209F1 instrument (NETZSCH). About 4 to 10mg of each sample was equilibrated at room temperature and heated at 10 °C min^{-1} to 600 °C under nitrogen. The reported $T_{d5\%}$ and $T_{d50\%}$ values represent the temperatures at which 5% and 50% of the mass is lost, respectively.

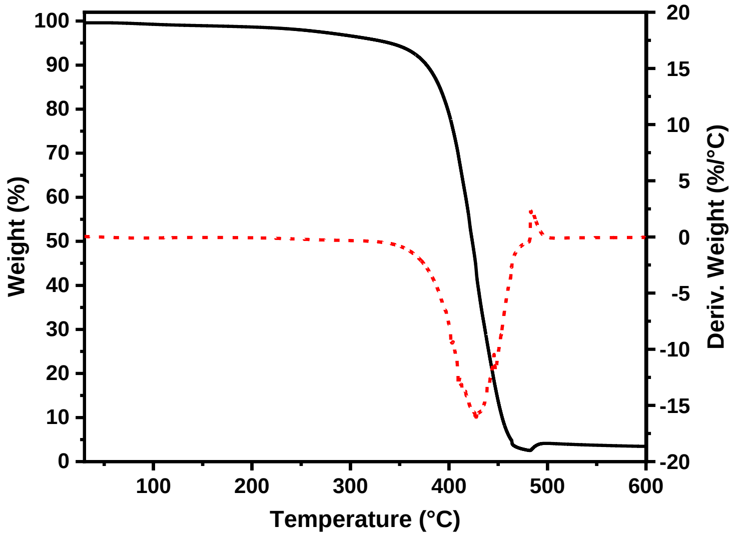

Figure S100. Weight loss (%) against temperature (°C) for sample G_1 in a nitrogen atmosphere.

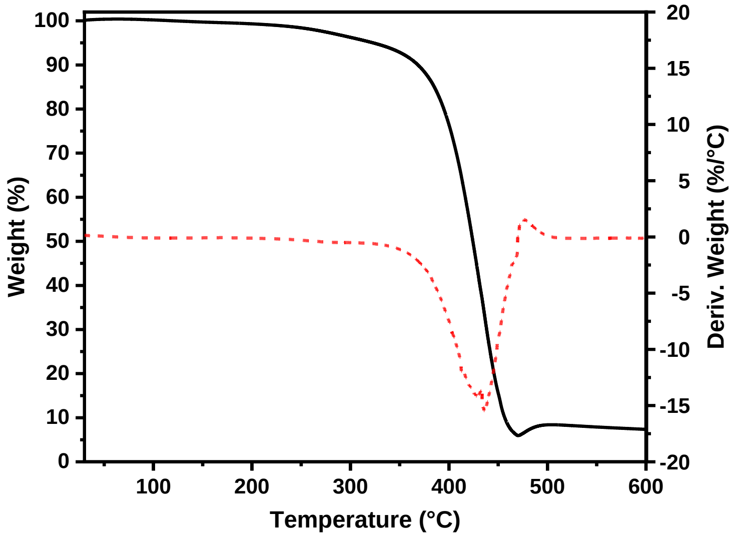

Figure S101. Weight loss (%) against temperature (°C) for sample $G_{0.5}$ in a nitrogen atmosphere.

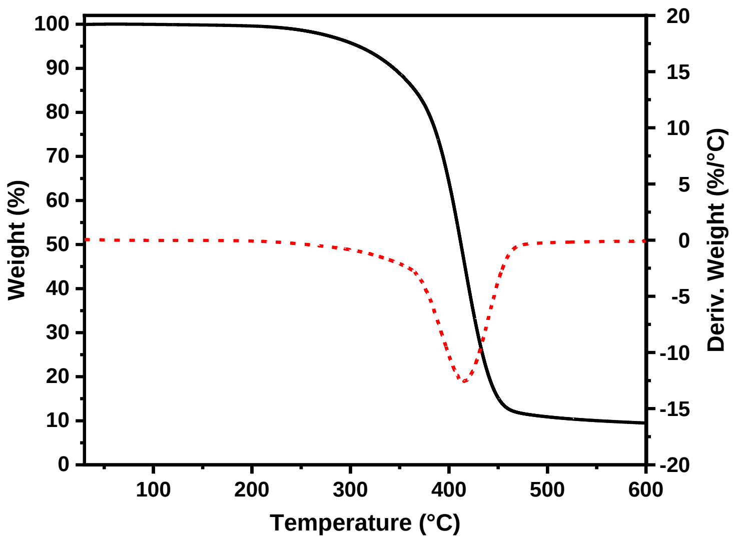

Figure S102. Weight loss (%) against temperature (°C) for sample S_1 in a nitrogen atmosphere.

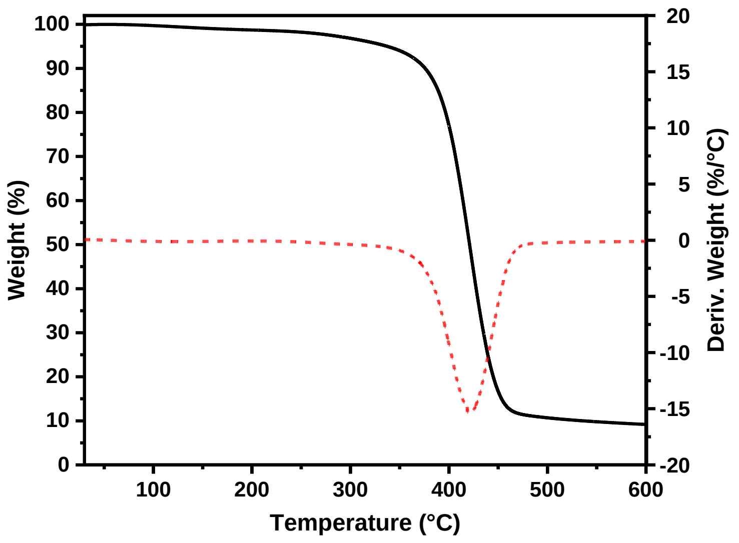

Figure S103. Weight loss (%) against temperature (°C) for sample $S_{0.5}$ in a nitrogen atmosphere.

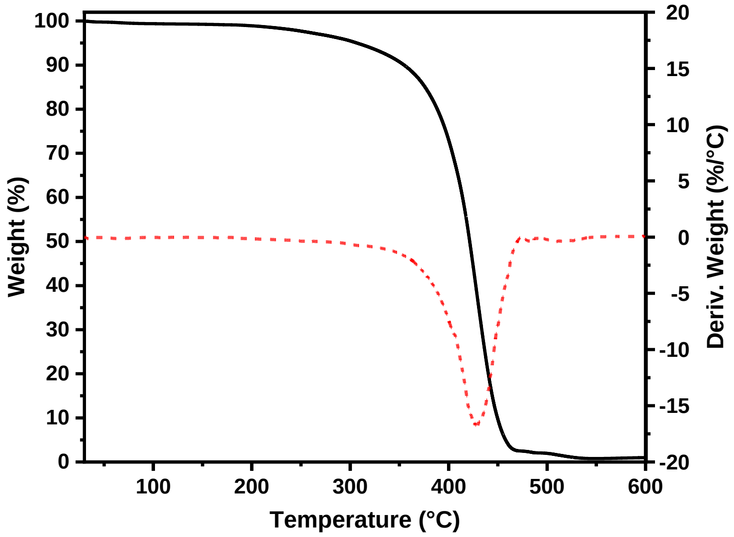

Figure S104. Weight loss (%) against temperature (°C) for sample $GS_{0.5}$ in a nitrogen atmosphere.

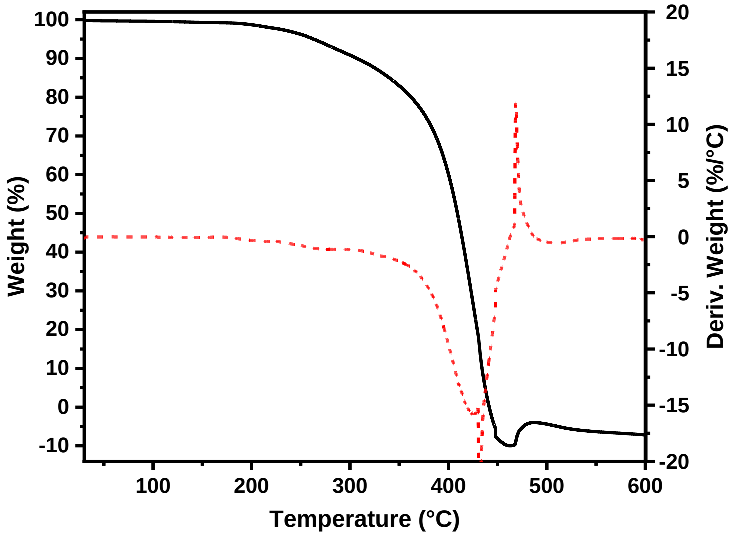

Figure S105. Weight loss (%) against temperature (°C) for sample $GS_{0.25}$ in a nitrogen atmosphere.

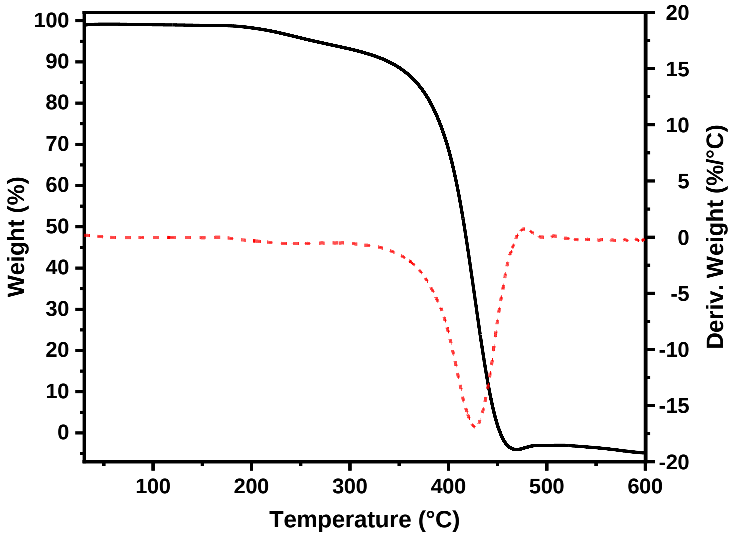

Figure S106. Weight loss (%) against temperature (°C) for sample $G_{0.2}S_{0.8}$ in a nitrogen atmosphere.

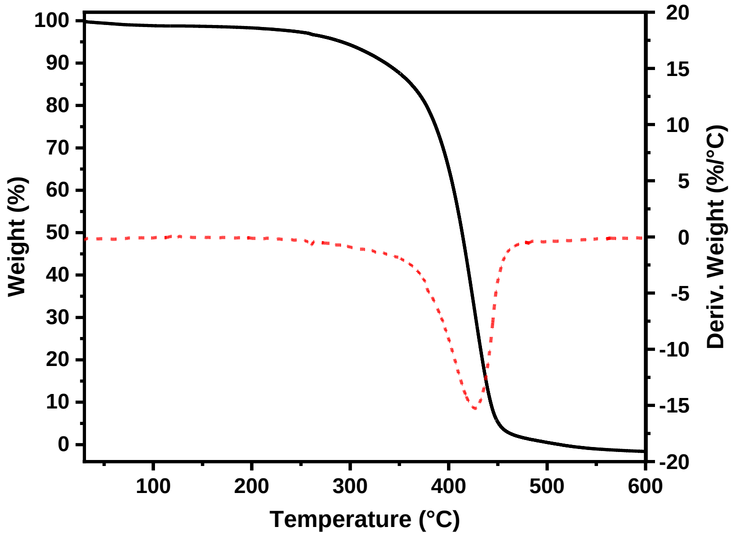

Figure S107. Weight loss (%) against temperature (°C) for sample $G_{0.8}S_{0.2}$ in a nitrogen atmosphere.

Tensile tests of the fresh materials

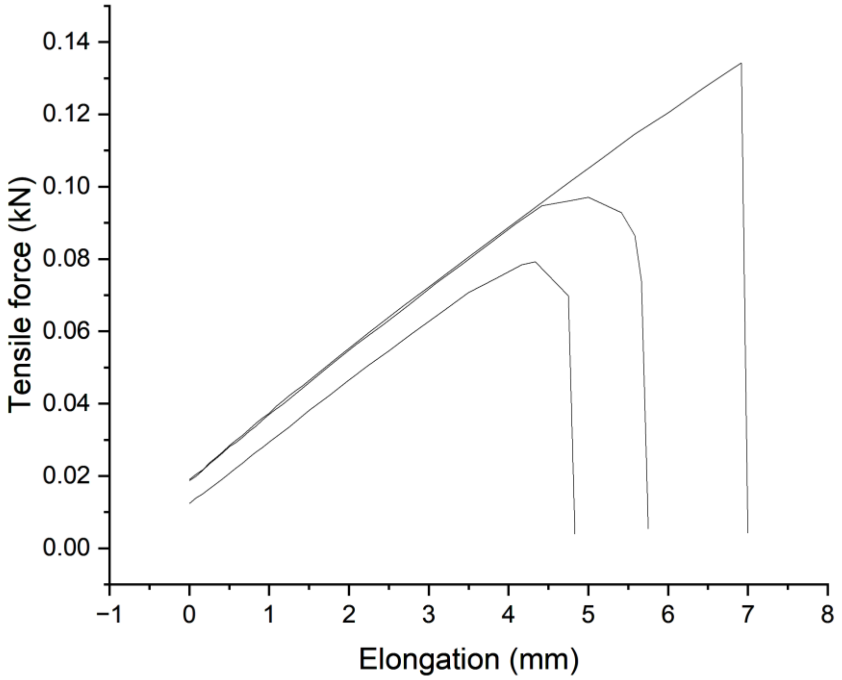

Figure 108. Stress-strain curve for sample G_1. The measurement was triplicated.

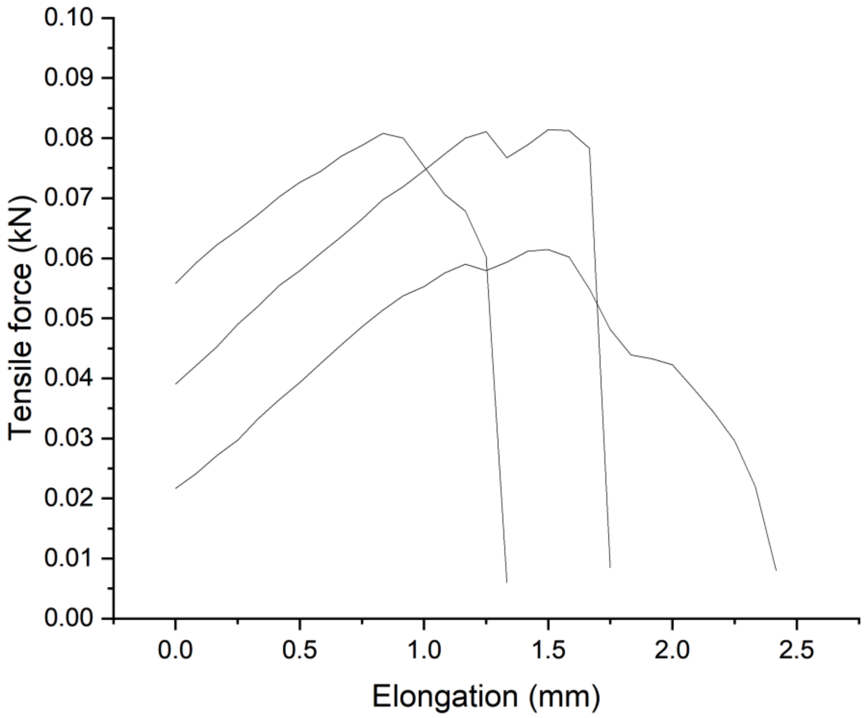

Figure S109. Stress-strain curve for sample $G_{0.5}$. The measurement was triplicated.

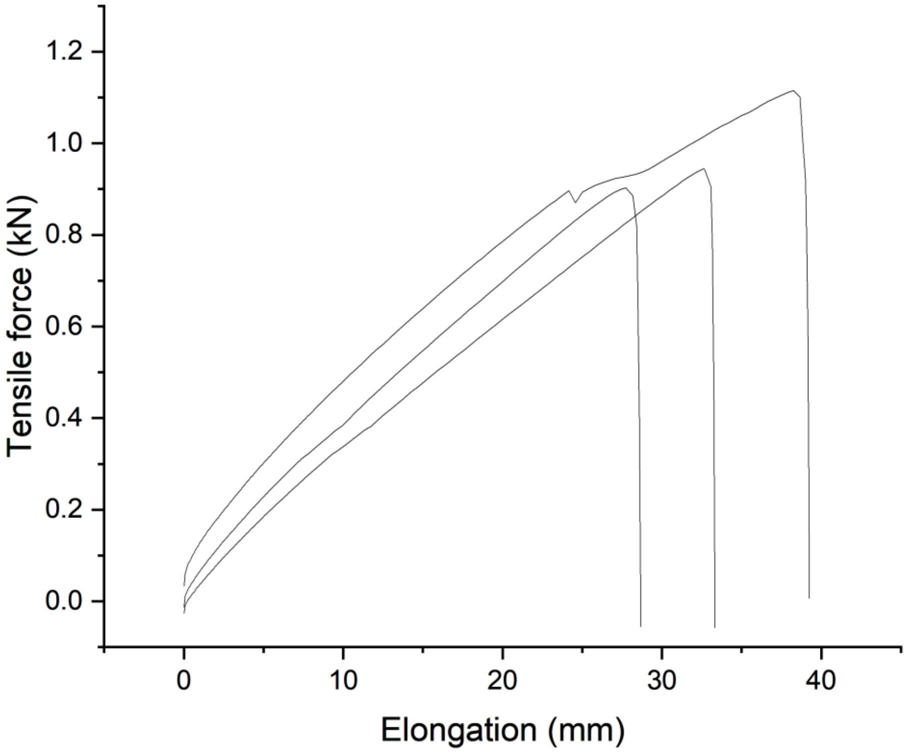

Figure S110. Stress-strain curve for sample S_1. The measurement was triplicated.

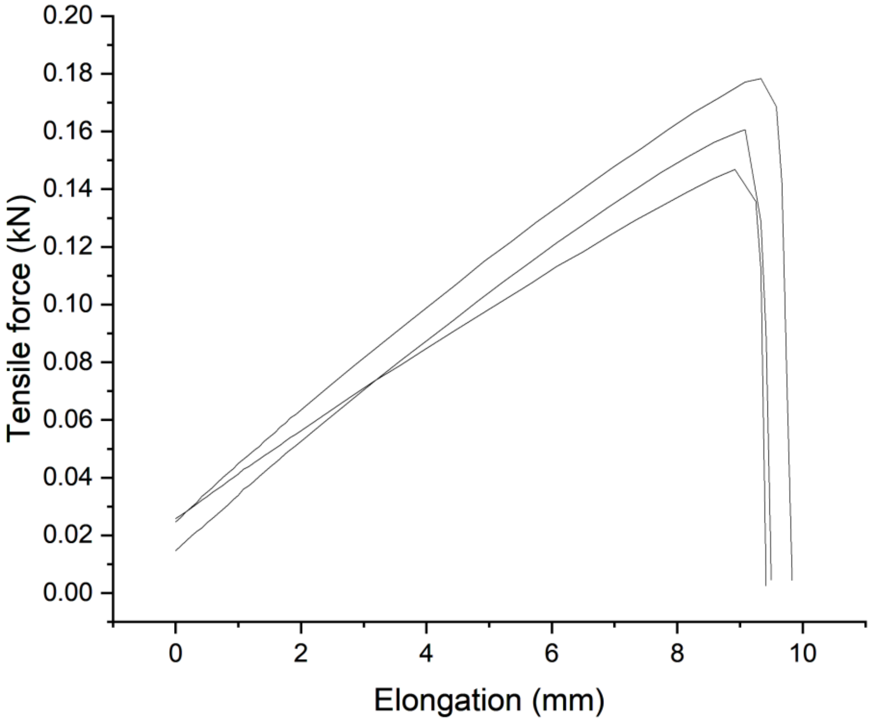

Figure S111. Stress-strain curve for sample $S_{0.5}$. The measurement was triplicated.

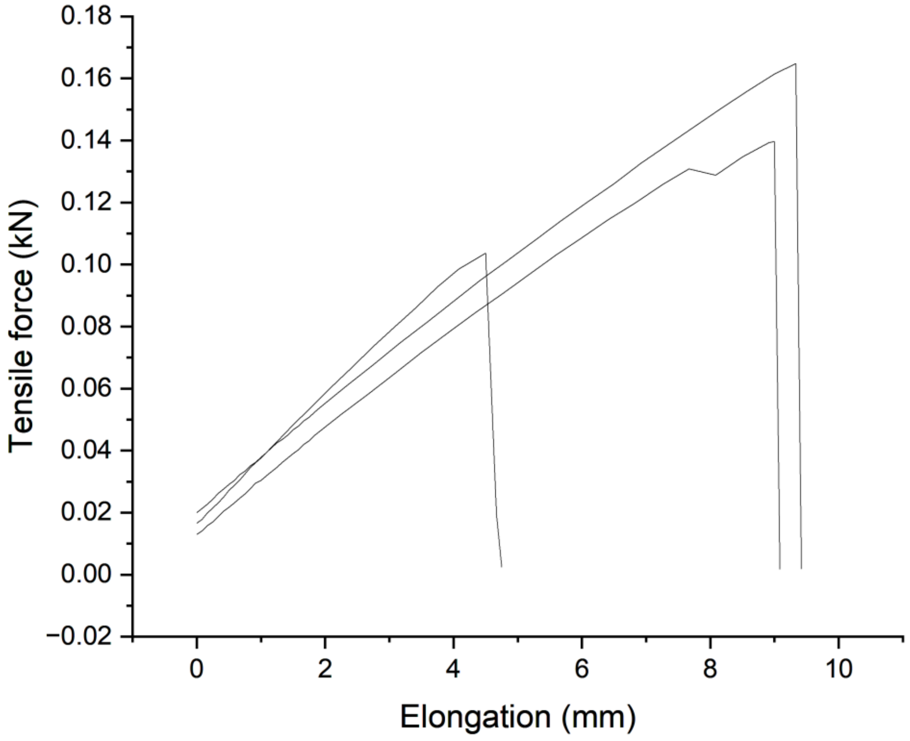

Figure S112. Stress-strain curve for sample $GS_{0.5}$. The measurement was triplicated.

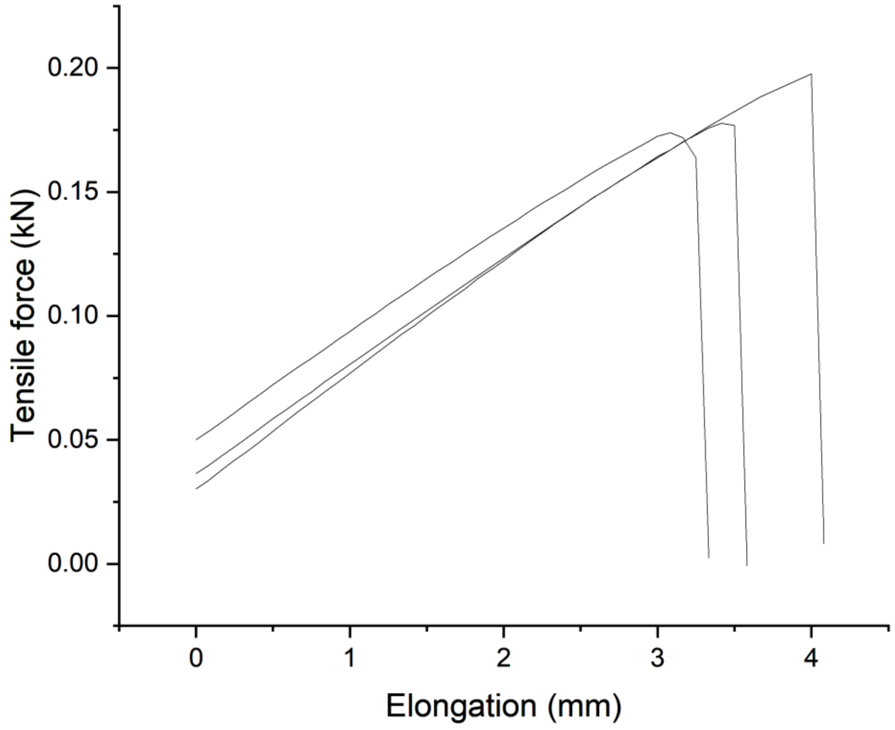

Figure S113. Stress-strain curve for sample $GS_{0.25}$. The measurement was triplicated.

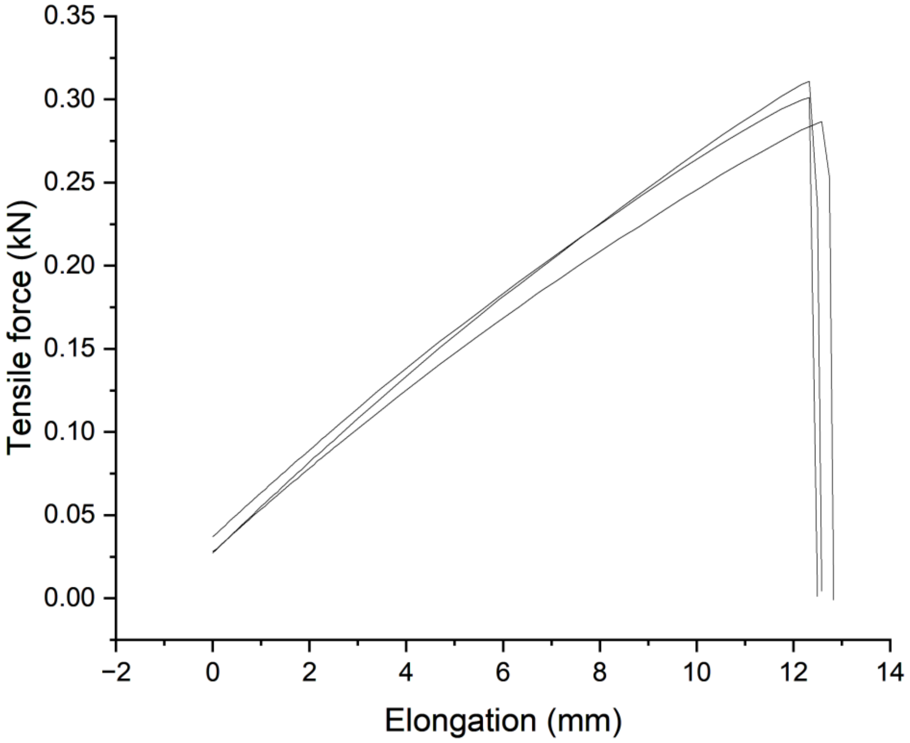

Figure S114. Stress-strain curve for sample $G_{0.2}S_{0.8}$. The measurement was triplicated.

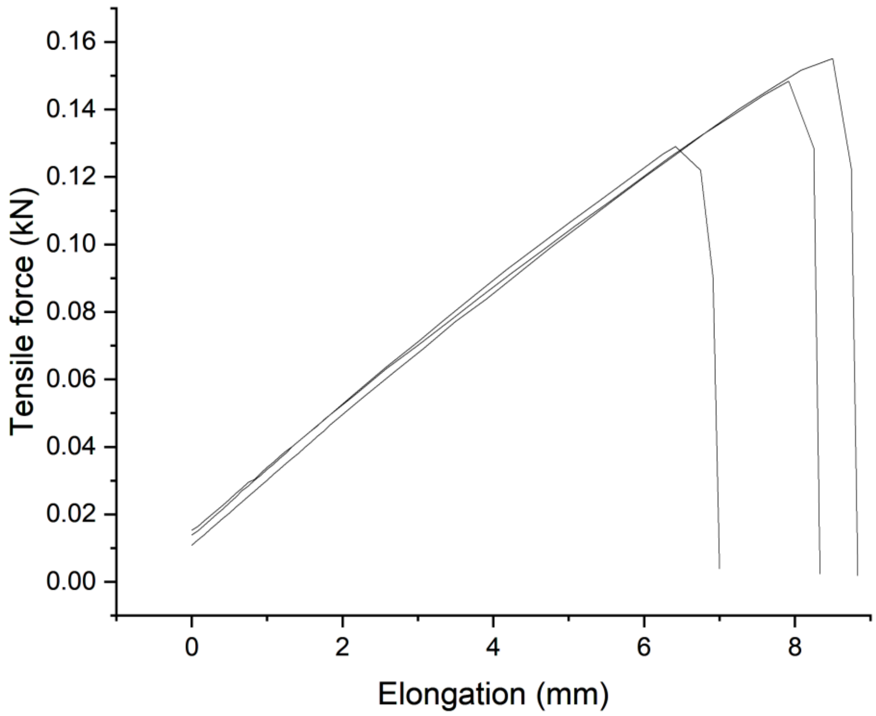

Figure S115. Stress-strain curve for sample $G_{0.8}S_{0.2}$. The measurement was triplicated.

Tensile tests of the recycled materials

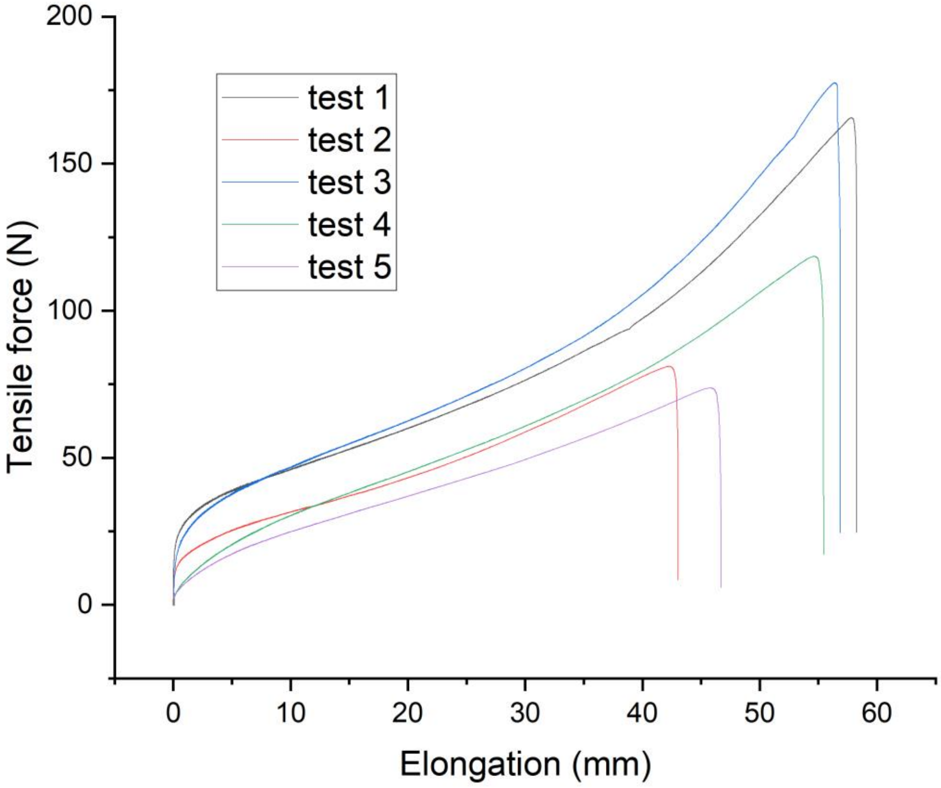

Figure S116. Stress-strain curve for sample $G_{0.2}S_{0.8}$. The measurement was carried out 5 times.

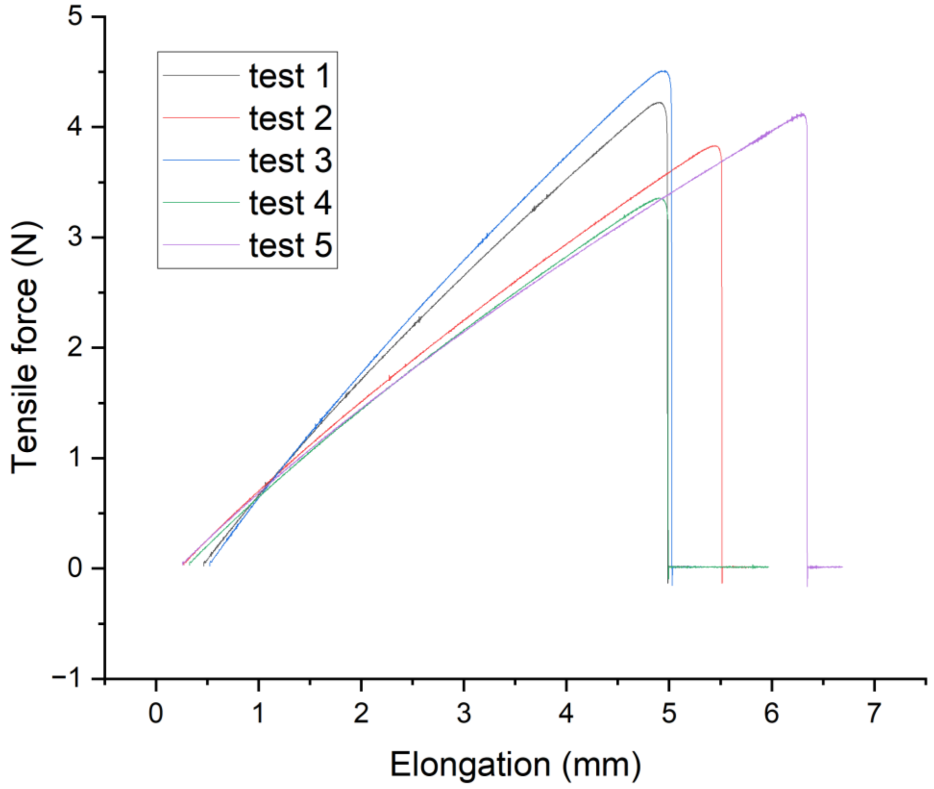

Figure S117. Stress-strain curve for sample $G_{0.8}S_{0.2}$. The measurement was carried out 5 times.

References:

[1] D. Di Francesco, D. Rigo, K. Reddy Baddigam, A. P. Mathew, N. Hedin, M. Selva, J. S. M. Samec, *ChemSusChem* **2022**, *15*, DOI 10.1002/cssc.202200326.

[2] C. R. Morcombe, K. W. Zilm, *Journal of Magnetic Resonance* **2003**, *162*, 479–486.

[3] M. de Meireles Brioude, D. Hansen Guimarães, R. da Paz Fiúza, L. Antônio Sanches de Almeida Prado, J. Soares Boaventura, N. Mamede José, *Synthesis and Characterization of Aliphatic Polyesters from Glycerol, by-Product of Biodiesel Production, and Adipic Acid*, **2007**.

[4] T. Zhang, B. A. Howell, A. Dumitrascu, S. J. Martin, P. B. Smith, *Polymer (Guildf)* **2014**, *55*, 5065–5072.

[5] J. F. Stumbé, B. Bruchmann, *Macromol Rapid Commun* **2004**, *25*, 921–924.

[6] V. Taresco, R. G. Creasey, J. Kennon, G. Mantovani, C. Alexander, J. C. Burley, M. C. Garnett, *Polymer (Guildf)* **2016**, *89*, 41–49.

[7] A. S. Kulshrestha, W. Gao, R. A. Gross, *Macromolecules* **2005**, *38*, 3193–3204.

[8] F. Zeng, X. Yang, D. Li, L. Dai, X. Zhang, Y. Lv, Z. Wei, *J Appl Polym Sci* **2020**, *137*, DOI 10.1002/app.48574.

[9] T. Wersig, M. C. Hacker, J. Kressler, K. Mäder, *Int J Pharm* **2017**, *531*, 225–234.

[10] V. Taresco, J. Suksiriworapong, R. Creasey, J. C. Burley, G. Mantovani, C. Alexander, K. Treacher, J. Booth, M. C. Garnett, *J Polym Sci A Polym Chem* **2016**, *54*, 3267–3278.

[11] P. Kallinteri, S. Higgins, G. A. Hutcheon, C. B. St. Pourçain, M. C. Garnett, *Biomacromolecules* **2005**, *6*, 1885–1894.

[12] L. Navarro, N. Ceaglio, I. Rintoul, *Polym J* **2017**, *49*, 625–632.

[13] W. Q. Yuan, G. L. Liu, C. Huang, Y. D. Li, J. B. Zeng, *Macromolecules* **2020**, *53*, 9847–9858.

[14] A. R. Goddard, E. A. Apebende, J. C. Lentz, K. Carmichael, V. Taresco, D. J. Irvine, S. M. Howdle, *Polym Chem* **2021**, *12*, 2992–3003.

[15] A. Anand, R. D. Kulkarni, C. K. Patil, V. V. Gite, *RSC Adv* **2016**, *6*, 9843–9850.

[16] L. Gustini, B. A. J. Noordover, C. Gehrels, C. Dietz, C. E. Koning, *Eur Polym J* **2015**, *67*, 459–475.

[17] L. Gustini, C. Lavilla, W. W. T. J. Janssen, A. Martínez De Ilarduya, S. Muñoz-Guerra, C. E. Koning, *ChemSusChem* **2016**, *9*, 2250–2260.

[18] A. Anand, R. D. Kulkarni, V. V. Gite, in *Prog Org Coat*, **2012**, pp. 764–767.

[19] V. Kavimani, V. Jaisankar, *Journal of Physical Science and Application* **2014**, *4*, 507–515.

[20] A. V. Kavimani, B. Viswanathan Jaisankar, *Indian Journal of Advances in Chemical Science Design, Synthesis and Characterization of Biodegradable Sorbitol Elastomers with Tunable Mechanical and Degradation Properties*, **2015**.

[21] A. Kumar, A. S. Kulshrestha, W. Gao, R. A. Gross, *Macromolecules* **2003**, *36*, 8219–8221.

[22] H. Fu, A. S. Kulshrestha, W. Gao, R. A. Gross, M. Baiardo, M. Scandola, *Macromolecules* **2003**, *36*, 9804–9808.

[23] D. Rigo, R. Calmanti, A. Perosa, M. Selva, *Green Chemistry* **2020**, *22*, 5487–5496.

[24] D. Rigo, A. F. Masters, T. Maschmeyer, M. Selva, G. Fiorani, *Chemistry – A European Journal* **2022**, DOI 10.1002/chem.202200431.

[25] H. Wang, N. D. Vu, G. R. Chen, E. Métay, N. Duguet, M. Lemaire, *Green Chemistry* **2021**, *23*, 1154–1159.

[26] J. M. Lowe, B. E. Bowers, Y. Seo, M. R. Gagné, *Angewandte Chemie - International Edition* **2020**, *59*, 17297–17300.

[27] Y. Xiu, A. Chen, X. Liu, C. Chen, J. Chen, L. Guo, R. Zhang, Z. Hou, *RSC Adv* **2015**, *5*, 28233–28241.

[28] P. Che, F. Lu, X. Si, H. Ma, X. Nie, J. Xu, *Green Chemistry* **2018**, *20*, 634–640.

[29] N. M. Hein, Y. Seo, S. J. Lee, M. R. Gagné, *Green Chemistry* **2019**, *21*, 2662–2669.
